# A Comprehensive Analysis of the Metal–Nitrile Bonding in an Organo-Diiron System

**DOI:** 10.3390/molecules26237088

**Published:** 2021-11-23

**Authors:** Giulio Bresciani, Lorenzo Biancalana, Guido Pampaloni, Stefano Zacchini, Gianluca Ciancaleoni, Fabio Marchetti

**Affiliations:** 1 Department of Chemistry and Industrial Chemistry, University of Pisa, Via G. Moruzzi 13, I-56124 Pisa, Italy; giulio.bresciani@dcci.unipi.it (G.B.); lorenzo.biancalana@unipi.it (L.B.); guido.pampaloni@unipi.it (G.P.); 2 Interuniversity Consortium for Chemical Reactivity and Catalysis (CIRCC), Via Celso Ulpiani 27, I-70126 Bari, Italy; stefano.zacchini@unibo.it; 3 Department of Industrial Chemistry “Toso Montanari”, University of Bologna, Viale Risorgimento 4, I-40136 Bologna, Italy

**Keywords:** coordination chemistry, nitrile ligand, metal–nitrile bonding, π-back-donation, diiron complexes, DFT calculations

## Abstract

Nitriles (N≡CR) are ubiquitous in coordination chemistry, yet literature studies on metal–nitrile bonding based on a multi-technique approach are rare. We selected an easily-available di-organoiron framework, containing both π-acceptor (CO, aminocarbyne) and donor (Cp = η^5^−C_5_H_5_) ligands, as a suitable system to provide a comprehensive description of the iron–nitrile bond. Thus, the new nitrile **(2–12)CF_3_SO_3_** and the related imine/amine complexes **(8–9)CF_3_SO_3_** were synthesized in 58–83% yields from the respective tris-carbonyl precursors **(1a–d)CF_3_SO_3_**, using the TMNO strategy (TMNO = trimethylamine-N-oxide). The products were fully characterized by elemental analysis, IR (solution and solid state) and multinuclear NMR spectroscopy. In addition, the structures of **(2)CF_3_SO_3_**, **(3)CF_3_SO_3_**, **(5)CF_3_SO_3_** and **(11)CF_3_SO_3_** were ascertained by single crystal X-ray diffraction. Salient spectroscopic data of the nitrile complexes are coherent with the scale of electron-donor power of the R substituents; otherwise, this scale does not match the degree of Fe → N π-back-donation and the Fe–N bond energies, which were elucidated in **(2–7)CF_3_SO_3_** by DFT calculations.

## 1. Introduction

Nitriles (N≡CR) are ubiquitous and versatile ligands in coordination chemistry [1,2,3]. They most commonly behave as monodentate (*end-on*) ligands, and have been widely employed as weakly coordinating agents in complexes of low- to middle-valent transition metals, since their substitution by more strongly coordinating ligands is a convenient strategy to access a multitude of derivatives, catalysts and materials [4,5,6,7,8,9,10,11]. Furthermore, nitrile ligands are usually susceptible to nucleophilic attack [12,13,14] and may be engaged in a great variety of chemical transformations mediated by the adjacent metal centre, either catalytic [15,16,17,18] or stoichiometric [12,19,20,21,22,23].

**Scheme 1:**
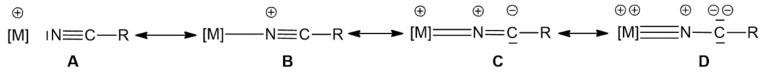
Resonance structures contributing to a generic metal–nitrile bond.

In principle, the metal–nitrile bond can be described in terms of four resonance structures (Scheme 1).

Structure **A** accounts for a purely electrostatic interaction: the metal attracts the nitrile electrons (both σ and π), thus strengthening the triple N≡C bond with a polarization toward the nitrogen (N ← C). Structure **B** represents the σ metal–nitrile bond, where the polarization is still N ← C (bond strengthening) because of the formal charge on the nitrogen. Structure **C** shows the back-donation of an electron pair from the metal to one π * orbital of the nitrile. In this case, the polarization is N → C and thus the N≡C bond is weakened. As two empty π * orbitals are available on the nitrile, the metal may back-donate an additional electron pair, leading to the limit structure **D**, wherein the N → C polarization and the carbon–nitrogen bond weakening reach their extreme.

In most literature cases, *end-on* nitriles have been regarded as essentially σ-donor ligands, with a possible secondary contribution to the bond with the metal arising from π-back-donation [1,24,25]. Pombeiro placed nitriles and phosphines approximately on the same level of a scale of *net* π-*electron acceptor minus* σ-*donor character*, based on electrochemical studies [26,27,28].

**Figure 1:**
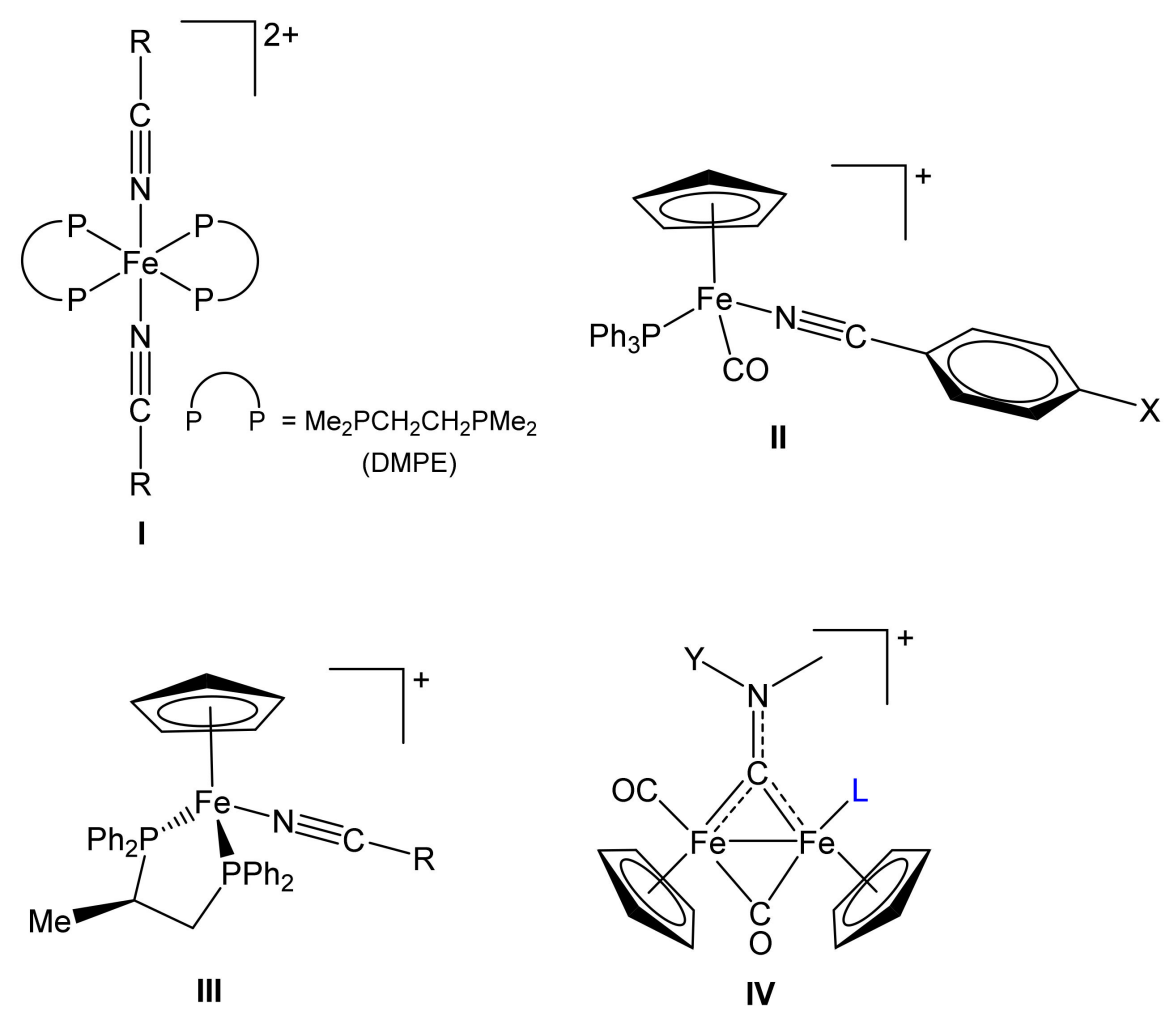
Iron–nitrile complexes cited in the Introduction. (**I**) R = Me, Ph, 4-C_6_H_4_Br [13]. (**II**) X = H, OH, CH_2_OH, NH_2_, Br, Cl [15]. (**III**) R = Et, Ph, 4-C_6_H_4_Me, 4-C_6_H_4_OMe, 4-C_6_H_4_NMe_2_, 4-C_6_H_4_NO_2_ [16]. (**IV**) Diiron complexes with a bridging aminocarbyne ligand (Y = alkyl or aryl; L = CO, replaceable with a nitrile).

In several cases, IR data have been invoked to proof the occurrence of metal to nitrile π-back-donation. While the stretching wavenumber of the N≡C bond (ῦ_N__≡C_) is often quite near to that of the uncoordinated nitrile, or even higher [29], a wavenumber decrease upon coordination has been imputed to π-back-donation. This feature was prevalently observed with arylnitriles [24,30,31,32], and less frequently with acetonitrile, cyanoacetic acid (N≡CCH_2_CO_2_H) and acrylonitrile (N≡CCH=CH_2_) [33]. For instance, Skelton and co-workers prepared a series of mono- and bis-nitrile iron derivatives (Figure 1, structure **I**) [34], and observed a minor change of ῦ_N__≡C_ (N≡CCH_3_ ligand) with respect to free acetonitrile, whereas a decrease by 10–30 cm^−1^ was recognized with aryl-nitriles. In addition, the X-ray structures displayed a shorter Fe–N distance in [Fe(DMPE)_2_{NC(4-C_6_H_4_Br)}_2_]^2+^ [1.893(7)Å] than in [Fe(DMPE)_2_{NCPh}_2_]^2+^ [1.917(5)Å] (DMPE = 1,2-bis(dimethylphosphino)ethane), attributed to a higher degree of back-donation in the former, due to the electron-withdrawing effect of the Br-substituent. Conversely, X-ray data related to a series of [RuCl(NCR)(DPPM)_2_]^+^ complexes (DPPM = Ph_2_PCH_2_PPh_2_) with alkyl-nitriles revealed almost identical distances for the Ru-P bonds—trans to the chloride and trans to the nitrile, respectively—thus arguing against any significant Ru to NCR π-back-donation [35].

Piano-stool iron complexes of the type [FeCp(CO){NC(4-C_6_H_4_X)}(PPh_3_)]^+^ (Figure 1, structure **II**) manifested electronic effects (IR and NMR data) related to the aryl substituent (X), which were tentatively explained with an increase of Fe to N back-donation on going along the series X = OH < H < Br [36].

The effect of the nitrile substituent (R) on the strength of the metal–nitrile bond was elegantly demonstrated on the series of complexes [FeCp(Prophos)(NCR)]PF_6_ (Figure 1, structure **III**); Prophos = (R)-(+)-1,2-bis(diphenylphosphino)propane), by means of a kinetic study in CDCl_3_ solution [37]. These complexes contain two chiral centres, and the rate-determining step for the first-order epimerization reaction is the cleavage of the iron–nitrile bond. Therefore, the following sequence of stability was established on account of the half-life times of complexes; i.e., NCMe < NCEt < NCPh < NC(4-C_6_H_4_Me) < NC(4-C_6_H_4_NMe_2_).

Casarin and co-workers estimated the π-backbonding in [PtCl_2_(NCR)_2_] adducts as 30–40% of the total Pt–N bonding interaction, and pointed out the negligible effect of the R group; these authors concluded that ῦ_N__≡C_ values are not correlated with the strength of the nitrile bond [38]. Back-donation was investigated by DFT also on other metal–nitrile systems [27,39,40,41].

In this scenario, a comprehensive description of metal–nitrile bonding, embracing crystallographic, spectroscopic and computational methods, is scarcely available. Our long experience with the chemistry of diiron μ–aminocarbyne complexes (Figure 1, structure **IV**) [42,43,44] prompted us to exploit such a versatile and easily-accessible molecular framework to elucidate the iron–nitrile bonding picture [4]. With this purpose in mind, the considered system offers some advantageous features: (1) the effectiveness of the nitrile substituent is suggested by previous findings, according to which the N≡CR ligand undergoes addition by anionic nucleophiles [45,46,47], leading to different outcomes depending on R [46,47,48]; (2) the presence of two types of π-acceptor co-ligands, i.e., the carbonyls and one (variable) aminocarbyne, amplifies the possibility of evaluating the electronic influence of R; (3) in principle, the formal +I oxidation state of the iron centres might enable an appreciable iron to nitrile back-donation despite the net cationic charge of the complex.

Trends of experimental data from our multi-technique approach will be examined in detail; we will discuss their correlation with the electronic properties of the nitrile substituents and with the degree of π-back-donation and the Fe–N bond energies, which have been estimated by DFT in the distinct cases.

## 2. Results and Discussion

### 2.1. Synthesis of Diiron μ-Aminocarbyne Complexes with Nitrile- and Other Nitrogen-Ligands

**Figure 2:**
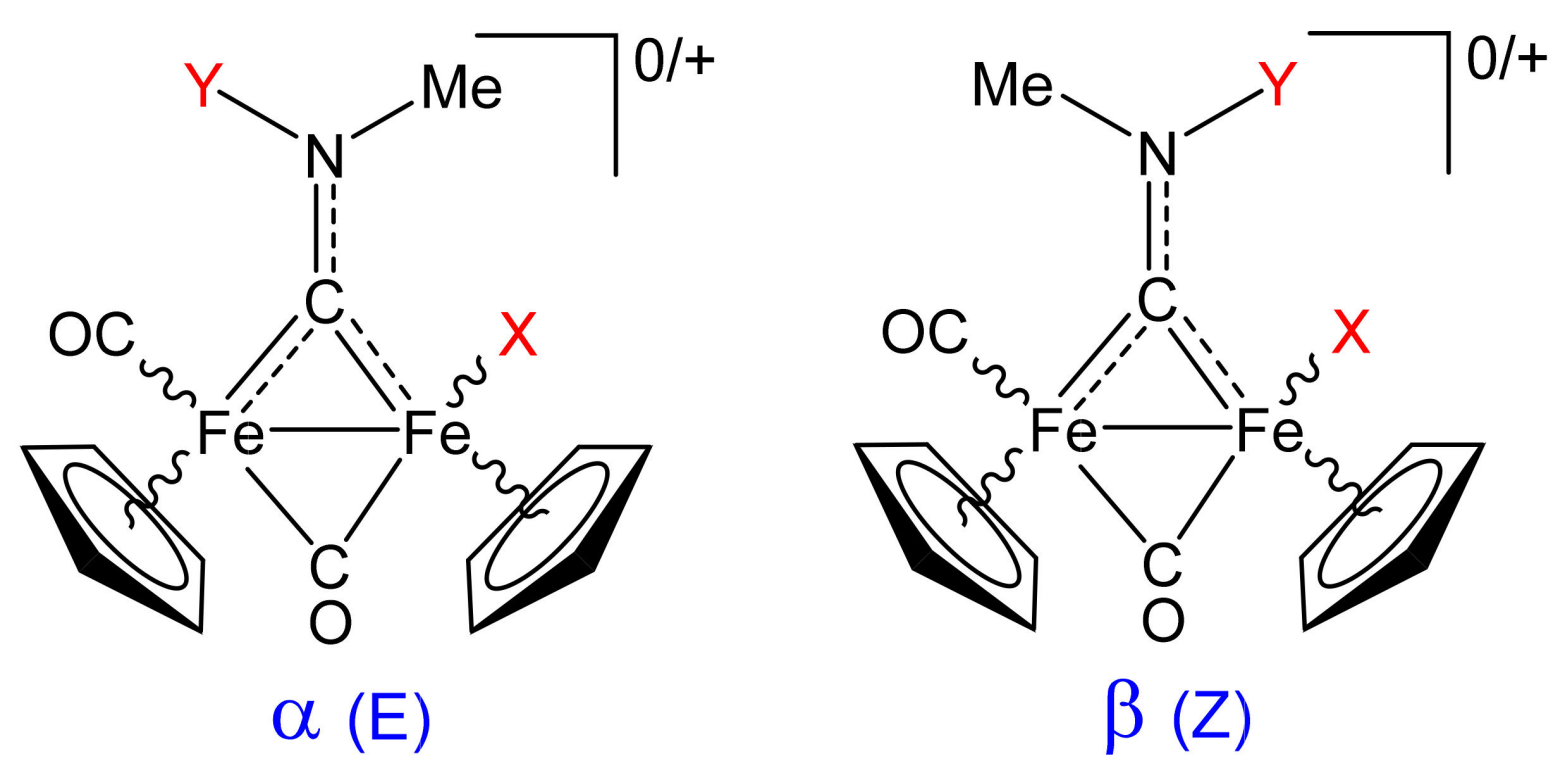
Isomerism in asymmetric diiron μ–aminocarbyne complexes (Cp rings in cis or trans position). Y = CH_2_Ph or 2,6-C_6_H_3_Me_2_. X = Ph, alkyl, dithiocarbamate, halide/pseudohalide (neutral complexes); X = nitrile, isocyanide, phosphine, imine, carbene (cationic complexes). When X = nitrile, α and β correspond to E and Z isomers, respectively.

**Scheme 2:**
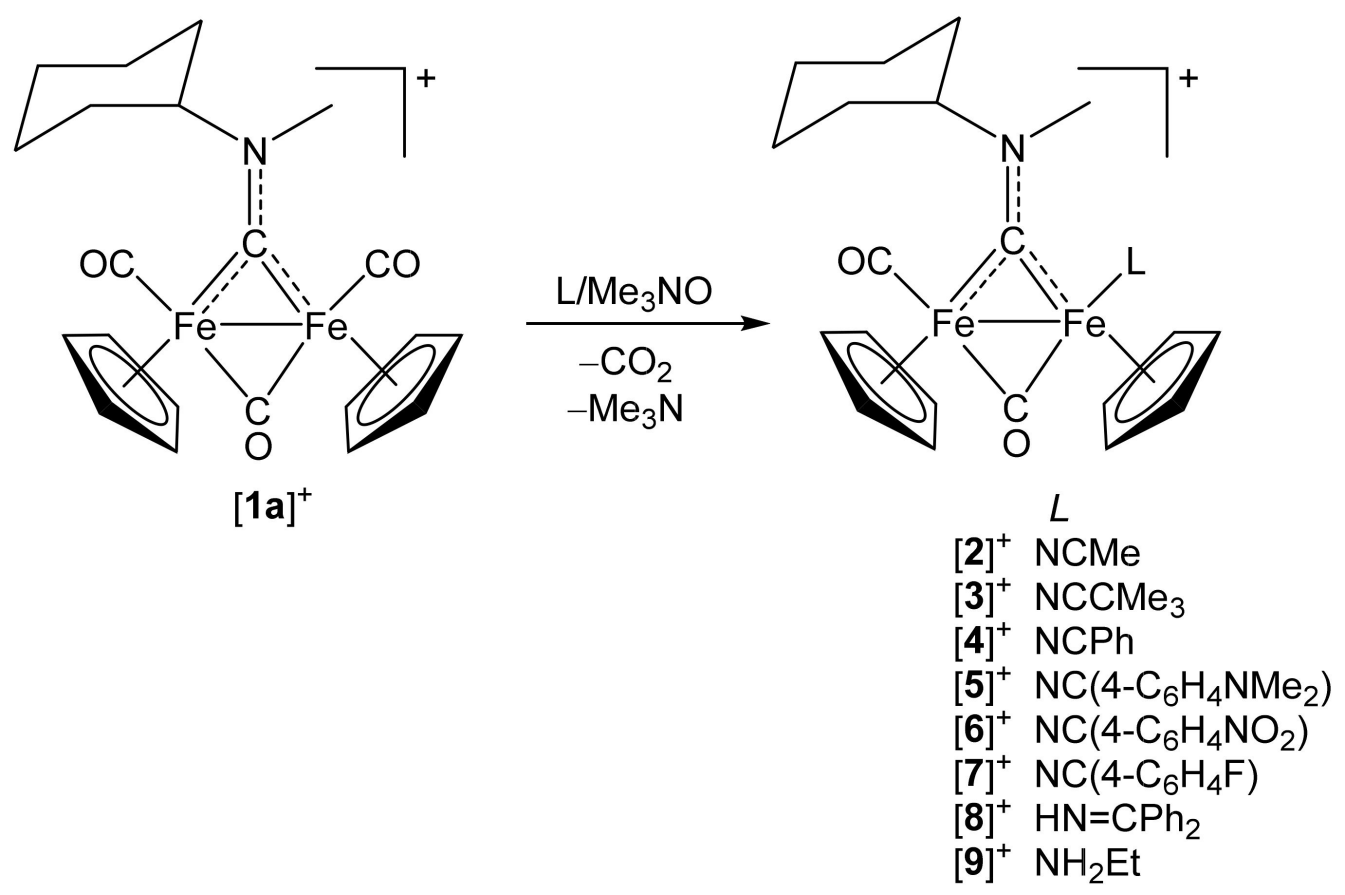
Carbonyl-substitution reactions on a (*N*-methyl, *N*-cyclohexyl)aminocarbyne diiron complex (CF_3_SO_3_^−^ salts).

**Scheme 3:**
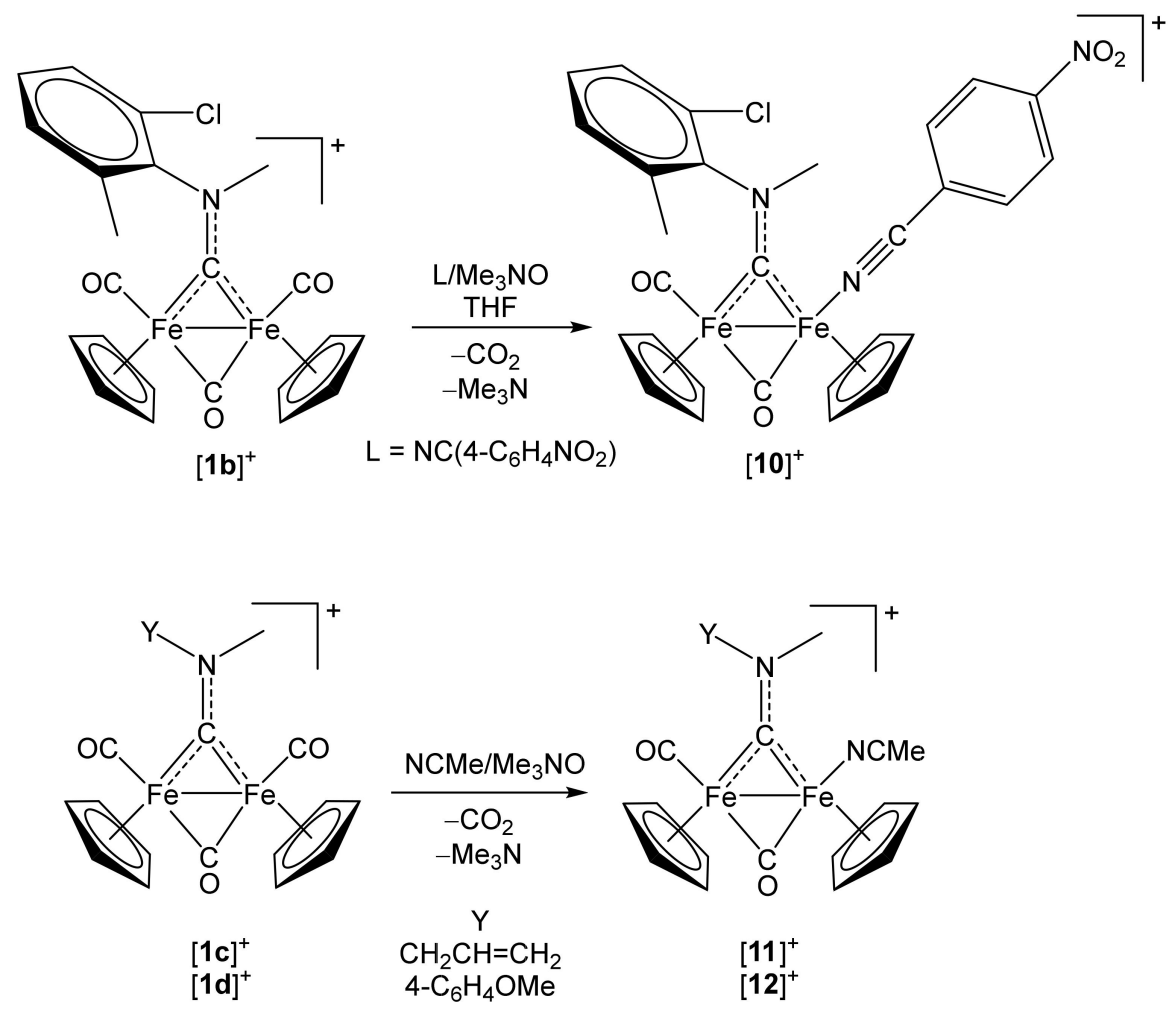
Synthesis of nitrile derivatives from complexes with different aminocarbyne substituents (CF_3_SO_3_^−^ salts).

The triflate salt [Fe_2_Cp_2_(CO)_2_(µ-CO){µ-CN(Me)(Cy)}]CF_3_SO_3_ (**(1a)CF_3_SO_3_**) [43] was selected as a starting material, bearing in mind that two aminocarbyne substituents of considerably different size (i.e., methyl and Cy = cyclohexyl) could supply information about steric factors related to nitrile coordination. More precisely, the aminocarbyne group possesses some iminium character, whereby the rotation around the carbyne–nitrogen bond is inhibited at room temperature; therefore, the replacement of one CO with a nitrile ligand may result in the formation of two isomers with a ratio depending on the relative hindrance of nitrile and Y (Figure 2), vide infra. The novel complexes **(2–7)CF_3_SO_3_** were prepared from the reactions of **(1a)CF_3_SO_3_** with the appropriate nitrile reactant, in the presence of a slight excess of Me_3_NO∙2H_2_O (Scheme 2). The products were purified by alumina chromatography and finally isolated as air-stable solids in 69–83% yields. By using a similar procedure, the new complexes **(8)CF_3_SO_3_** and **(9)CF_3_SO_3_**, containing respectively an imine and an amine as monodentate N-donor ligands, were also synthesized for comparative purposes (58–62% yields, Scheme 2). Moreover, in order to evaluate the possible effect of the aminocarbyne substituents on iron–nitrile bonding, complexes **(10–12)CF_3_SO_3_** were obtained from the respective tris-carbonyl precursors, **(1b–d)CF_3_SO_3_**, in 64–77% yields (Scheme 3).

All the products were fully characterized by means of elemental analysis, IR, ^1^H and ^13^C NMR spectroscopy (Figures S1–S47): the salient spectroscopic features are visualized in Table 1. In addition, the structures of **(2)CF_3_SO_3_**, **(3)CF_3_SO_3_**, **(5)CF_3_SO_3_** and **(11)CF_3_SO_3_** were ascertained by single crystal X-ray diffraction studies, and nitrile complexes **(2–7)CF_3_SO_3_** underwent DFT investigation. Spectroscopic, X-ray and DFT data will be discussed in the following sections.

### 2.2. Analysis of IR Spectra

**Scheme 4:**
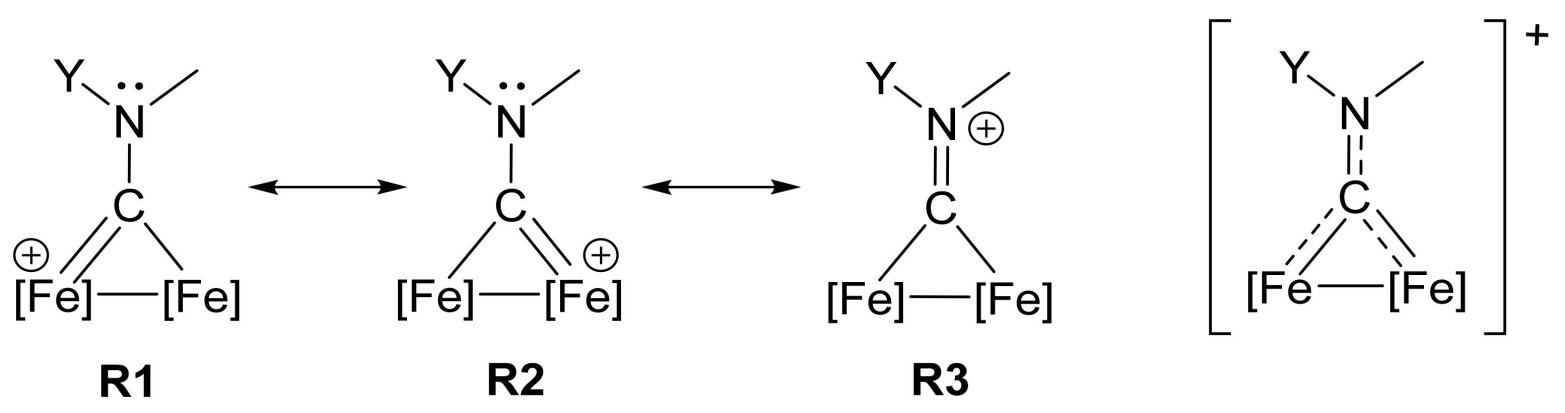
Resonance structures representing the aminocarbyne (**R1**, **R2**) and iminium (**R3**) character of the bridging hydrocarbyl ligand in (**2–12**)^+^, and combined representation with delocalized charge.

IR spectra were recorded both in dichloromethane solution and in the solid state. They share a common pattern consisting of three main absorptions in the 2300–1500 cm^−1^ region, ascribable to the terminal and bridging carbonyls and the carbyne–nitrogen bond; isomers (see Section 3) are not distinguishable. Data indicate that the µ-(C–N) bond possesses some double bond character (iminium character), as usually found in related compounds (Scheme 4) [42,51,52]. The nature of the nitrile has negligible influence on the CO, µ-CO and µ-CN stretching vibrations which, for compounds **(2–7)CF_3_SO_3_**, fall within the narrow intervals 1982–1985, 1818–1821 and 1559–1561 cm^−1^, respectively (in CH_2_Cl_2_). Complexes **(8,9)CF_3_SO_3_** display significantly lower values for the CO wavenumbers, thus pointing out the stronger electron-donor power of ethylamine and, to a less extent, benzophenone imine with respect to the investigated nitriles. The relatively high electron donation supplied by NH_2_Et also enhances the metal-to-carbyne back-donation (Scheme 4, resonance structures **R1** and **R2**) with consequent weakening of the µ-C-N bond (ῦ = 1532 cm^−1^).

The nitrile N≡C stretching gives rise to a weak absorption in the 2230–2280 cm^−1^ region. The variations of wavenumber (Δῦ) of the nitrile stretching in the complexes with respect to the corresponding uncoordinated nitriles are compiled in Table 1, with reference to both dichloromethane solutions and solid state. Δῦ values decrease along the series (**3**)^+^ > (**2**)^+^ > (**5**)^+^ > (**4**)^+^ > (**7**)^+^ > (**6**)^+^, in accordance with the progressive decrease of the electron-donor ability of the R substituent (^t^Bu > Me > 4-C_6_H_4_NMe_2_ > C_6_H_5_ > 4-C_6_H_4_F > 4-C_6_H_4_NO_2_), and are all slightly positive except for the 4-nitrobenzonitrile complexes (**6**)^+^ and (**10**)^+^. Moreover, Δῦ for the acetonitrile complexes varies according to the sequence (**12**)^+^ > (**2**)^+^ > (**11**)^+^, suggesting that the π-acceptor potential of NCMe ligand is affected by the aminocarbyne substituents, and decreases along the series Y = allyl > cyclohexyl > 4-methoxyphenyl. Note that the same sequence is reflected in the ῦ (μ-CN) values ((**12**)^+^ < (**2**)^+^ < (**11**)^+^).

Similarly, Δῦ is lower in (**6**)^+^ (R = 4-C_6_H_4_NO_2_, Y = Cy) than in (**10**)^+^ (R = 4-C_6_H_4_NO_2_, Y = 2,6-C_6_H_3_MeCl), because of the superior electron-donor character of the cyclohexyl aminocarbyne substituent compared to 2-chloro-6-methylphenyl, favouring in (**6**)^+^ back-donation from the iron to 4-nitrobenzonitrile, at the expense of the aminocarbyne.

### 2.3. Analysis of NMR Spectra

As expected, the NMR spectra of **(2–12)CF_3_SO_3_** reveal the existence in solution of E-Z isomers, with reference to the different orientations assumed by the aminocarbyne (iminium) substituents with respect to the terminal CO and L ligands. This kind of isomerism was previously recognized in many other diiron aminocarbyne complexes of general formula [Fe_2_Cp_2_(CO)(X)(µ-CO){µ-CN(Me)(Y)}]^0/+^ (X = anionic or neutral ligand ≠ CO; Y ≠ Me), and isomers were generally named α and β (Figure 2); in most cases, Y and X are bulkier than Me and CO, respectively, therefore the α form is expected to be favoured over the β one for steric reasons [42,45,53,54].

Based on cross-comparison with the NMR data from a library of compounds, (**2–12**)^+^ exhibit cis geometry of the Cp rings, and the E (α) isomer (Y and L placed on opposite sides) is prevalent in (**2**)^+^ and (**3**)^+^, and slightly prevalent in (**4**)^+^, (**5**)^+^, (**6**)^+^, (**8**)^+^ and (**9**)^+^. On the other hand, the Z (β) isomer is major in (**11**)^+^, and slightly prevalent in (**12**)^+^. Looking at the ratios reported in Table 1, it is presumable that additional factors, other than the steric hindrance of Y and X, somehow contribute to the relative amount of E and Z isomers in the solution. The NMR spectra of (**10**)^+^ consist of four sets of resonances, arising from E–Z isomerism and, in addition, conformational isomerism resulting in two possible frozen orientations for the aryl substituents (Cl and Me) with respect to the Fe–Fe axis [55].

The increase of the ^13^C chemical shift affecting the nitrile carbon upon coordination (Δδ, see Table 1) agrees with the tendency of the nitrile to donate charge to the metal (R = 4-C_6_H_4_NO_2_ < 4-C_6_H_4_F < Ph < 4-C_6_H_4_NMe_2_ < ^t^Bu) and is almost identical for E and Z pairs. Coherently, Δδ slightly increases upon replacing the cyclohexyl on the aminocarbyne with a more electron-withdrawing group in the acetonitrile adducts (**2**)^+^ (Y = Cy) and (**12**)^+^ (Y = 4-C_6_H_4_OMe); charge donation from the NCMe ligand to the iron was enhanced in (**12**)^+^ compared to (**2**)^+^. In this framework, it is not surprising that, for aromatic substituents (complexes **4**, **5**, **6** and **7**), δ(N≡C) correlates quite well with the Hammett parameter σ_p_.

The influence of the nitrile substituent on the ^13^C NMR resonances of the carbonyl ligands is not appreciable within the series of complexes **(2–7)CF_3_SO_3_** (δ for terminal and bridging CO ligands occur in the ranges 212.3–213.6 ppm and 265.2–266.7 ppm, respectively). Otherwise, the collected ^13^C data for the carbyne carbon permit a correlation with the electronic properties of the nitriles. In general, in diiron aminocarbyne complexes, the aminocarbyne centre resonates in the 305–390 ppm interval, and its chemical shift increases on increasing the [FeFe] to carbyne back-donation, which is enhanced by the electron withdrawing power of the N-substituents [56]. Here, this tendency is verifiable in the series of acetonitrile complexes (**2Z**)^+^, (**11Z**)^+^ and (**12Z**)^+^ [δ = 330.2 (Y = Cy), 333.4 (Y = allyl), 338.1 (Y = 4-C_6_H_4_OMe)]. Accordingly, within (**2–7**)^+^, the lowest value of carbyne chemical shift has been detected for (**6**)^+^ (R = 4-C_6_H_4_NO_2_, δ = 329.0–329.2 ppm), corresponding to the minimum nitrile-to-iron donated charge (see also C_TOT_ in Table 4), thus resulting in less back-donation to the aminocarbyne {CNMe(Cy)}. In summary, the strongly π-acceptor bridging aminocarbyne moiety, rather than the carbonyl ligands, is sensitive to the nitrile substituent R, and the chemical shift of the former is informative about the electronic behaviour of the latter.

### 2.4. X-ray Diffraction Studies

**Figure 3:**
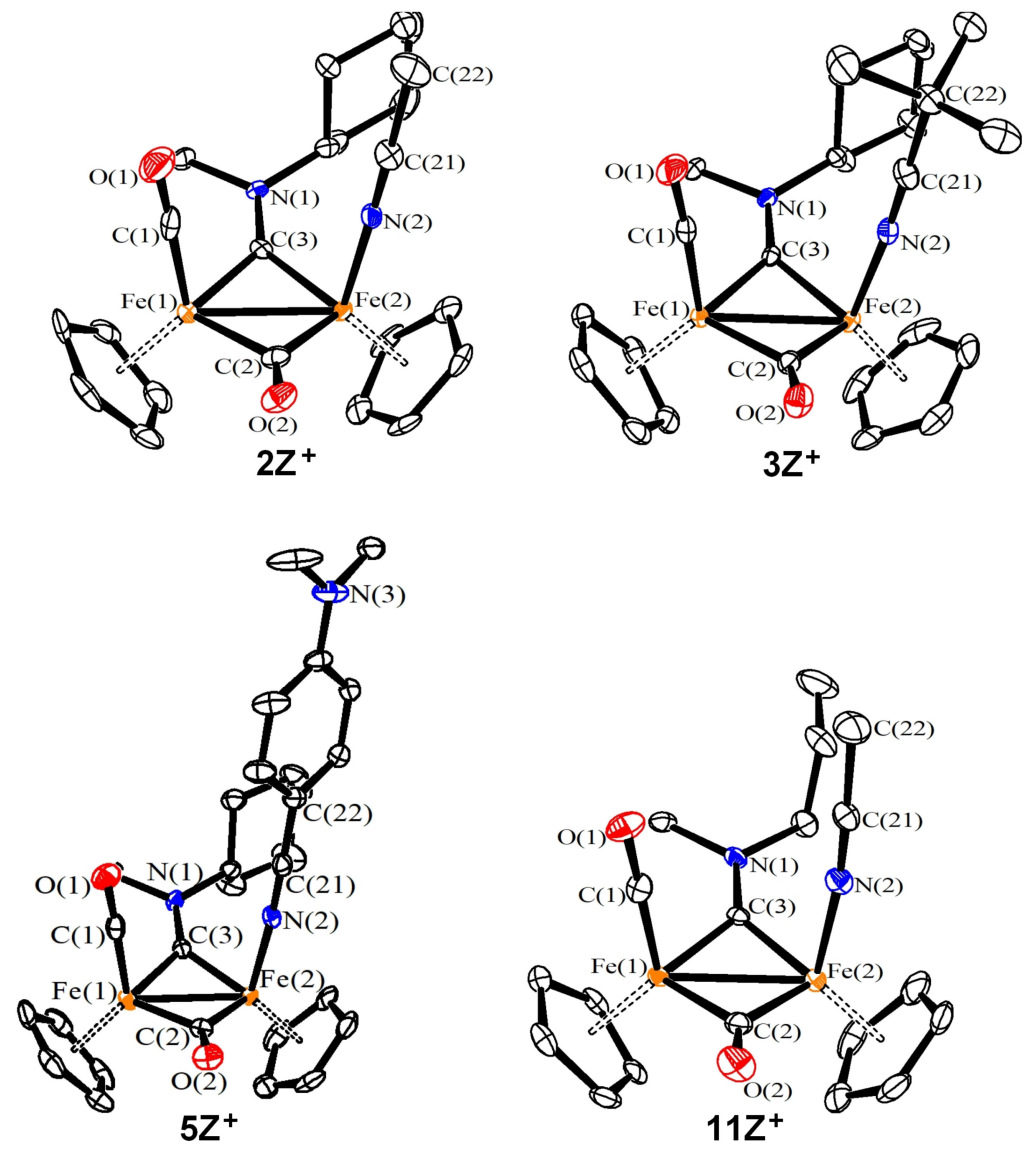
View of the molecular structures of the cations of **[2Z]CF_3_SO_3_**, **[3Z]CF_3_SO_3_**, **[5Z]CF_3_SO_3_** and **[11Z]CF_3_SO_3_**. Displacement ellipsoids are at the 30% probability level. H atoms have been omitted for clarity.

The molecular structures of **(2Z)CF_3_SO_3_**, **(3Z)CF_3_SO_3_**, **(5Z)CF_3_SO_3_** and **(11Z)CF_3_SO_3_** were determined by X-ray diffraction (Figure 3 and Table 2). The cations are based on a diiron core comprising the Cp ligands in *cis* position, bridging aminocarbyne and CO ligands, and terminal CO and nitrile ligands. The C(3)–N(1) interaction [1.288(7)–1.297(4) Å] displays some double-bond character, as typically found in analogous complexes [19,20,21,26,27,57,58], highlighting the hybrid aminocarbyne/iminium nature of the {µ-CN(Me)(Y)} ligand (Scheme 4). In all structures, the nitrile ligand is on the same side with respect to the bulkier aminocarbyne substituent Y (Z isomer). This configuration corresponds to the largely prevalent one, NMR-detected in the solution for (**11**)^+^, while the opposite is true for (**2**)^+^, (**3**)^+^ and (**5**)^+^ (E/Z ratio > 1 in solution, see above). The bridging CO shows a marked asymmetry, with the Fe(2)–C(2) interaction [1.887(6)–1.914(4) Å] considerably shorter than Fe(1)–C(2) [1.947(4)–1.969(6) Å]. This is in keeping with the fact that Fe(2) is bonded to a better σ-donor ligand (the nitrile) than Fe(1). The iron–nitrile distances are perceptibly different in (**3Z**)^+^ [1.928(3) Å] and (**5Z**)^+^ [1.912(3) Å], indicating that 4-dimethylaminobenzonitrile forms a stronger bond with iron than tert-butyl-nitrile ((**3Z**)^+^), a feature confirmed by DFT calculations (vide infra). Regarding the nitrile ligand, the N≡C distances and the N–C–C angles do not significantly differ in the four complexes.

Table 3 shows crystallographic data for a selection of iron–nitrile complexes from the literature. Regarding acetonitrile complexes, N≡C bond distances do not change significantly on varying the metal coordination environment, and are close to those found in **(2Z)CF_3_SO_3_** and **(11Z)CF_3_SO_3_**. Instead, the Fe–NCMe bond distances in a series of cyclopentadienyl monoiron complexes are finely sensitive to the ligand set, whereas the incidence of the net charge of the complex appears minor. For instance, the Fe–N length is shorter in [Fe_2_Cp_2_(CO)(NCMe)(µ-SMe)_2_]^2+^ [1.924(3) Å] than in [FeCp(CO)_2_(NCMe)]^+^ [1.935(4) Å], suggesting a higher degree of back-donation in the former complex, despite the higher total charge (+2 vs. +1), favoured by the substantial charge donation from the thiolato-groups. The Fe–NCMe distance reaches the maximum value in [Fe(NCMe)_6_]^2+^, i.e., 2.1510(12) Å.

In the iron(0)–pivalonitrile complex [Fe{=C(2-C_6_H_4_P^i^Pr_2_)_2_}(NC^t^Bu)(N_2_)], the Fe–N bond is considerably elongated—1.945(2) Å—compared to **(3Z)CF_3_SO_3_** [1.928(3) Å].

Interestingly, one other iron complex with the 4-dimethylaminobenzonitrile ligand was previously crystallographically characterized; i.e., the piano-stool [FeCp(Prophos){NC(4-C_6_H_4_NMe_2_)}]^+^ [16]. In this case, the Fe^II^-nitrile and N≡C bond lengths are consistent with the situation in **(5Z)CF_3_SO_3_**. The X-ray structure of the nitrile molecule is also available, outlining the invariance of the N≡C bond upon coordination [1.145(3) vs. 1.148(5) Å in **(5Z)CF_3_SO_3_]**.

In summary, the X-ray structures of homologous diiron aminocarbyne complexes and an overview of relevant literature data suggest that both the nitrile substituent and the coordination environment around the iron centre may influence iron–nitrile bonding. This feature is noticeable by looking at the Fe–N bond distance values, whereas the N≡C distance is almost unvaried in the different cases.

### 2.5. DFT Studies

Initially, we analysed as a model the diiron complex with the most simple nitrile and aminocarbyne substituents; i.e., [Fe_2_Cp_2_(CO)(µ-CO)(µ-CNMe_2_)(NCMe)]^+^, [**M1**]^+^ [20,67]. The DFT-computed increment of infrared wavenumber value for the N≡C bond, with respect to the isolated ligand, is Δῦ = +2.3 cm^−1^.

**Figure 4:**
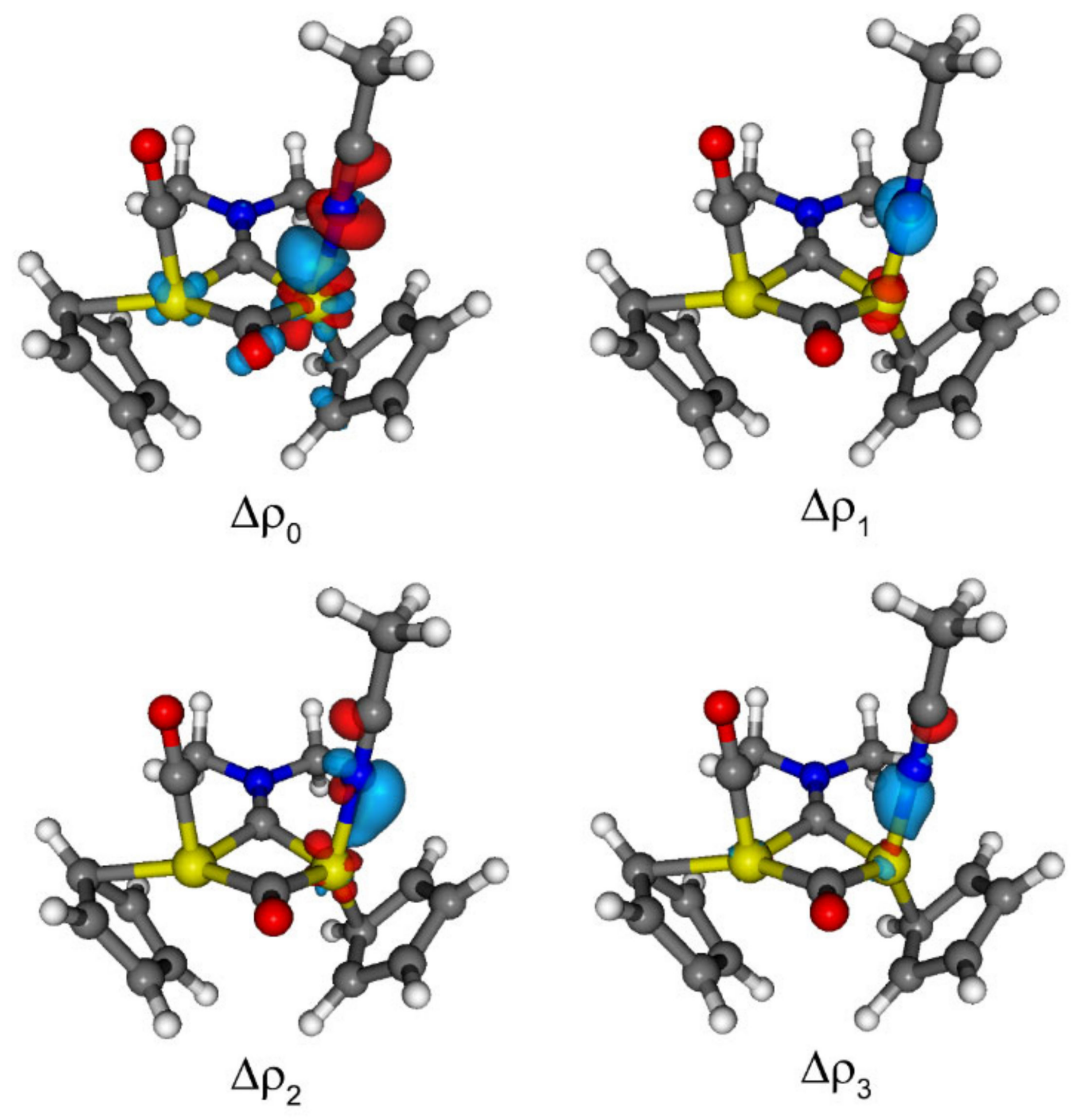
Isodensity surface plots (isodensity value 5 me a.u.^−3^ except for Δρ_3_, 2 me a.u.^−3^) for the deformation maps relative to Δρ_k_ of the [Fe_2_Cp_2_(CO)_2_(CNMe_2_)]^+^⋯NCMe interaction (complex (**M1**)^+^). The charge flux is red → blue.

To gain information about iron–nitrile bonding, [**M1**]^+^ was split into two fragments, i.e., [Fe_2_Cp_2_(CO)_2_(CNMe_2_)]^+^ and NCMe. According to energy decomposition analysis (EDA), the dissociation energy BDE is −46.1 kcal/mol, which is the sum of the interaction energy E_int_ (−48.9 kcal/mol) and the deformation energy (+2.8 kcal/mol). E_int_ can be further divided into steric (sum of the Pauli and the electrostatic term, E_st_, 19.3 kcal/mol), dispersion (E_disp_, −8.6 kcal/mol) and orbital (E_orb_, −59.6 kcal/mol) contributions. The latter is composed of four terms (ETS-NOCV analysis), namely Δρ_k_ (k = 0–3, Figure 4). Δρ_0_ is the most relevant one (ΔE_0_ = −33.2 kcal/mol) and, given the position and the local symmetry of the accumulation/depletion regions (blue/red coloured in Figure 4), it is associated with Fe ← N σ donation, accompanied by N ← C polarization of the N≡C bond (see Scheme 1). Notably, accumulation regions are present also on the other iron atom and on the bridging carbonyl ligand. Δρ_1_ is still relevant (ΔE_1_ = −11.1 kcal/mol) and can be associated with a Fe → N π back-donation; in this regard, the polarization of the triple bond is N → C. Δρ_2_ is slightly weaker than Δρ_1_ (ΔE_2_ = −9.6 kcal/mol) and is related to Fe → N π back-donation on a plane perpendicular to that for Δρ_1_. The sum of the two back-donation contributions (20.7 kcal/mol) represents 34.7% of the total orbital interaction. Finally, Δρ_3_ appears to be a simple N ← C polarization of the nitrile, likely due to the electrostatic attraction between the metal and the σ electrons on the ligand.

**Figure 5:**
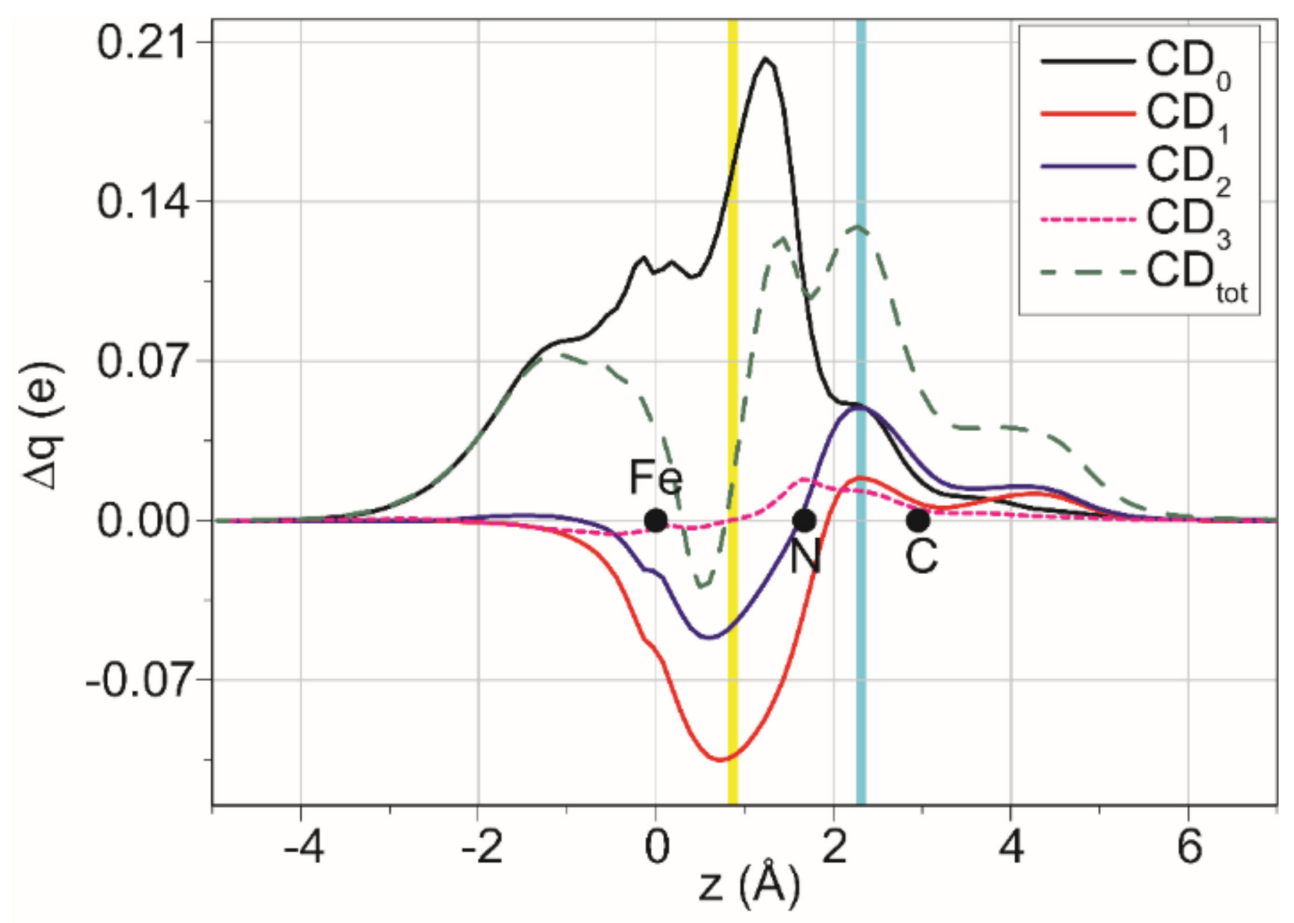
Charge Displacement function related to the [Fe_2_Cp_2_(CO)_2_(CNMe_2_)]^+^⋯NCMe interaction (complex (**M1**)^+^). Black dots indicate the position of the atomic nuclei on the axis. The yellow vertical band indicates the boundary between the fragments. The cyan vertical band indicates the half of the N≡C bond.

Contributions from k > 3 are negligible, with an associated ΔE_k_ < 0.6 kcal/mol. For a more detailed analysis, the different Δρ_k_ functions can be integrated along the Fe–N axis (charge displacement (CD) analysis), affording the CD_k_ functions shown in Figure 5. Each of them quantifies, at each point of the space, the number of electrons involved in the electronic rearrangement due to the Fe–N bond formation.

In CD_k_ functions, positive and negative values correspond to a charge flux from right to left and left to right, respectively. Indeed, the integration of Δρ_0_ leads to CD_0_, which is always positive and describes a displacement of electrons from the ligand to the metallic fragment (σ donation). Between the two fragments (isoboundary), such a displacement is equal to CT_0_ = 0.167 e, whereas at the middle of the triple bond it is CT_CN,0_ = 0.046 e, in accordance with the structures **A** and **B** in Scheme 1, and limited to σ electrons. Indeed, there is not one-to-one correspondence between CD_k_ curves and the resonance structures shown in Scheme 1, but each CD_k_ can be seen as a combination of more resonance structures.

The integration of Δρ_1_ and Δρ_2_ leads to CD_1_ and CD_2_, respectively, which display a more complex shape. At the isoboundary, CT_1_ and CT_2_ are −0.102 and −0.043 e, thus indicating a remarkable Fe → N back-donation. The total is CT^π^_tot_ = −0.145 e, which is only slightly smaller than CT_0_ (0.167 e). Note that both CD_1_ and CD_2_ are positive at the nitrile bond, since the N ← C polarization prevails (CT_CN,1_ = 0.018, CT_CN,2_ = 0.047 e). Being back-donation and polarization opposite in sign, there is a point where the sum of the two functions is null, generally around the z coordinate of the N atom. Therefore, despite the large back-donation, the N≡C bond is reinforced (Δῦ > 0), as the polarization of the π electrons due to structures **A** and **B** (Scheme 1) is more than that due to back-donation (structures **C** and **D**).

Note that, in terms of displaced electrons, the back-donation is almost equivalent to the donation, (CT_1_ + CT_2_)/CT_0_ = 0.86, whereas in terms of energy the ratio is lower, (ΔE_1_ + ΔE_2_)/ΔE_0_ = 0.62. This depends on the fact that the energy contributions are related to the whole molecule, including all the polarization regions that could be indirectly related to the iron–nitrile bond, whereas CD functions are calculated at a specific point of the molecule (in this case at the boundary between the two fragments [Fe_2_Cp_2_(CO)_2_(CNMe_2_)]^+^ and NCMe, see Computational Details).

Finally, Δρ_3_ can also be integrated, leading to CD_3_. The latter is negligible in the organometallic region, and slightly positive within the N≡C bond. As pointed out before, this is a small, additional polarization of the N≡C bond (0.012 e) upon coordination to the iron. The latter contribution is coherent with structure **A** in Scheme 1. The total polarization of the triple bond is CT_CN_ = 0.121 e. The sum of all components between the iron and the nitrogen is CT_tot_ = 0.034 e.

The polarization is remarkable even in the region of the methyl group (Δq = 0.036 e, at the carbon, and 0.017 e, at the hydrogen atoms). The electronic polarization of the methyl group is in alignment with the marked acidity manifested by the acetonitrile ligand in diiron aminocarbyne complexes,^21a^ as well as in other organometallic systems [68,69]. We extended the computational analysis to the terminal {Fe–CO} bond within (**M1**)^+^ (Figure S48) and we found that the interaction energy is much stronger for {Fe–CO} than {Fe–NCMe} (E_int_ = −65.7 kcal/mol vs. −45.4 kcal/mol). In addition, for {Fe–CO}, the orbital contribution of back-donation (E_1_ + E_2_ = −56.1 kcal/mol) is even larger than the orbital contribution of the donation (E_0_ = −49.2 kcal/mol), while the contrary occurs for {Fe–NCMe} (E_1_ + E_2_ = −20.7 kcal/mol, E_0_ = −33.2 kcal/mol).

The same framework as that described for the model adduct (**M1**)^+^ retains its validity in the nitrile complexes (**2–7**)^+^, with numerical differences depending on the nitrile (R) and aminocarbyne (Y), substituents which are detailed in Table 4. A view of the DFT-optimized structures of (**2–7**)^+^ is provided as Supplementary Materials (Figure S49).

As the crystal structure of [2Z]CF_3_SO_3_ is available (Figure 3), we applied the NOCV-CD analysis to both the experimental geometry without further optimization ((**2Z_exp_**)^+^) and the optimized geometry ((**2Z**)^+^), to evaluate the influence of crystal lattice on the Fe–N bond (Table 4). Indeed, there is a difference in the experimental and computed bond lengths, as the Fe–N and N–C distances measure 1.924(5) and 1.126(7) Å in the crystal structure and 1.865 and 1.163 Å in the optimized geometry, respectively. Such a difference is reflected on the bonds in (**2Z_exp_**)^+^ and (**2Z**)^+^: in the former, both σ and π contributions are smaller than in the latter, in absolute value, in terms of energy and electrons. This is likely due to the longer Fe–N distance in (**2Z_exp_**)^+^, as all the bonding contributions decrease as distance increases.

By comparing the bond splitting in (**2Z**)^+^ and in (**M1**)^+^, the contributions are strikingly similar, indicating that the presence on the aminocarbyne ligand of a cyclohexyl group ((**2Z**)^+^), rather than a methyl ((**M1**)^+^), does not substantially affect the iron–nitrile bonding. NOCV-CD analysis on the isomer (**2E**)^+^ led to almost identical values as (**2Z**)^+^, revealing that the relative geometry of the nitrile and the cyclohexyl is also not important. Accordingly, the calculated Δῦ does not change significantly (+2.3 cm^−1^ in (**M1**)^+^, +2.5 cm^−1^ in (**2Z**)^+^, +2.2 cm^−1^ in (**2E**)^+^).

A systematic NOCV-CD analysis was carried out on complexes (**2–7**)^+^ (E isomers). An acceptable correlation exists between experimental and computed Δῦ values (r^2^ = 0.85), although the former are systematically lower than the latter. As these complexes show quite close experimental Δῦ values (Table 1), additional theoretical systems [Fe_2_Cp_2_(CO)(µ-CO){µ-CNMe(Cy)}(NCR)]^+^ with fluorinated nitriles were included to widen the spectral window of the analysis (R = CF_3_, (**M2**)^+^; R = CHF_2_, (**M3**)^+^; R = CH_2_F, (**M4**)^+^; see Figure S50, Supplementary Materials).

The orbital energy associated with the back-donation ranges from 31.9% to 46.6% of the total bonding interaction. Looking at the CT values, back-donation roughly increases its relative weight on increasing the electron-withdrawing character of R, although clear deviations from this trend are noticeable; more precisely, the CT^π^_tot_/CT_0_ ratios vary from 0.75 ([5]^+^) to 2.01 ((**M2**)^+^), along the sequence (**5**)^+^ < (**3**)^+^ < (**2**)^+^ < (**7**)^+^ < (**4**)^+^ < (**6**)^+^ < (**M4**)^+^ < (**M3**)^+^ < (**M2**)^+^. On the other hand, the computed Fe–N bond energy (in absolute value) increases in the order (**M2**)^+^ < (**M3**)^+^ < (**M4**)^+^ < (**2**)^+^ < (**6**)^+^ < (**3**)^+^ = (**7**)^+^ < (**4**)^+^ < (**5**)^+^, pointing out that an appropriate balance of s-donation and p-back donation may be beneficial to the iron–nitrile bond stability. Interestingly, complex (**5**)^+^ (R = 4-C_6_H_4_NMe_2_) combines the lowest CT^π^_tot_/CT_0_ ratio with the highest BDE of the series: the special strength of the coordination of 4-dimethylaminobenzonitrile in (**5**)^+^ agrees with experimental findings on another iron system (see Introduction) [16].

Regarding the {N≡C} polarization, CT_CN_ is always positive (N ← C) and remarkable, ranging from 0.032 e ((**M2**)^+^) to 0.150 e ((**5E**)^+^). Considering only the π contribution (CT^π^_CN_), the polarization may become null when opposing electron fluxes counterbalance each other ((**M3**)^+^), or even negative (−0.051 e, (**M2**)^+^). As explained above for (**M1**)^+^, in general the N ← C polarization due to electrostatic and σ metal–nitrile interactions is not sufficiently balanced by back-donation from iron (Scheme 1), resulting in a positive value of Δῦ and favouring nucleophilic attack at the nitrile carbon (see Introduction) [46,47,48]. In the presence of significantly π-acidic substituents on the nitrile, the polarization may be inverted (N → C), leading to negative Δῦ values. Therefore, it has to be remarked that a positive value of Δῦ is not an index of absence of back-donation, in analogy to what previously demonstrated for carbonyl complexes,^41^ and advising caution in the interpretation of the metal–nitrile bonding based on infrared data only [1,10,12,70,71].

**Figure 6:**
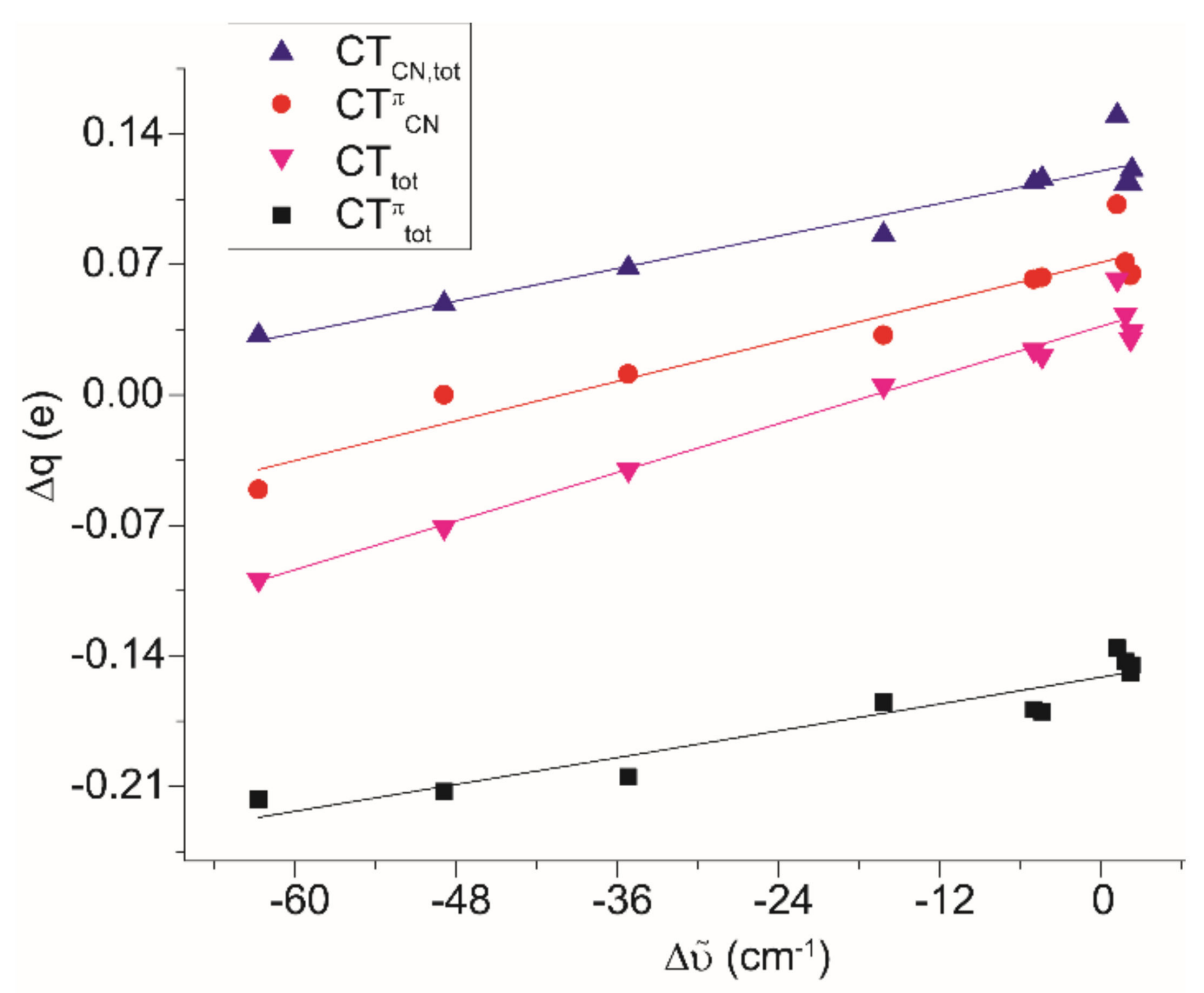
Correlation between bond components and IR frequency shifts (calculated Δῦ) for complexes **(M1–M4)^+^** and **(2–7)^+^** (see Table 4). Correlation factors are r^2^ = 0.89 for CT^π^_tot_, 0.90 for CT^π^_CN_, 0.89 for CT_CN,tot_ and 0.97 for CT_tot_.

The computed Δῦ values in Table 4 correlate well with the relevant bond contributions, the correlation factors varying from 0.89 (with CT^π^_tot_ and CT_CN,tot_) to 0.97 (with CT_tot_, see Figure 6). It can be concluded that the Fe → N≡CR back-donation is important and tunable, and appreciably influences the IR vibration of the N≡C bond. Similarly, it was proposed for carbonyl complexes that the experimental infrared stretching wavenumber is proportional to the degree of metal to CO π-back-donation and, more precisely, to the polarization of the π electrons on the carbon-oxygen bond [72,73,74].

## 3. Conclusions

Nitriles (N≡CR) have been largely employed in coordination chemistry and, despite being regarded in several cases as relatively weak ligands, the occurrence of metal to nitrile π-back-donation has been proposed. To support this view, experimental and theoretical proofs have been supplied with reference to diverse metal systems, but a comprehensive crystallographic, spectroscopic and computational approach is rare in the literature. Here, we have exploited an easily accessible di-organoiron scaffold to explore the bonding between one iron centre and a range of nitriles, using X-ray, IR, NMR and DFT methods.

Computational results outlined that the relative contribution of Fe → N π-back-donation is normally strong but only marginally dependent on the nature of the nitrile. A comparative view of X-ray structures, extended to additional literature iron compounds, highlighted that different nitrile substituents (R) may affect the Fe–N bond distance but not the N≡C one. More finely, the shifts of infrared stretching vibration (Δῦ) and ^13^C NMR resonance (Δδ) related to the nitrile function upon coordination to the metal rigorously correlate with the electronic properties of R. Besides, DFT studies clarify that a positive value of Δῦ is not evidence for a lack of back-donation, as sometimes misconceived in the literature; a parallelism emerges between metal–nitrile and metal–carbonyl bonds, in terms of the relationship between IR stretching vibration and ligand polarization, with the necessary distinctions in terms of donation/back-donation ratio. Note that IR and NMR data clearly demonstrate an interplay between the nitrile ligand and the strongly π-acceptor bridging aminocarbyne ligand. Overall, the established scales of Δῦ, Δδ and ^13^C NMR carbyne resonance values are indicators of the electronic behaviour of R holding a predictive potential in such regard. Nevertheless, the degree of back-donation is not strictly correlated with the electron withdrawing power of R; furthermore, increasing the back-donation does not seem a guarantee of strengthening the iron–nitrile bond. DFT outcomes, partially supported here by experimental X-ray analysis, indicate N≡C(4-C_6_H_4_NMe_2_) as a convenient choice for a nitrile ligand pointing to a relatively stable metal coordination.

## 4. Experimental

### 4.1. Materials and Methods

**Figure 7:**
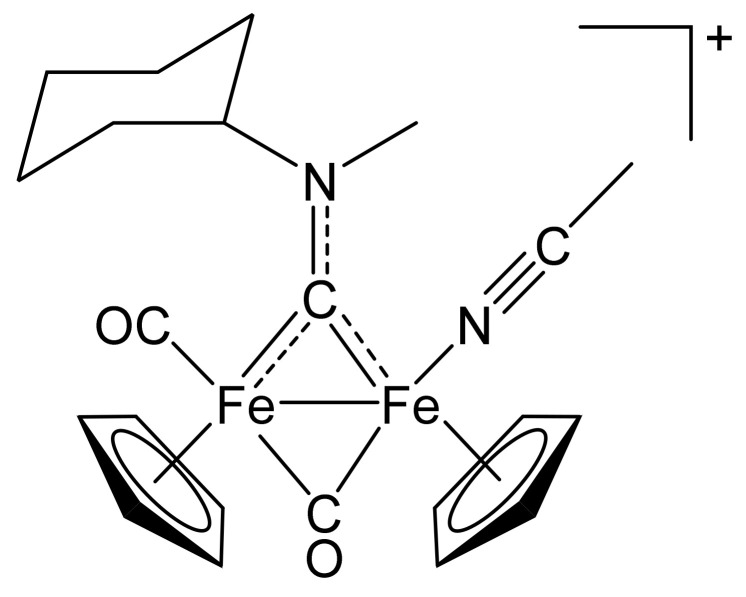
Structure of the cation of **(2)CF_3_SO_3_**.

**Figure 8:**
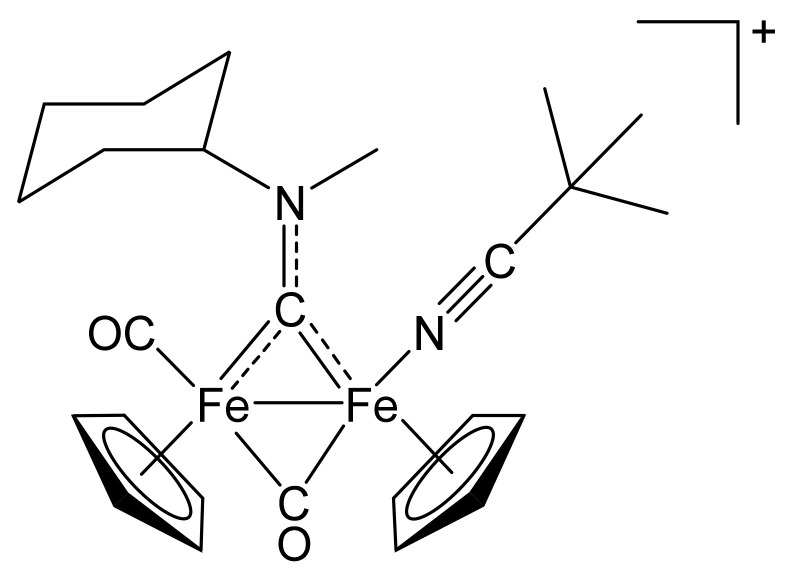
Structure of the cation of **(3)CF_3_SO_3_**.

**Figure 9:**
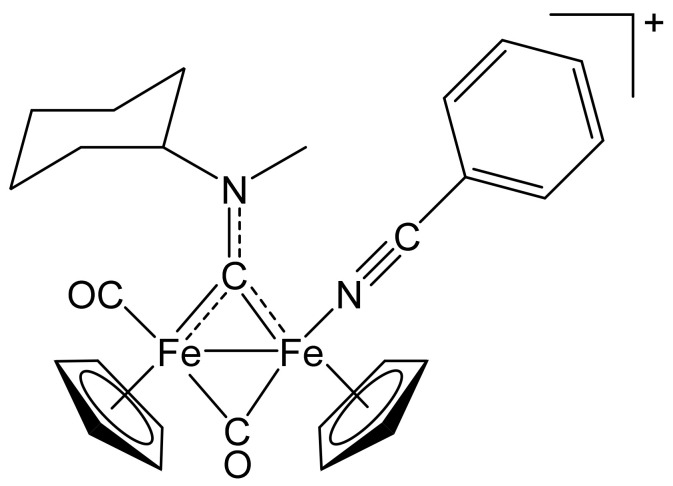
Structure of the cation of **(4)CF_3_SO_3_**.

**Figure 10:**
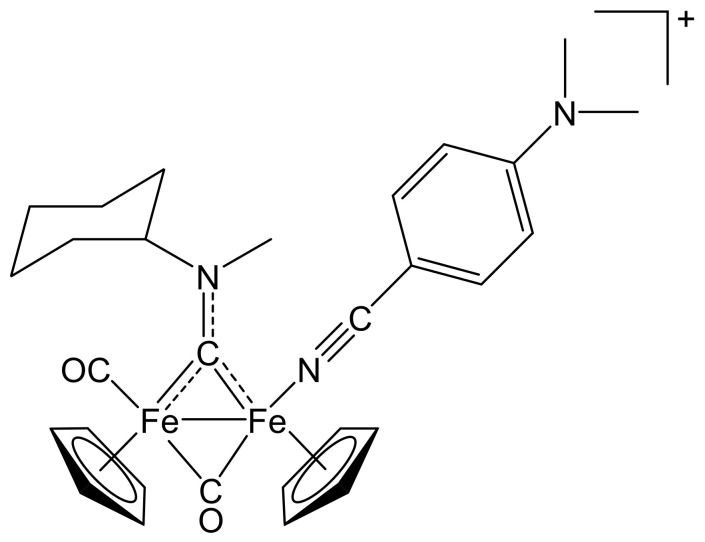
Structure of the cation of **(5)CF_3_SO_3_**.

**Figure 11:**
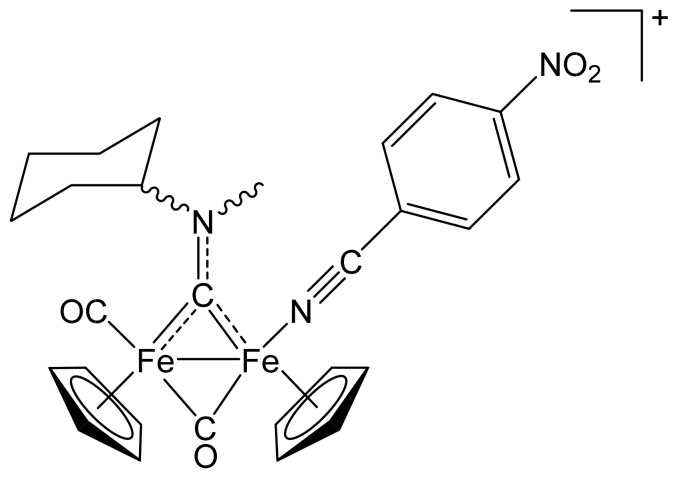
Structure of the cation of **(6)CF_3_SO_3_**.

**Figure 12:**
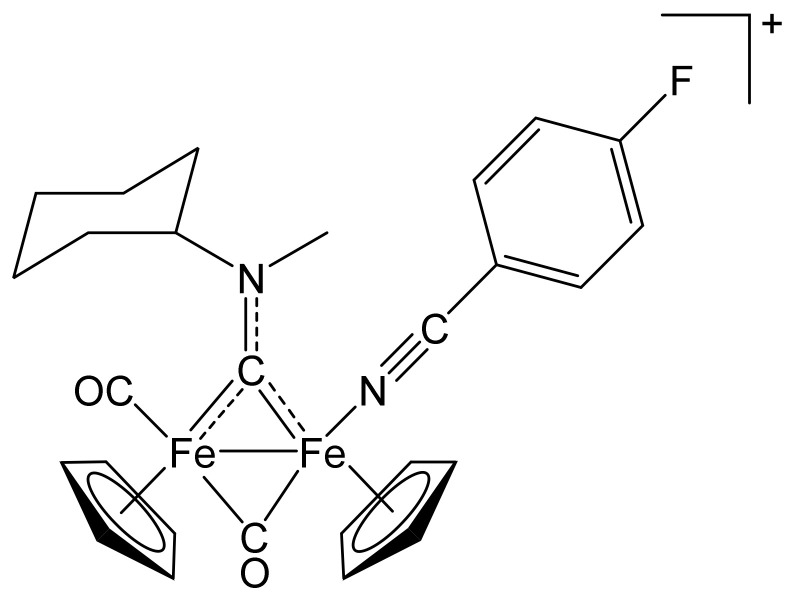
Structure of the cation of **(7)CF_3_SO_3_**.

**Figure 13:**
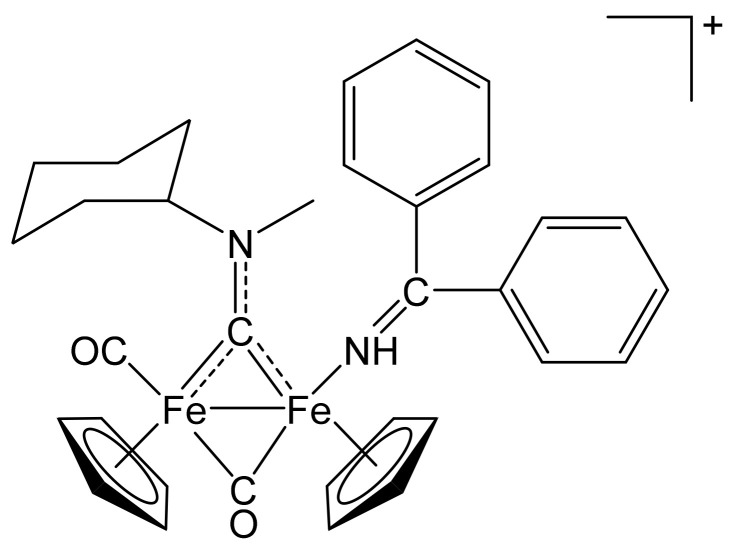
Structure of the cation of **(8)CF_3_SO_3_**.

**Figure 14:**
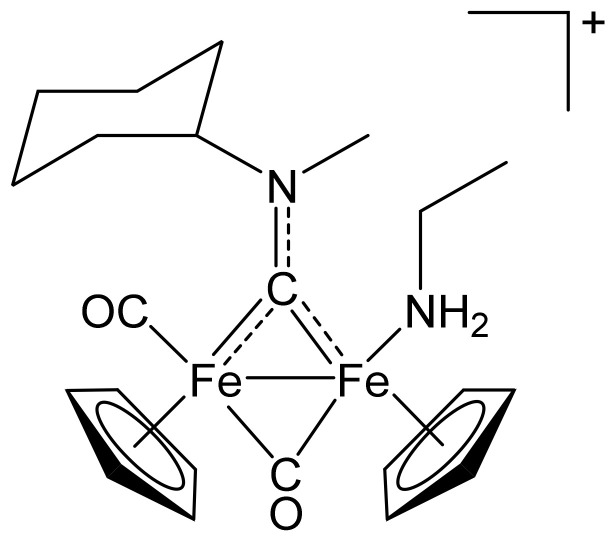
Structure of the cation of **(9)CF_3_SO_3_**.

**Figure 15:**
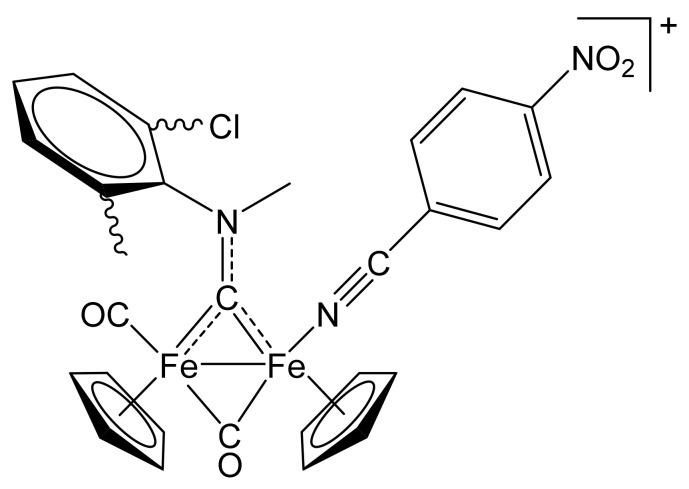
Structure of the cation of **(10)CF_3_SO_3_**.

**Figure 16:**
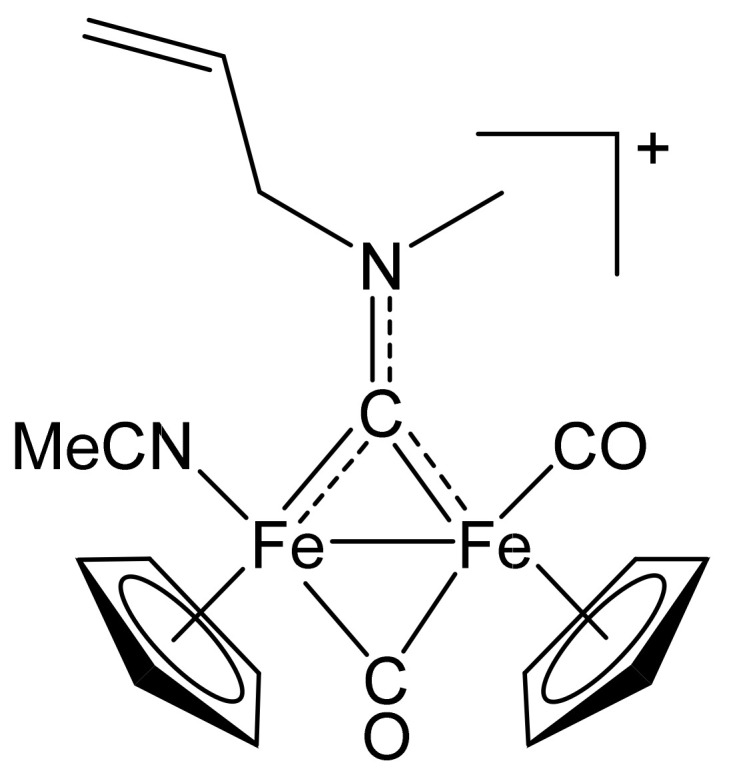
Structure of the cation of **(11)CF_3_SO_3_**.

**Figure 17:**
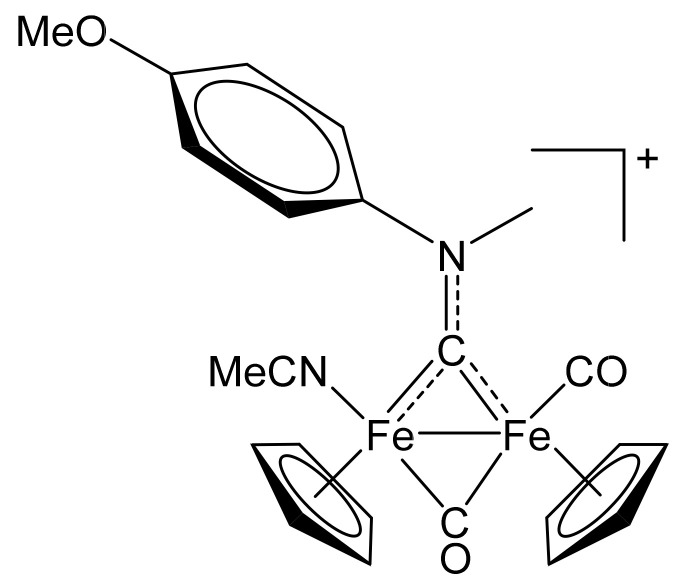
Structure of the cation of **(12)CF_3_SO_3_**.

Reactants and solvents were obtained from Alfa Aesar, Merck, Strem and TCI Chemicals and were of the highest purity available. Complexes **(1a,c,d)CF_3_SO_3_** [43] and **(1b)CF_3_SO_3_** [27] were prepared according to the literature. Once isolated, all the products were stored under N_2_, or under air, for limited periods of time (<3 days). Synthetic procedures were conducted under N_2_ atmosphere using standard Schlenk techniques. CH_2_Cl_2_ and THF were dried with the solvent purification system mBraun MB SPS5, while MeCN was distilled from CaH_2_. Chromatography separations were carried out on columns of deactivated alumina (Merck, 4% *w*/*w* water). IR spectra of solutions were recorded using a CaF_2_ liquid transmission cell (2300–1500 cm^−1^) on a Perkin Elmer Spectrum 100 FT-IR spectrometer. IR spectra of solid samples (650–4000 cm^−1^) and liquid nitriles (acetonitrile and trimethylacetonitrile [49]) were recorded on a Perkin Elmer Spectrum One FT-IR spectrometer, equipped with a UATR sampling accessory. IR spectra were processed with Spectragryph software [75]. NMR spectra were recorded at 298 K on a Bruker Avance II DRX400 instrument equipped with a BBFO broadband probe. Chemical shifts (expressed in parts per million) are referenced to the residual solvent peaks [76] or to external standard (^19^F, CFCl_3_). NMR spectra were assigned with the assistance of ^1^H-^13^C (*gs*-HSQC and *gs*-HMBC) correlation experiments [77]. NMR signals due to secondary isomeric forms (where it has been possible to detect them) are italicized. Figure 7, Figure 8, Figure 9, Figure 10, Figure 11, Figure 12, Figure 13, Figure 14, Figure 15, Figure 16 and Figure 17 show the prevalent isomer detected by NMR for each case. Elemental analyses were performed on a Vario MICRO cube instrument (Elementar).

### 4.2. Synthesis and Characterization of Compounds

#### 4.2.1. Synthesis and Characterization of [Fe_2_Cp_2_(CO)(NCMe)(µ-CO){µ-CN(Me)(Cy)}]CF_3_SO_3_, **(2)CF_3_SO_3_**

In a 25 mL Schleck tube, a mixture of **(1a)CF_3_SO_3_** (233 mg, 0.389 mmol), Me_3_NO∙2H_2_O (48 mg, 0.43 mmol) and MeCN (5 mL) was stirred for 2 h at room temperature. Afterwards, volatiles were evaporated under vacuum; the solid residue was dissolved in CH_2_Cl_2_ and the solution charged on an alumina column. Elution with THF allowed to separate impurities, then the fraction corresponding to the title compound was eluted using neat MeCN. Solvent was removed under reduced pressure and the residue was suspended in Et_2_O (50 mL) for 2 h. The brown powder was recovered by filtration and dried under vacuum. The yield was 180 mg (76%); soluble in MeCN, CH_2_Cl_2_, acetone; insoluble in Et_2_O; X-ray quality crystals of **(2)CF_3_SO_3_** were obtained from a MeCN solution layered with Et_2_O and settled aside at −20 °C. Anal. calcd. for C_23_H_27_F_3_Fe_2_N_2_O_5_S: C, 45.12; H, 4.44; N, 4.57; S, 5.24. Found: C, 45.21; H, 4.40; N, 4.50; S, 5.31. IR (solid): ῦ/cm^−1^ = 2279 vw (C≡N), 1966 vs (CO), 1807 s (µ-CO), 1543 m (µ-CN). IR (CH_2_Cl_2_): ῦ/cm^−1^ = 2277 w (C≡N), 1985 vs (CO), 1818 s (µ-CO), 1561 w (µ-CN), 1540 w. IR (MeCN): ῦ/cm^−1^ = 1980 s (CO), 1811 s (µ-CO), 1562 m (µ-CN), 1543 m. ^1^H NMR (CDCl_3_): δ/ppm = 5.78, 5.0 * (t, *J* = 12 Hz, 1 H, CH^Cy^); 5.20, 5.16, 5.01, 4.98 (s, 10 H, Cp); 4.53, 4.21 (s, 3 H, NMe); 2.86, 2.85 (s, 3 H, NC*Me*); 2.73, 2.58, 2.36–2.12, 2.02–1.35 (m, 10 H, CH_2_^Cy^). * Hidden by Cp resonances. ^13^C{^1^H} NMR (CDCl_3_): δ/ppm = 330.5 (μ-CN); 266.7 (μ-CO); 213.2, 212.3 (CO); 132.0, 131.8 (C≡N); 89.6, 89.4, 87.7, 87.6 (Cp); 78.4, 77.7 (CH^Cy^); 46.1, 45.3 (NMe); 32.7, 32.1, 31.6, 26.4, 26.3, 26.2, 25.9 (CH_2_^Cy^); 3.6 (NC*Me*). Isomer ratio (E/Z) = 65:35.

#### 4.2.2. General Procedure for the Synthesis of (3–10)CF_3_SO_3_

In a Schlenk tube, the starting complex (**(1a)CF_3_SO_3_** or **(1b)CF_3_SO_3_**) and Me_3_NO∙2H_2_O (1.1 eq.) were dissolved in THF (7 mL); then, the appropriate organic reactant (ca. 3.5 eq.) was added. The mixture was stirred for 2 h at room temperature, and then charged on an alumina column. Elution with CH_2_Cl_2_ and with CH_2_Cl_2_/THF mixture (2:1 *v*/*v*) allowed the separation of impurities, then the fraction corresponding to the product was collected using THF/MeOH mixture (10:1 *v*/*v*). Volatiles were evaporated under reduced pressure, and the residue was suspended in Et_2_O (15 mL) for 2 h. The obtained powder was recovered by filtration and dried under vacuum.

##### [Fe_2_Cp_2_(CO)(NCCMe_3_)(µ-CO){µ-CN(Me)(Cy)}]CF_3_SO_3_, **(3)CF_3_SO_3_**

From **(1a)CF_3_SO_3_** (100 mg, 0.167 mmol) and trimethylacetonitrile (65 µL, 0.58 mmol). Dark-brown solid, yield 75 mg (69%). X-ray quality crystals of **(3)CF_3_SO_3_** were obtained from a CH_2_Cl_2_ solution layered with pentane and settled aside at − 20°C. Anal. calcd. for C_26_H_33_F_3_Fe_2_N_2_O_5_S: C, 47.73; H, 5.08; N, 4.28; S, 4.90 Found: C, 47.75; H, 5.10; N, 4.19; S, 4.81. IR (solid state): ῦ/cm^−1^ = 2264 w (C≡N), 1967 vs. (CO), 1799 s (µ-CO), 1544 m (µ-CN). IR (CH_2_Cl_2_): ῦ/cm^−1^ = 2264 w (C≡N), 1984 vs (CO), 1820 s (µ-CO), 1559 w (µ-CN). ^1^H NMR (acetone-d_6_): δ/ppm = 5.79, 5.0 * (m, 1 H, CH^Cy^); 5.22, 5.18, 5.03, 4.98 (s, 10 H, Cp); 4.54, 4.22 (s, 3 H, NMe); 2.74, 2.59, 2.35–2.14, 1.93–1.56, 1.40–1.22 (m, 10 H, CH_2_^Cy^); 1.08, 1.06 (s, 9 H, CMe_3_). * Hidden by Cp resonances. ^13^C{^1^H} NMR (acetone-d_6_): δ/ppm = 330.7, 330.1 (μ-CN); 266.7, 266.1 (μ-CO); 213.6, 212.6 (CO); 140.0, 139.8 (C≡N); >89.8, 89.6, 88.1, 87.9 (Cp); 78.4, 77.9 (CH^Cy^); 46.3, 45.3 (NMe); 35.1, 33.1, 32.1, 31.7, 31.4, 30.8, 26.4, 26.3, 26.0, 25.9 (CH_2_^Cy^); 27.7 (*C*Me_3_); 27.6 (C*Me*_3_). Isomer ratio (E/Z) = 70:30. 

##### [Fe_2_Cp_2_(CO)(NCPh)(µ-CO){µ-CN(Me)(Cy)}]CF_3_SO_3_, **(4)CF_3_SO_3_**

From **(1a)CF_3_SO_3_** (100 mg, 0.167 mmol) and benzonitrile (60 µL, 0.58 mmol). Pale-brown solid, yield 93 mg (80%). Anal. calcd. for C_28_H_29_F_3_Fe_2_N_2_O_5_S: C, 49.88; H, 4.34; N, 4.15; S, 4.75. Found: C, 49.79; H, 4.38; N, 4.19; S, 4.83. IR (solid): ῦ/cm^−1^ = 2238 w (C≡N), 1965 vs (CO), 1799 vs (µ-CO), 1540 m (µ-CN). IR (CH_2_Cl_2_): ῦ/cm^−1^ = 2238 w (C≡N), 1984 vs (CO), 1820 s (µ-CO), 1560 w (µ-CN). ^1^H NMR (acetone-d_6_): δ/ppm = 7.66–7.40 (m, 5 H, Ph); 5.84, 5.0 * (t, *J* = 11.6 Hz, 1 H, CH^Cy^); 5.28, 5.24, 5.17, 5.13 (s, 10 H, Cp); 4.59, 4.23 (s, 3 H, NMe); 2.76, 2.61, 2.35–2.22, 1.91–1.29 (m, 10 H, CH_2_^Cy^). * Hidden by Cp resonances. ^13^C{^1^H} NMR (acetone-d_6_): δ/ppm = 330.0, 329.7 (μ-CN); 266.0, 265.8 (μ-CO); 213.3, 212.4 (CO); 134.9, 134.8, 133.2, 133.1, 130.5, 130.4, 111.4, 111.3 (Ph); 131.6, 131.2 (C≡N); 89.9, 89.8, 88.4, 88.3 (Cp); 78.6, 78.1 (CH^Cy^); 46.3, 45.5 (NMe); 33.0, 32.0, 31.7, 31.5, 30.8, 26.4, 26.3, 26.2, 25.9 (CH_2_^Cy^). Isomer ratio (E/Z) = 54:46.

##### [Fe_2_Cp_2_(CO){NC(4-C_6_H_4_NMe_2_)}(µ-CO){µ-CN(Me)(Cy)}]CF_3_SO_3_, **(5)CF_3_SO_3_**

From **(1a)CF_3_SO_3_** (100 mg, 0.167 mmol) and 4-dimethylaminobenzonitrile (85 mg, 0.58 mmol). Brown solid, yield 100 mg (83%). X-ray quality crystals of **(5)CF_3_SO_3_** were obtained from a CH_2_Cl_2_ solution layered with pentane and settled aside at − 20 °C. Anal. calcd. for C_30_H_34_F_3_Fe_2_N_3_O_5_S: C, 50.23; H, 4.78; N, 5.86; S, 4.47. Found: C, 50.29; H, 4.75; N, 5.07; S, 4.37. IR (solid state): ῦ/cm^−1^ = 2227 w (C≡N), 1964 vs (CO), 1798 s (µ-CO), 1529 m (µ-CN). IR (CH_2_Cl_2_): ῦ/cm^−1^ = 2227 vw (C≡N), 1985 vs (CO), 1819 s (µ-CO), 1561 w (µ-CN). ^1^H NMR (acetone-d_6_): δ/ppm = 7.13, 6.97, 6.63 (m, 4 H, C_6_H_4_); 5.84, 5.0 * (m, 1 H, CH^Cy^); 5.24, 5.20, 5.10, 5.06 (s, 10 H, Cp); 4.58, 4.21 (s, 3 H, NMe); 3.00, 2.86 (s, 6 H, NMe_2_); 2.77, 2.62, 2.34–2.22, 1.89–1.36 (m, 10 H, CH_2_^Cy^). * Hidden by Cp resonances. ^13^C{^1^H} NMR (acetone-d_6_): δ/ppm = 330.9, 330.4 (μ-CN); 266.6, 266.3 (μ-CO); 213.4, 212.6 (CO); 154.2, 134.9, 134.3, 112.4, 112.3, 95.4, 95.3 (C_6_H_4_); 133.9, 133.4, (C≡N); 89.7, 89.6, 88.1, 88.0 (Cp); 78.4, 77.9 (CH^Cy^); 46.1, 45.3 (NMe); 39.9 (NMe_2_); 32.9, 32.1, 31.7, 31.5, 30.8, 26.4, 26.3, 26.1, 25.9 (CH_2_^Cy^). Isomer ratio (E/Z) = 53:47. 

##### [Fe_2_Cp_2_(CO){NC(4-C_6_H_4_NO_2_)}(µ-CO){µ-CN(Me)(Cy)}]CF_3_SO_3_, **(6)CF_3_SO_3_**

From **(1a)CF_3_SO_3_** (100 mg, 0.167 mmol) and 4-nitrobenzonitrile (87 mg, 0.58 mmol). Dark-red solid, yield 85 mg (71%). Anal. calcd. for C_28_H_28_F_3_Fe_2_N_3_O_7_S: C, 46.76; H, 3.92; N, 5.84; S, 4.46. Found: C, 46.62; H, 3.98; N, 5.79; S, 4.53. IR (solid state): ῦ/cm^−1^ = 2230 w (C≡N), 1966 vs (CO), 1801 s (µ-CO), 1526 m (µ-CN). IR (CH_2_Cl_2_): ῦ/cm^−1^ = 2230 vw (C≡N), 1982 vs (CO), 1821 s (µ-CO), 1561 s (µ-CN). ^1^H NMR (acetone-d_6_) *cis*-isomers: δ/ppm = 8.31,7.74 (m, 4 H, C_6_H_4_); 5.82, 5.0 * (m, 1 H, CH^Cy^); 5.33, 5.29, 5.23, 5.19 (s, 10 H, Cp); 4.60, 4.26 (s, 3 H, NMe); 2.78, 2.61, 2.35–1.42 (m, 10 H, CH_2_^Cy^). * Hidden by Cp resonances. ^1^H NMR (acetone-d_6_) *trans*-isomers: δ/ppm = 8.48, 8.15 (m, 4 H, C_6_H_4_); 4.92, 4.89, 4.69, 4.66 (s, 10 H, Cp); 4.55, 4.14 (s, 3 H, NMe). ^13^C{^1^H} NMR (acetone-d_6_): δ/ppm = 329.2, 329.0 (μ-CN); 265.4, 265.2 (μ-CO); 213.1, 212.3 (CO); 151.1, 134.9, 134.8, 125.3, 125.2, 117.1, 116.9(C_6_H_4_); 129.6, 129.2 (C≡N); 90.1, 89.9, 88.8, 88.6 (Cp); 78.7, 78.2 (CH^Cy^); 46.4, 45.6 (NMe); 35.0, 33.1, 32.0, 31.6, 31.5, 30.8, 26.3, 26.0, 25.8 (CH_2_^Cy^). ^13^C{^1^H} NMR (acetone-d_6_) *trans*-isomers: δ/ppm = 89.6, 89.4, 87.9, 87.8 (Cp). Isomer ratio (cis-E/cis-Z) = 56:44. Isomer ratio (trans-E/trans-Z) = 50:50. Isomer ratio (cis/trans) = 92:8.

##### [Fe_2_Cp_2_(CO){NC(4-C_6_H_4_F)}(µ-CO){µ-CN(Me)(Cy)}]CF_3_SO_3_, **(7)CF_3_SO_3_**

From **(1a)CF_3_SO_3_** (100 mg, 0.167 mmol) and 4-fluorobenzonitrile (71 µL, 0.58 mmol). Brown solid, yield 81 mg (70%). Anal. calcd. for C_28_H_28_F_4_Fe_2_N_2_O_5_S: C, 48.58; H, 4.08; N, 4.05; S, 4.63. Found: C, 48.38; H, 4.12; N, 4.07; S, 4.67. IR (solid state): ῦ/cm^−1^ = 2240 w (C≡N), 1967 vs (CO), 1800 s (µ-CO), 1540 m (µ-CN). IR (CH_2_Cl_2_): ῦ/cm^−1^ = 2240 w (C≡N), 1983 vs (CO), 1819 s (µ-CO), 1559 m (µCN). ^1^H NMR (acetone-d_6_): δ/ppm = 7.52, 7.29 (m, 4 H, C_6_H_4_); 5.83, 5.0 * (m, 1 H, CH^Cy^); 5.28, 5.24, 5.16, 5.12 (s, 10 H, Cp); 4.59, 4.24 (s, 3 H, NMe); 2.73, 2.61, 2.31–2.20, 1.88–1.36 (m, 10 H, CH_2_^Cy^). * Hidden by Cp resonances. ^13^C{^1^H} NMR (acetone-d_6_): δ/ppm = 329.9, 329.7 (μ-CN); 265.9, 265.7 (μ-CO); 212.4, 212.3 (CO); 166.2, 136.4, 136.2, 118.4, 117.9, 107.9, 107.8 (d, C_6_H_4_); 130.7, 130.4 (C≡N); 89.9, 89.7, 88.4, 88.3 (Cp); 78.6, 78.1 (CH^Cy^); 46.3, 45.5 (NMe); 33.0, 32.0, 31.6, 31.5 26.3, 26.2, 26.1, 25.8 (CH_2_^Cy^). ^19^F{^1^H} NMR (acetone-d_6_): −102.0, −102.2 (s). Isomer ratio (E/Z) = 50:50.

##### [Fe_2_Cp_2_(CO)(NH=CPh_2_)(µ-CO){µ-CN(Me)(Cy)}]CF_3_SO_3_, **(8)CF_3_SO_3_**

From **(1a)CF_3_SO_3_** (100 mg, 0.167 mmol) and benzophenone imine (97 µL, 0.58 mmol). Dark-red solid, yield 78 mg (62%). Anal. calcd. for C_34_H_35_F_3_Fe_2_N_2_O_5_S: C, 54.28; H, 4.69; N, 3.72; S, 4.26. Found: C, 54.19; H, 4.58; N, 3.69; S, 4.18. IR (solid state): ῦ/cm^−1^ = 1955 vs. (CO), 1792 s (µ-CO), 1537 m (µ-CN), 1532 w (C=N). IR (CH_2_Cl_2_): ῦ/cm^−1^ = 1974 vs (CO), 1810 s (µ-CO), 1558 vw (µ-CN), 1531 w (C=NH). ^1^H NMR (acetone-d_6_): δ/ppm = 7.75–7.35, 6.95–6.81 (m, 10 H, Ph); 6.60, 6.38 (s, 1H, NH); 6.09, 4.8 * (m, 1 H, CH^Cy^); 5.16, 4.84, 4.78 (s, 10 H, Cp); 4.74, 4.42 (s, 3 H, NMe); 2.68, 2.50–2.30, 1.89–1.41 (m, 10 H, CH_2_^Cy^). * Hidden by Cp resonances. ^13^C{^1^H} NMR (acetone-d_6_): δ/ppm = 334.5 (μ-CN); 266.8 (μ-CO); 214.5 (CO); 192.7 (C=NH); 139.8, 137.2, 132.9, 132.8, 131.9, 131.8, 130.4, 130.2, 129.8, 129.7129.4, 129.1, 127.7, 126.1 (Ph); 90.3, 90.0, 88.0, 87.7 (Cp); 79.1, 76.6 (CH^Cy^); 47.6, 45.7 (NMe); 33.6, 33.2, 31.8, 31.7, 30.8, 26.4, 26.3, 25.9 (CH_2_^Cy^). Isomer ratio (E/Z) = 62:38.

##### [Fe_2_Cp_2_(CO)(NH_2_Et)(µ-CO){µ-CN(Me)(Cy)}]CF_3_SO_3_, (**9)CF_3_SO_3_**

From **(1a)CF_3_SO_3_** (100 mg, 0.167 mmol) and ethylamine (large excess of gas bubbled into the solution). Brown solid, yield 60 mg (58%). Anal. calcd. for C_23_H_31_F_3_Fe_2_N_2_O_5_S: C, 44.83; H, 5.07; N, 4.55; S, 5.20. Found: C, 44.93; H, 5.02; N, 4.62; S, 5.24. IR (solid state): ῦ/cm^−1^ = 1944 vs (CO), 1780 s (µ-CO). IR (CH_2_Cl_2_): ῦ/cm^−1^ = 1965 vs (CO), 1800 s (µ-CO), 1560 vw (µ-CN). ^1^H NMR (acetone-d_6_): δ/ppm = 5.85, 5.0 * (m, 1 H, CH^Cy^); 5.11, 5.10, 5.01, 5.00 (s, 10 H, Cp); 4.54, 4.21 (s, 3 H, NMe); 3.06–2.89 (m, 2 H, CH_2_^amine^); 2.63, 2.54, 2.35–2.21, 1.97–1.40 (m, 10 H, CH_2_^Cy^); 0.73 (m, 3 H, CH_3_^amine^); −1.80, −1.89 (s, NH_2_). * Hidden by Cp resonances. ^13^C{^1^H} NMR (acetone-d_6_): δ/ppm = 331.9, 331.7 (μ-CN); 270.4, 269.6 (μ-CO); 215.2, 214.7 (CO); 89.7, 89.5, 87.8, 87.5 (Cp); 78.6, 75.7 (CH^Cy^); 47.0, 45.3 (NMe); 44.0, 43.9 (CH_2_^amine^); 35.1, 33.1, 32.7, 31.8, 30.8, 26.4, 26.3, 26.1, 26.0, 25.7 (CH_2_^Cy^); 17.4, 17.3 (CH_3_^amine^). Isomer ratio (E/Z) = 56:44.

##### [Fe_2_Cp_2_(CO){NC(4-C_6_H_4_NO_2_)}(µ-CO){µ-CN(Me)(2,6-C_6_H_3_MeCl)}]CF_3_SO_3_, **(10)CF_3_SO_3_**

From **(1b)CF_3_SO_3_** (100 mg, 0.156 mmol) and 4-nitrobenzonitrile (81 mg, 0.55 mmol). Red solid, yield 76 mg (64%). Anal. calcd. for C_29_H_23_ClF_3_Fe_2_N_3_O_7_S: C, 45.73; H, 3.04; N, 5.52; S, 4.21. Found: C, 45.67; H, 2.98; N, 5.49; S, 4.15. IR (solid state): ῦ/cm^−1^ = 2235 w (C≡N), 1975 vs (CO), 1810 s (µ-CO), 1514 (µ-CN). IR (CH_2_Cl_2_): ῦ/cm^−1^ = 1988 vs (CO), 1825 s (µ-CO), 1560 s (µ-CN). ^1^H NMR (acetone-d_6_): δ/ppm = 8.48–8.15, 7.86–7.61 (m, 7 H, arom CH); 5.49, 5.40, 5.00, 4.99, 4.87, 4.84, 4.75, 4.72 (s, 10 H, Cp); 3.64, 3.42 (m, 3 H, NMe); 2.44, 2.33, 2.29, 2.23 (s, 3 H, C_6_H_3_*Me*). ^13^C{^1^H} NMR (acetone-d_6_): δ/ppm = 342.2, 341.1, 341.8, 341.0 (μ-CN); 263.9, 263.7, 263.3, 262.7 (μ-CO); 212.9, 212.4, 212,1, 121.0 (CO); 152.6, 151.2, 147.2, 147.1, 146.9, 146.6, 138.3, 138.2, 138.0, 137.6, 136.5, 135.1, 135.0, 134.9, 134.8, 132.5, 131.4, 126.0, 125.3, 117.1, 117.0 (C_6_H_4_ + C_6_H_3_); 130.0, 129.9, 129.2, 129.1 (C≡N); 90.6, 89.7, 89.5, 89.4, 88.5, 88.4 (Cp); 55.7, 55.5, 54.6, 54.3 (NMe); 19.3, 19.1, 18.4, 18.1 (C_6_H_3_*Me*). Isomer ratio = 70 (cis-E, two conformers): 30 (cis-Z, two conformers).

#### 4.2.3. Synthesis and Characterization of [Fe_2_Cp_2_(CO)(NCMe)(µ-CO){µ-CN(Me)(CH_2_CH=CH_2_)}]CF_3_SO_3_, (11)CF_3_SO_3_

The title compound was prepared by a procedure analogous to that described for the synthesis of **(2)CF_3_SO_3_**, from **(1c)CF_3_SO_3_** (240 mg, 0.431 mmol) and Me_3_NO∙2H_2_O (53 mg, 0.47 mmol) in acetonitrile (7 mL). Brown solid, yield 182 mg (77%). X-ray quality crystals of **(11)CF_3_SO_3_** were obtained from a MeCN solution layered with Et_2_O and settled aside at − 20 °C. Anal. calcd. for C_20_H_21_F_3_Fe_2_N_2_O_5_S: C, 42.13; H, 3.71; N, 4.91; S, 5.62. Found: C, 42.18; H, 3.65; N, 4.88; S, 5.68. IR (solid): ῦ/cm^−1^ = 2277 vw (C≡N), 1956 vs (CO), 1803 s (µ-CO), 1571 (µ-CN). IR (CH_2_Cl_2_): ῦ/cm^−1^ = 1987 vs (CO), 1818 s (µ-CO), 1568 w (µ-CO). ^1^H NMR (acetone-d_6_): δ/ppm = 6.42 (m, 1 H, CH=); 5.86, 5.69 (dd, 2 H, =CH_2_); 5.55 (m, 2 H, NCH_2_); 5.23, 5.19, 5.04, 4.99 (s, 10 H, Cp); 4.63, 4.30 (s, 3 H, NMe); 2.07 (s, 3 H, NCMe).^13^C{^1^H} NMR (acetone-d_6_): δ/ppm = 333.4 (μ-CN); 266.3 (μ-CO); 212.3 (CO); 134.0, 133.2 (CH=); 132.1 (C≡N); 119.9, 119.5 (=CH_2_); 89.5, 89.4, 87.7, 87.5 (Cp); 70.2, 69.6 (NCH_2_); 51.3 (NMe); 3.7 (NC*Me*). Isomer ratio (Z/E) = 87:13. 

#### 4.2.4. Synthesis and Characterization of [Fe_2_Cp_2_(CO)(NCMe)(µ-CO){µ-CN(Me)(4-C_6_H_4_OMe)}]CF_3_SO_3_, (12)CF_3_SO_3_

The title compound was prepared by a procedure analogous to that described for the synthesis of **(2)CF_3_SO_3_**, from **(1d)CF_3_SO_3_** (262 mg, 0.420 mmol) and Me_3_NO∙2H_2_O (51 mg, 0.46 mmol) in acetonitrile (7 mL). Brown solid, yield 200 mg (75%). Anal. calcd. for C_24_H_23_F_3_Fe_2_N_2_O_6_S: C, 45.31; H, 3.64; N, 4.40; S, 5.04. Found: C, 45.38; H, 3.69; N, 4.32; S, 5.09. IR (solid): ῦ/cm^−1^ = 2281 vw (C≡N), 1972 vs (CO), 1802 s (µ-CO), 1505 (µCN). IR (CH_2_Cl_2_): ῦ/cm^−1^ = 1984 vs (CO), 1817 s (µ-CO), 1525 w (µ-CN). ^1^H NMR (acetone-d_6_): δ/ppm = 7.88–7.78, 7.27 (m, 4 H, C_6_H_4_); 5.32, 5.14, 4.67, 4.46 (s, 10 H, Cp); 5.01, 4.71 (s, 3 H, NMe); 3.97 (s, 3 H, OMe); 2.19, 2.15 (s, 3 H, NCMe).^13^C{^1^H} NMR (acetone-d_6_): δ/ppm = 338.2, 338.1 (μ-CN); 268.7, 266.0 (μ-CO); 213.3, 212.4 (CO); 160.6, 160.5, 145.4, 145.0, 127.9, 127.6, 115.9 (C_6_H_4_); 132.3, 132.1 (C≡N); 89.9, 89.5, 88.1, 87.6 (Cp); 57.3, 57.2 (NMe); 56.3, 56.2 (OMe); 3.9, 3.8 (NC*Me*). Isomer ratio (Z/E) = 55:45.

### 4.3. X-ray Crystallography

Crystal data and collection details for **(2)CF_3_SO_3_**, **(3)CF_3_SO_3_**, **(5)CF_3_SO_3_** and **(11)CF_3_SO_3_** are reported in Table 5. Data were recorded on a Bruker APEX II diffractometer equipped with a PHOTON2 detector using Mo–Kα radiation. The structures were solved by direct methods and refined by full-matrix least-squares based on all data using *F*^2^ [78]. Hydrogen atoms were fixed at calculated positions and refined using a riding model. All non-hydrogen atoms were refined with anisotropic displacement parameters. The crystals of **(2)CF_3_SO_3_** appeared to be non-merohedrally twinned. The TwinRotMat routine of PLATON [79] was used to determine the twinning matrix and to write the reflection data file (.hkl) containing the twin components. Refinement was performed using the instruction HKLF 5 in SHELXL and one BASF parameter, which refined as 0.262(3).

### 4.4. DFT Calculations

All geometries were optimized with ORCA 4.0.1.2 [80], using the BP86 functional in conjunction with a triple-ζ quality basis set (def2-TZVP). The dispersion corrections were introduced using the Grimme D3-parametrized correction and the Becke Johnson damping to the DFT energy [81]. Relativistic effects were treated with the scalar zeroth-order regular approximation (ZORA) [82,83], in conjunction with SARC/J auxiliary basis sets. Most of the structures were confirmed to be local energy minima (no imaginary frequencies), but in some cases a small, unavoidable negative frequency relative to the Cp rotation around the M-Cp axis was observed.

***Energy Decomposition Analysis*** [84]. The EDA has been performed using ORCA 4.1.0. The EDA allows the decomposition of the bond energy into physically meaningful contributions. The interaction energy (E_int_) is the difference of the energy between the adduct and the unrelaxed fragments. It can be divided into contributions associated with the orbital, steric, and dispersion interactions, as shown in Equation (1).

E_int_ = E_st_ + E_orb_ + E_disp_ (1)

E_st_ is usually called the steric interaction energy and it is the sum of E_elst_, the classical electrostatic interaction between the unperturbed charge distributions of the fragments (ρ_A_ and ρ_B_) at their final positions in the adduct, and the Pauli repulsion (E_Pauli_), that is the energy change associated with going from ρ_A_ + ρ_B_ to the antisymmetrized and renormalized wave function. The decomposition of E_st_ is not possible with ORCA 4.1.0, and it comprises the destabilizing interactions between the occupied orbitals; and, it is responsible for any steric repulsion. E_orb_ is the contribution arising from allowing the wave function to relax to the fully converged one, accounting for electron pair bonding, charge transfer and polarization, while E_disp_ is the contribution of the dispersion forces.

***Extended Transition State—Natural Orbital for Chemical Valence theory (ETS––NOCV) and Charge Displacement Function Analysis*** [85,86]. In the NOCV approach, the electron density rearrangement that takes place upon formation of AB from fragments A and B is defined with respect to a reference system made up of the occupied *ψ_i_^A^* and *ψ_i_^B^* orbitals of A and B orthonormalized with respect to each other (*ψ_i_*^0^). In other words, rather than two separate A and B determinants, their antisymmetrized product is taken as the fragment−fragment noninteracting reference (the so-called “promolecule”). The resulting electron density rearrangement in Equation (2),

(2) $$\Delta \rho_{tot}=\underset{i}{\sum }{\left|\psi_{i}^{AB}\right|}^{2}-{\left|\psi_{i}^{0}\right|}^{2}$$

where *ψ_i_^AB^* is the set of occupied orbitals of the adduct, can be brought into a diagonal form in terms of NOCVs. 

These are defined as the eigenfunctions, ***ϕ*_±*k*_**, of the so-called “valence operator” Equation (3) [87,88,89].

(3) $$\hat{V}=\underset{i}{\sum }\left(\left|\psi_{i}^{\left(AB\right)}\rangle \langle \psi_{i}^{\left(AB\right)}|-|\psi_{i}^{0}\rangle \langle \psi_{i}^{0}\right|\right)$$

The NOCVs can be grouped in pairs of complementary orbitals (***ϕ_k_***, ***ϕ_−k_***) corresponding to eigenvalues with the same absolute value but the opposite sign (Equation (4)).

(4) $$\hat{V}\varphi_{\pm k}=\pm \;\nu_{k}\varphi {\;}_{\pm \;k\;}(\nu_{k}>0)$$

where ***k*** numbers the NOCV pairs (***k*** = 0 for the largest value of ***ν_k_***). 

In this framework, **Δ*ρ*’** can be defined as in Equation (5).

(5) $$\Delta \rho^{\prime }=\underset{k}{\sum }\nu_{k}\left({\left|\varphi_{k}\right|}^{2}-{\left|\varphi_{-k}\right|}^{2}\right)=\underset{k}{\sum }\Delta \rho \prime_{k}$$

Hence, on formation of AB from the promolecule, a fraction ***ν_k_*** of electrons is transferred from the ***ϕ_−k_*** to the ***ϕ_k_*** orbital. Only some NOCV pairs have ***ν_k_*** significantly different from zero, and this subgroup is generally enough to describe the A⋯B interaction. For each value of ***k***, an energy contribution associated with the ***k***-th NOCV pair is given (***E_k_***).

The charge displacement function analysis [90,91] is based on Equation (6) on the relevant **Δ*ρ’_k_*** functions. The function **Δ*q***(***z’***) defines, at each point along a chosen axis, the amount of electron charge that, upon formation of the bond between the fragments, moves across a plane perpendicular to the axis through the point ***z’***. A positive (negative) value corresponds to electrons flowing in the direction of decreasing (increasing) z. Charge accumulates where the slope of **Δ*q*** is positive and decreases where it is negative.

(6) $$\Delta q_{k}\left(z\prime \right)=\int_{-\infty }^{+\infty }dx\int_{-\infty }^{+\infty }dy\int_{-\infty }^{z\prime }dz\;\Delta \rho_{k}$$

To extract a CT value from the **Δ*q*** curve, it is useful to fix a plausible boundary separating the fragments in the adducts (isoboundary). Unless otherwise specified, we chose the point on the ***z*** axis at which equal-valued isodensity surfaces of the isolated fragments are tangent. At this point, the value of **Δ*q_k_*** is represented by CT_k_.

## Figures and Tables

**Table 1:** Comparative view of IR and NMR data for complexes (**2–12**)CF_3_SO_3_. Δῦ (N≡C) = ῦ(complex)-ῦ(uncoordinated nitrile [49]). Δδ (N≡C) = δ (complex)-δ (uncoordinated nitrile [50]). ^a^ E isomer. ^b^ Z isomer. ^c^ Average value.

	IR (CH_2_Cl_2_), ῦ/cm^−1^					IR (Solid), ῦ/cm^−1^		^13^C NMR (Acetone-d_6_), δ/ppm						E/Z. Ratio
Comp.	ῦ (t-CO)	ῦ (μ-CO)	ῦ (μ-CN)	ῦ (N≡C)	Δῦ (N≡C)	ῦ (N≡C)	Δῦ (N≡C)	δ (μ-CN) ^a^	δ (μ-CN) ^b^	δ (N≡C) ^a^	δ (N≡C) ^b^	Δδ(N≡C) ^a^	Δδ(N≡C) ^b^	
**(2)^+^**	1985	1818	1561	2279	+25	2279	+26	330.5		132.0	131.8	+14.6	+14.4	1.9
**(3)^+^**	1984	1820	1559	2264	+31	2262	+27	330.7	330.2	139.8	140.0	+13.1	+13.3	2.3
**(4)^+^**	1984	1820	1560	2238	+8	2238	+9	329.7	330.0	131.2	131.6	+11.9	+12.3	1.2
**(5)^+^**	1985	1819	1561	2227	+12	2227	+17	330.9	330.4	133.4	133.9	+12.4	+12.9	1.1
**(6)^+^**	1982	1821	1561	2230	−7	2230	−3	329.2	329.0	129.2	129.6	+11.4	+11.8	1.3
**(7)^+^**	1983	1819	1559	2240	+7	2240	+6	329.9	329.7	130.4	130.7	+11.8	+12.1	1.0
**(8)^+^**	1974	1810	1558					334.5						1.7
**(9)^+^**	1965	1800	1532					331.7	332.0					1.3
**(10)^+^**	1984	1820	1560	2235	−2	2235	+2	342.0 ^c^	341.1 ^c^	129.1 ^c^	130.0 ^c^	+11.3 ^c^	+12.2 ^c^	2.3
**(11)^+^**	1987	1818	1568	2277	+23	2277	+24		333.4		132.1		+14.7	0.15
**(12)^+^**	1984	1817	1525	2281	+27	2281	+28	338.2	338.1	132.1	132.3	+14.7	+14.9	0.82

**Table 2:** Selected bond lengths (Å) and angles (°) for **(2Z)^+^**, **(3Z)^+^**, **(5Z)^+^** and **(11Z)^+^**.

	(2Z)^+^	(3Z)^+^	(5Z)^+^	(11Z)^+^
Fe(1)-Fe(2)	2.5100(10)	2.5091(9)	2.5023(7)	2.5107(7)
Fe(1)-C(1)	1.792(7)	1.761(3)	1.759(4)	1.754(4)
Fe(2)-N(2)	1.924(5)	1.928(3)	1.912(3)	1.918(3)
Fe(1)-C(2)	1.969(6)	1.964(3)	1.960(4)	1.947(4)
Fe(2)-C(2)	1.887(6)	1.910(3)	1.896(4)	1.914(4)
Fe(1)-C(3)	1.884(5)	1.880(3)	1.875(4)	1.880(3)
Fe(2)-C(3)	1.861(5)	1.863(3)	1.863(4)	1.871(4)
Fe(1)-Cp_av_	2.111(13)	2.112(7)	2.110(9)	2.110(7)
Fe(2)-Cp_av_	2.095(12)	2.112(7)	2.107(9)	2.102(7)
C(1)-O(1)	1.092(7)	1.141(4)	1.146(5)	1.143(5)
C(2)-O(2)	1.157(7)	1.171(4)	1.166(5)	1.166(5)
N(1)-C(3)	1.288(7)	1.297(4)	1.295(5)	1.290(5)
N(2)-C(21)	1.126(7)	1.140(4)	1.148(5)	1.133(5)
Fe(1)-C(1)-O(1)	177.4(6)	176.6(3)	176.9(3)	179.7(4)
Fe(2)-N(2)-C(21)	177.3(5)	175.6(2)	172.8(3)	173.6(3)
Fe(1)-C(2)-Fe(2)	81.2(2)	80.73(11)	80.91(14)	81.13(14)
Fe(1)-C(3)-Fe(2)	84.2(2)	84.18(12)	84.03(15)	84.03(14)
N(2)-C(21)-C(22)	178.3(6)	174.9(3)	178.7(4)	177.7(4)
Sum at N(1)	359.6(8)	360.0(3)	360.0(5)	360.0(5)
Sum at C(3)	359.5(6)	359.8(3)	359.3(4)	359.6(4)

**Table 3:** X-ray literature data for selected iron–nitrile complexes (with specified formal iron oxidation state) and 4-dimethylaminobenzonitrile. ^i^Pr = CH(CH_3_)_2_; PTA = 1,3,5-triaza-7-phosphaadamantane.

Complex	Fe–N (Å)	N≡C (Å)	Ref.
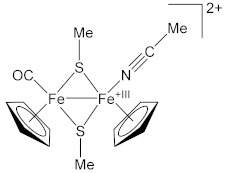	1.924(3)	1.141(4)	[59]
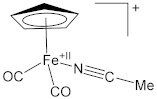	1.935(4)	1.147(6)	[60]
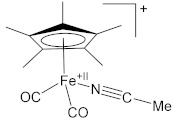	1.924(3)	1.143(5)	[61]
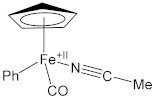	1.9025(11)	1.1468(17)	[62]
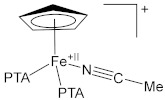	1.9099(19)	1.134(3)	[63]
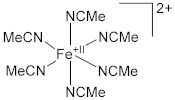	2.1510(12)	1.1369(17)	[64]
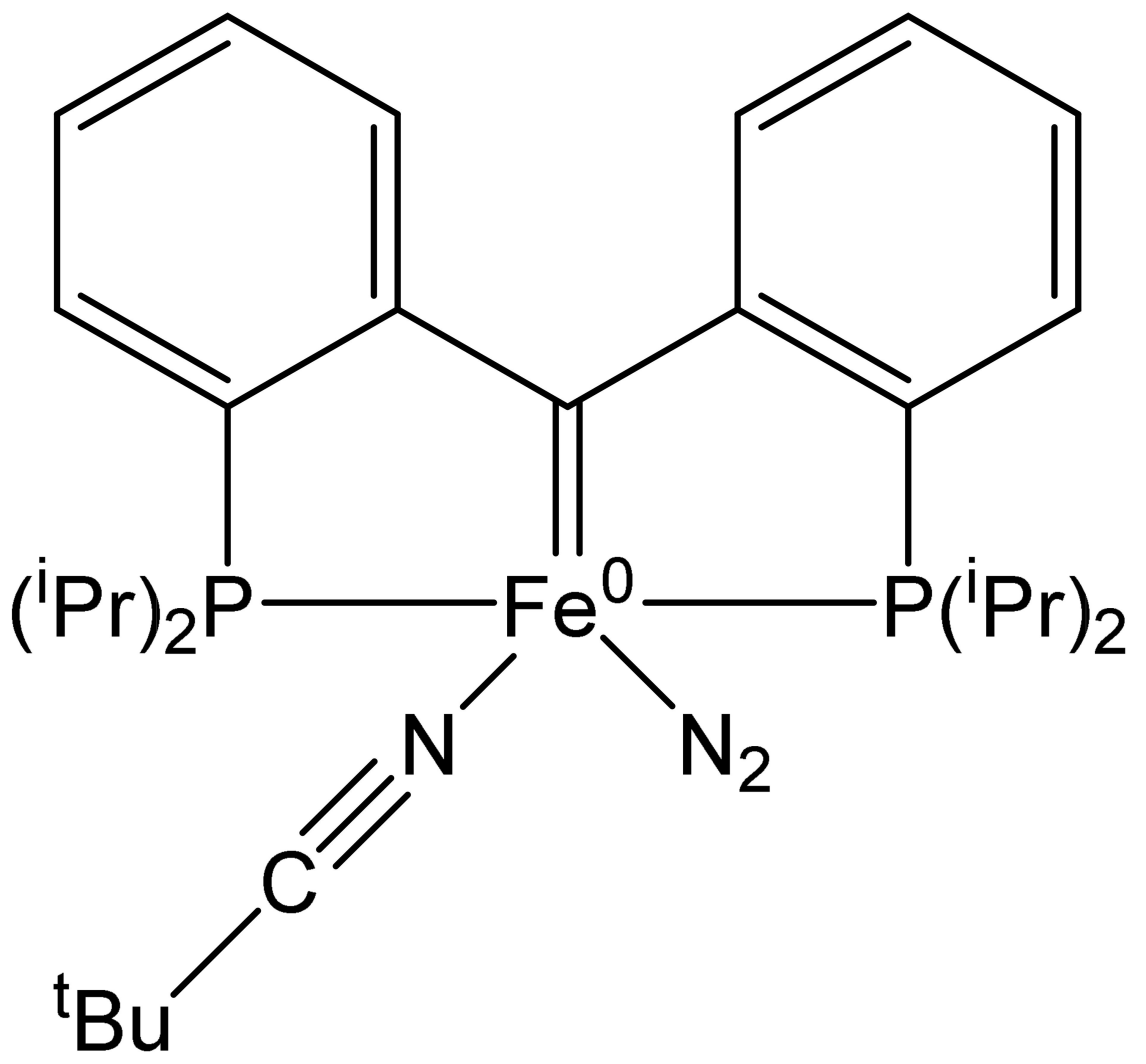	1.945(2)	1.147(3)	[65]
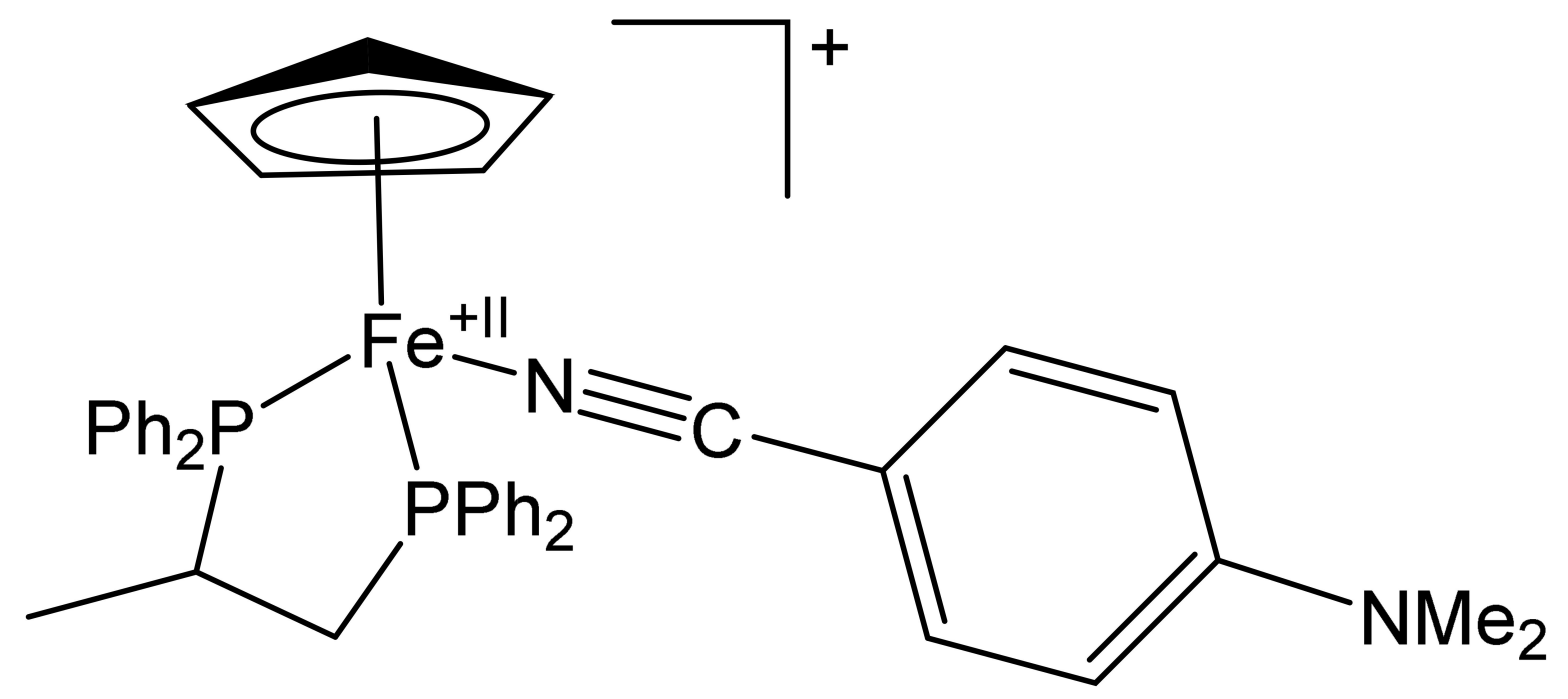	1.906(5)	1.146(8)	[16]
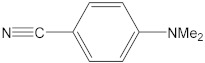		1.145(3)	[66]

**Table 4:** Calculated infrared wavenumber shift (Δῦ, in cm^−1^) upon coordination of nitriles to diiron complexes, bond dissociation energy (BDE, in kcal/mol), orbital contributions (ΔE_k_, in kcal/mol) and charge transfer (CT_k_) values (in electrons). k = 0 is related to σ donation, k = 1 and 2 are related to π back-donation. CT_tot_ is the net charge transfer; CT^π^_tot_ = CT_1_ + CT_2_. % of π-back-donation (% b.d.) calculated as: (ΔE_1_ + ΔE_2_)/ΔE_orb_. **(M1)^+^** = [Fe_2_Cp_2_(CO)(µ-CO){µ-CNMe_2_}(NCMe)]^+^, **(M2)^+^** = [Fe_2_Cp_2_(CO)(µ-CO){µ-CNMe(Cy)}(N≡CCF_3_)]^+^, **(M3)^+^** = [Fe_2_Cp_2_(CO)(µ-CO){µ-CNMe(Cy)}(N≡CCHF_2_)]^+^, **(M4)^+^** = [Fe_2_Cp_2_(CO)(µ-CO){µ-CNMe(Cy)}(N≡CCH_2_F)]^+^ (model complexes, see Figure S50).

Comp.	Δῦ	BDE	ΔE_0_ (CT_0_)	ΔE_1_ (CT_1_)	ΔE_2_ (CT_2_)	CT_tot_ (CT^π^_tot_)	CT_CN_ (CT^π^_CN_)	% b.d.
**(M1)^+^**	+2.3	−46.1	−33.2 (0.167)	−11.1 (−0.102)	−9.6 (−0.043)	0.034 (−0.145)	0.121 (0.065)	34.7
**(2Z)^+^_exp_ ^a^**	−	−	−30.2 (0.158)	−9.1 (−0.085)	−7.6 (−0.034)	0.048 (−0.119)	0.108 (0.063)	31.9
**(2Z)^+^**	+2.5	−46.3	−33.7 (0.167)	−11.5 (−0.107)	−9.6 (−0.038)	0.029 (−0.145)	0.111 (0.054)	34.8
**(2E)^+^**	+2.2	−46.6	−33.6 (0.168)	−11.4 (−0.106)	−9.6 (−0.043)	0.030 (−0.149)	0.113 (0.064)	35.0
**(3E)^+^**	+1.8	−50.0	−34.1 (0.173)	−11.2 (−0.100)	−9.5 (−0.043)	0.043 (−0.143)	0.113 (0.071)	33.7
**(4E)^+^**	−4.4	−50.9	−34.5 (0.176)	−13.5 (−0.126)	−10.1 (−0.044)	0.021 (−0.170)	0.116 (0.063)	36.6
**(5E)^+^**	1.2	−56.1	−35.4 (0.182)	−11.9 (−0.096)	−9.4 (−0.040)	0.062 (−0.136)	0.150 (0.102)	33.2
**(6E)^+^**	−16.2	−47.7	−33.9 (0.169)	−15.7 (−0.119)	−10.9 (−0.046)	0.005 (−0.165)	0.086 (0.032)	39.8
**(7E)^+^**	−5.0	−50.0	−34.5 (0.178)	−13.4 (−0.124)	−10.2 (−0.045)	0.024 (−0.169)	0.114 (0.062)	36.5
**(M2)^+^**	−62.7	−42.5	−32.5 (0.108)	−18.1 (−0.178)	−15.0 (−0.039)	−0.099 (−0.217)	0.032 (−0.051)	46.6
**(M3)^+^**	−48.9	−44.1	−33.2 (0.132)	−16.7 (−0.169)	−13.7 (−0.044)	−0.071 (−0.213)	0.049 (0.00)	44.0
**(M4)^+^**	−35.2	−46.0	−33.5 (0.154)	−16.1 (−0.159)	−11.0 (−0.046)	−0.040 (−0.205)	0.068 (0.011)	40.8

^a^ Not optimized experimental geometry.

**Table 5:** Crystal data and measurement details for **(2)CF_3_SO_3_**, **(3)CF_3_SO_3_**, **(5)CF_3_SO_3_** and **(11)CF_3_SO_3_**.

	(2)CF_3_SO_3_	(3)CF_3_SO_3_	(5)CF_3_SO_3_	(11)CF_3_SO_3_
Formula	C_23_H_27_F_3_Fe_2_N_2_O_5_S	C_26_H_33_F_3_Fe_2_N_2_O_5_S	C_30_H_34_F_3_Fe_2_N_3_O_5_S	C_20_H_21_F_3_Fe_2_N_2_O_5_S
FW	612.22	654.30	717.36	570.15
T, K	100(2)	100(2)	100(2)	100(2)
*λ*, Å	0.71073	0.71073	0.71073	0.71073
Crystal system	Monoclinic	Monoclinic	Monoclinic	Orthorhombic
Space group	P*2_1_*/*n*	P*2_1_*/*n*	C*2*/*c*	P*bca*
*a*, Å	10.9065(9)	12.081(4)	28.694(3)	15.1677(7)
*b*, Å	14.8064(11)	17.491(6)	10.6013(9)	13.5839(6)
*c*, Å	15.9959(12)	13.857(4)	23.210(2)	22.1751(10)
*β*	101.382(3)	107.13(2)	116.562(3)	90
Cell Volume, Å^3^	2532.3(3)	2798.2(16)	6315.0(10)	4568.9(4)
Z	4	4	8	8
*D_c_*, g∙cm^−3^	1.606	1.553	1.509	1.658
µ, mm^−1^	1.287	1.170	1.046	1.420
F(000)	1256	1352	2960	2320
Crystal size, mm	0.16 × 0.14 × 0.11	0.22 × 0.20 × 0.10	0.18 × 0.16 × 0.12	0.18 × 0.16 × 0.11
θ limits	1.892–25.027	1.929–27.000	1.962–26.000	1.837–25.999
Reflections collected	44,496	37,875	39,670	59,100
Independent reflections	4453 [*R_int_* = 0.1492]	6110 [*R_int_* = 0.0453]	6200 [*R_int_* = 0.0435]	4467 [*R_int_* = 0.0805]
Data/restraints/parameters	4453/0/328	6110/24/356	6200/175/454	4467/0/300
Goodness on fit on F^2^	1.054	1.127	1.084	1.252
*R*_1_ (*I* > 2σ(*I*))	0.0597	0.0485	0.0581	0.0532
*wR*_2_ (all data)	0.1606	0.1056	0.16030	0.1085
Largest diff. peak and hole, e Å^−3^	0.966/–0.694	0.830/–0.579	2.211/–0.881	0.803/–0.469

## Data Availability

Not applicable.

## Supplementary Materials

The following are available online: NMR spectra (Figures S1–S25); IR spectra (Figures S26–S47); isodensity surface plots (Figure S48); view of DFT optimized structures (Figures S49–S50); DFT data. CCDC reference numbers 2088526 (**(****2)CF_3_SO_3_**), 2088527 **((****3)CF_3_SO_3_**), 2088528 **((****5)CF_3_SO_3_**) and 2088529 (**(****11)CF_3_SO_3_**) contain the supplementary crystallographic data for the X-ray studies reported in this paper. These data can be obtained free of charge at www.ccdc.cam.ac.uk/conts/retrieving.html (or from the Cambridge Crystallographic Data Centre, 12, Union Road, Cambridge CB2 1EZ, UK; fax: (internat.) +44-1223/336-033; e-mail: deposit@ccdc.cam.ac.uk).

## Sample Availability

Samples of the complexes reported in this paper are available from the authors.
