# Supplementary material for: A Comprehensive Analysis of the Metal–Nitrile Bonding in an Organo-Diiron System
Source: Molecules. 2021 Nov 23;26(23):7088. doi: 10.3390/molecules26237088 (PMC8659010; doi:10.3390/molecules26237088)

checkCIF/PLATON report

Structure factors have been supplied for datablock(s) 11, 2, 3, 5

THIS REPORT IS FOR GUIDANCE ONLY. IF USED AS PART OF A REVIEW PROCEDURE FOR PUBLICATION, IT SHOULD NOT REPLACE THE EXPERTISE OF AN EXPERIENCED CRYSTALLOGRAPHIC REFEREE.

No syntax errors found. CIF dictionary Interpreting this report

Datablock: 2

Bond precision:	C-C = 0.0092 A		Wavelength=0.71073
Cell:	a=10.9065(9)	b=14.8064(11)	c=15.9959(12)
	alpha=90	beta=101.382(3)	gamma=90
Temperature:	100 K		

	Calculated	Reported
Volume	2532.3(3)	2532.3(3)
Space group	P 21/n	P 21/n
Hall group	-P 2yn	-P 2yn
Moiety formula	C22 H27 Fe2 N2 O2, C F3 O3 S	?
Sum formula	C23 H27 F3 Fe2 N2 O5 S	C23 H27 F3 Fe2 N2 O5 S
Mr	612.23	612.22
Dx,g cm-3	1.606	1.606
Z	4	4
Mu (mm-1)	1.287	1.287
F000	1256.0	1256.0
F000’	1259.84	
h,k,lmax	12,17,19	12,17,19
Nref	4461	4453
Tmin,Tmax	0.814,0.868	0.421,0.563
Tmin’	0.814	

Correction method= # Reported T Limits: Tmin=0.421 Tmax=0.563
AbsCorr = EMPIRICAL

Data completeness= 0.998 Theta(max)= 25.027

R(reflections)= 0.0597(3618) wR2(reflections)= 0.1606(4453)

S = 1.054 Npar= 328

The following ALERTS were generated. Each ALERT has the format
test-name_ALERT_alert-type_alert-level.
Click on the hyperlinks for more details of the test.

Alert level C

```
RINTA01_ALERT_3_C  The value of Rint is greater than 0.12
            Rint given   0.149
PLAT213_ALERT_2_C Atom C103            has ADP max/min Ratio .....        3.1 oblate
PLAT213_ALERT_2_C Atom C201            has ADP max/min Ratio .....        3.5 prolat
PLAT220_ALERT_2_C NonSolvent   Resd 1  C   Ueq(max)/Ueq(min) Range        4.0 Ratio
PLAT244_ALERT_4_C Low    'Solvent' Ueq as Compared to Neighbors of         S1 Check
PLAT341_ALERT_3_C Low Bond Precision on  C-C Bonds ...............    0.00924 Ang.
PLAT906_ALERT_3_C Large K Value in the Analysis of Variance ......      5.133 Check
PLAT911_ALERT_3_C Missing FCF Refl Between Thmin & STh/L=    0.595          8 Report
```

Alert level G

```
PLAT083_ALERT_2_G SHELXL Second Parameter in WGHT  Unusually Large      10.84 Why ?
PLAT232_ALERT_2_G Hirshfeld Test Diff (M-X)  Fe1      --C1       .        7.3 s.u.
PLAT244_ALERT_4_G Low    'Solvent' Ueq as Compared to Neighbors of       C301 Check
PLAT343_ALERT_2_G Unusual sp?      Angle Range in Main Residue for         C2 Check
PLAT343_ALERT_2_G Unusual sp?      Angle Range in Main Residue for         C3 Check
PLAT870_ALERT_4_G ALERTS Related to Twinning Effects Suppressed ..          ! Info
PLAT883_ALERT_1_G No Info/Value for _atom_sites_solution_primary .     Please Do !
PLAT909_ALERT_3_G Percentage of I>2sig(I) Data at Theta(Max) Still        73% Note
PLAT931_ALERT_5_G CIFcalcFCF  Twin Law ( 1 0-1)         Est.d BASF       0.30 Check
PLAT965_ALERT_2_G The SHELXL WEIGHT Optimisation has not Converged     Please Check
```

---

0 **ALERT level A** = Most likely a serious problem - resolve or explain
0 **ALERT level B** = A potentially serious problem, consider carefully
8 **ALERT level C** = Check. Ensure it is not caused by an omission or oversight
10 **ALERT level G** = General information/check it is not something unexpected

1 ALERT type 1 CIF construction/syntax error, inconsistent or missing data
8 ALERT type 2 Indicator that the structure model may be wrong or deficient
5 ALERT type 3 Indicator that the structure quality may be low
3 ALERT type 4 Improvement, methodology, query or suggestion
1 ALERT type 5 Informative message, check

Datablock: 3

Bond precision:	C-C = 0.0048 A	Wavelength=0.71073	
Cell:	a=12.081(4)	b=17.491(6)	c=13.857(4)
	alpha=90	beta=107.13(2)	gamma=90
Temperature:	100 K		

	Calculated	Reported
Volume	2798.2(16)	2798.2(16)
Space group	P 21/n	P 21/n
Hall group	-P 2yn	-P 2yn
Moiety formula	C25 H33 Fe2 N2 O2, C F3 O3 S	?
Sum formula	C26 H33 F3 Fe2 N2 O5 S	C26 H33 F3 Fe2 N2 O5 S
Mr	654.30	654.30
Dx,g cm-3	1.553	1.553
Z	4	4
Mu (mm-1)	1.170	1.170
F000	1352.0	1352.0
F000’	1355.87	
h,k,lmax	15,22,17	15,22,17
Nref	6113	6110
Tmin,Tmax	0.773,0.890	0.311,0.434
Tmin’	0.773	

Correction method= # Reported T Limits: Tmin=0.311 Tmax=0.434
AbsCorr = EMPIRICAL

Data completeness= 1.000 Theta(max)= 27.000

R(reflections)= 0.0485(5340) wR2(reflections)= 0.1056(6110)

S = 1.127 Npar= 356

The following ALERTS were generated. Each ALERT has the format
test-name_ALERT_alert-type_alert-level.
Click on the hyperlinks for more details of the test.

Alert level C

```
PLAT213_ALERT_2_C Atom C205            has ADP max/min Ratio .....        3.5 prolat
PLAT220_ALERT_2_C NonSolvent   Resd 1  C   Ueq(max)/Ueq(min) Range        3.4 Ratio
PLAT244_ALERT_4_C Low    ’Solvent’ Ueq as Compared to Neighbors of         S1 Check
PLAT250_ALERT_2_C Large U3/U1 Ratio for Average U(i,j) Tensor ....        3.5 Note
PLAT906_ALERT_3_C Large K Value in the Analysis of Variance ......      4.045 Check
```

Alert level G

```
PLAT003_ALERT_2_G Number of Uiso or Uij Restrained non-H Atoms ...          4 Report
PLAT083_ALERT_2_G SHELXL Second Parameter in WGHT  Unusually Large       7.34 Why ?
PLAT186_ALERT_4_G The CIF-Embedded .res File Contains ISOR Records          2 Report
PLAT232_ALERT_2_G Hirshfeld Test Diff (M-X)  Fe1      --C1       .        5.6 s.u.
PLAT232_ALERT_2_G Hirshfeld Test Diff (M-X)  Fe2      --N2       .        5.2 s.u.
PLAT244_ALERT_4_G Low    ’Solvent’ Ueq as Compared to Neighbors of       C301 Check
PLAT343_ALERT_2_G Unusual sp?      Angle Range in Main Residue for         C2 Check
PLAT343_ALERT_2_G Unusual sp?      Angle Range in Main Residue for         C3 Check
PLAT860_ALERT_3_G Number of Least-Squares Restraints .............         24 Note
PLAT883_ALERT_1_G No Info/Value for _atom_sites_solution_primary .     Please Do !
PLAT912_ALERT_4_G Missing # of FCF Reflections Above STh/L=  0.600          3 Note
PLAT978_ALERT_2_G Number C-C Bonds with Positive Residual Density.          2 Info
```

```
   0 ALERT level A = Most likely a serious problem - resolve or explain
   0 ALERT level B = A potentially serious problem, consider carefully
   5 ALERT level C = Check. Ensure it is not caused by an omission or oversight
  12 ALERT level G = General information/check it is not something unexpected

   1 ALERT type 1 CIF construction/syntax error, inconsistent or missing data
  10 ALERT type 2 Indicator that the structure model may be wrong or deficient
   2 ALERT type 3 Indicator that the structure quality may be low
   4 ALERT type 4 Improvement, methodology, query or suggestion
   0 ALERT type 5 Informative message, check
```

Datablock: 5

Bond precision: C-C = 0.0066 A Wavelength=0.71073

Cell:	a=28.694(3)	b=10.6013(9)	c=23.210(2)
	alpha=90	beta=116.562(3)	gamma=90
Temperature:	100 K		

	Calculated	Reported
Volume	6315.1(10)	6315.0(10)
Space group	C 2/c	C 2/c
Hall group	-C 2yc	-C 2yc
Moiety formula	C29 H34 Fe2 N3 O2, C F3 O3 S	?
Sum formula	C30 H34 F3 Fe2 N3 O5 S	C30 H34 F3 Fe2 N3 O5 S
Mr	717.36	717.36
Dx,g cm-3	1.509	1.509
Z	8	8
Mu (mm-1)	1.046	1.046
F000	2960.0	2960.0
F000’	2967.82	
h,k,lmax	35,13,28	35,13,28
Nref	6210	6200
Tmin,Tmax	0.828,0.882	0.468,0.564
Tmin’	0.828	

Correction method= # Reported T Limits: Tmin=0.468 Tmax=0.564
AbsCorr = EMPIRICAL

Data completeness= 0.998 Theta(max)= 26.000

R(reflections)= 0.0581(5623) wR2(reflections)= 0.1603(6200)

S = 1.084 Npar= 454

The following ALERTS were generated. Each ALERT has the format
test-name_ALERT_alert-type_alert-level.
Click on the hyperlinks for more details of the test.

Alert level C

```
DIFMX02_ALERT_1_C  The maximum difference density is > 0.1*ZMAX*0.75
            The relevant atom site should be identified.
PLAT094_ALERT_2_C Ratio of Maximum / Minimum Residual Density ....       2.51 Report
PLAT097_ALERT_2_C Large Reported Max.  (Positive) Residual Density       2.21 eA-3
PLAT220_ALERT_2_C NonSolvent   Resd 1  C   Ueq(max)/Ueq(min) Range        3.4 Ratio
PLAT250_ALERT_2_C Large U3/U1 Ratio for Average U(i,j) Tensor ....        2.1 Note
PLAT341_ALERT_3_C Low Bond Precision on  C-C Bonds ...............    0.00661 Ang.
PLAT906_ALERT_3_C Large K Value in the Analysis of Variance ......      3.807 Check
PLAT911_ALERT_3_C Missing FCF Refl Between Thmin & STh/L=    0.600          7 Report
PLAT971_ALERT_2_C Check Calcd Resid. Dens.  1.46A   From C311            2.14 eA-3
```

Alert level G

```
PLAT002_ALERT_2_G Number of Distance or Angle Restraints on AtSite          8 Note
PLAT003_ALERT_2_G Number of Uiso or Uij Restrained non-H Atoms ...         16 Report
PLAT083_ALERT_2_G SHELXL Second Parameter in WGHT  Unusually Large      45.75 Why ?
PLAT128_ALERT_4_G Alternate Setting for Input Space Group     C2/c       I2/a Note
PLAT172_ALERT_4_G The CIF-Embedded .res File Contains DFIX Records          3 Report
PLAT175_ALERT_4_G The CIF-Embedded .res File Contains SAME Records          4 Report
PLAT178_ALERT_4_G The CIF-Embedded .res File Contains SIMU Records          2 Report
PLAT186_ALERT_4_G The CIF-Embedded .res File Contains ISOR Records          2 Report
PLAT232_ALERT_2_G Hirshfeld Test Diff (M-X)  Fe1      --C1       .        5.8 s.u.
PLAT300_ALERT_4_G Atom Site Occupancy of S1         Constrained at        0.5 Check
PLAT300_ALERT_4_G Atom Site Occupancy of F11        Constrained at        0.5 Check
PLAT300_ALERT_4_G Atom Site Occupancy of F12        Constrained at        0.5 Check
PLAT300_ALERT_4_G Atom Site Occupancy of F13        Constrained at        0.5 Check
PLAT300_ALERT_4_G Atom Site Occupancy of O11        Constrained at        0.5 Check
PLAT300_ALERT_4_G Atom Site Occupancy of O12        Constrained at        0.5 Check
PLAT300_ALERT_4_G Atom Site Occupancy of O13        Constrained at        0.5 Check
PLAT300_ALERT_4_G Atom Site Occupancy of C301       Constrained at        0.5 Check
PLAT300_ALERT_4_G Atom Site Occupancy of S2         Constrained at        0.5 Check
PLAT300_ALERT_4_G Atom Site Occupancy of F21        Constrained at        0.5 Check
PLAT300_ALERT_4_G Atom Site Occupancy of F22        Constrained at        0.5 Check
PLAT300_ALERT_4_G Atom Site Occupancy of F23        Constrained at        0.5 Check
PLAT300_ALERT_4_G Atom Site Occupancy of O21        Constrained at        0.5 Check
PLAT300_ALERT_4_G Atom Site Occupancy of O22        Constrained at        0.5 Check
PLAT300_ALERT_4_G Atom Site Occupancy of O23        Constrained at        0.5 Check
PLAT300_ALERT_4_G Atom Site Occupancy of C311       Constrained at        0.5 Check
PLAT302_ALERT_4_G Anion/Solvent/Minor-Residue Disorder (Resd  2  )       100% Note
PLAT302_ALERT_4_G Anion/Solvent/Minor-Residue Disorder (Resd  3  )       100% Note
PLAT343_ALERT_2_G Unusual sp?      Angle Range in Main Residue for         C2 Check
PLAT343_ALERT_2_G Unusual sp?      Angle Range in Main Residue for         C3 Check
PLAT432_ALERT_2_G Short Inter X...Y Contact  C103     ..C103             3.17 Ang.
                                               1-x,2-y,2-z  =      5_677 Check
PLAT789_ALERT_4_G Atoms with Negative _atom_site_disorder_group  #         16 Check
PLAT860_ALERT_3_G Number of Least-Squares Restraints .............        175 Note
PLAT883_ALERT_1_G No Info/Value for _atom_sites_solution_primary .     Please Do !
PLAT910_ALERT_3_G Missing # of FCF Reflection(s) Below Theta(Min).          2 Note
PLAT912_ALERT_4_G Missing # of FCF Reflections Above STh/L=  0.600          1 Note
PLAT933_ALERT_2_G Number of OMIT Records in Embedded .res File ...          7 Note
PLAT978_ALERT_2_G Number C-C Bonds with Positive Residual Density.          4 Info
```

---

0 **ALERT level A** = Most likely a serious problem - resolve or explain
0 **ALERT level B** = A potentially serious problem, consider carefully

```
 9 ALERT level C = Check. Ensure it is not caused by an omission or oversight
37 ALERT level G = General information/check it is not something unexpected

 2 ALERT type 1 CIF construction/syntax error, inconsistent or missing data
14 ALERT type 2 Indicator that the structure model may be wrong or deficient
 5 ALERT type 3 Indicator that the structure quality may be low
25 ALERT type 4 Improvement, methodology, query or suggestion
 0 ALERT type 5 Informative message, check
```

Datablock: 11

Bond precision: C-C = 0.0062 A Wavelength=0.71073

Cell:	a=15.1677(7)	b=13.5839(6)	c=22.1751(10)
	alpha=90	beta=90	gamma=90
Temperature:	100 K		

	Calculated	Reported
Volume	4568.9(4)	4568.9(4)
Space group	P b c a	P b c a
Hall group	-P 2ac 2ab	-P 2ac 2ab
Moiety formula	C19 H21 Fe2 N2 O2, C F3 O3 S	?
Sum formula	C20 H21 F3 Fe2 N2 O5 S	C20 H21 F3 Fe2 N2 O5 S
Mr	570.15	570.15
Dx,g cm-3	1.658	1.658
Z	8	8
Mu (mm-1)	1.420	1.420
F000	2320.0	2320.0
F000’	2327.63	
h,k,lmax	18,16,27	18,16,27
Nref	4486	4467
Tmin,Tmax	0.774,0.985	0.435,0.563
Tmin’	0.774	

Correction method= # Reported T Limits: Tmin=0.435 Tmax=0.563
AbsCorr = EMPIRICAL

Data completeness= 0.996 Theta(max)= 25.999

R(reflections)= 0.0532(4138) wR2(reflections)= 0.1085(4467)

S = 1.252 Npar= 300

The following ALERTS were generated. Each ALERT has the format
test-name_ALERT_alert-type_alert-level.
Click on the hyperlinks for more details of the test.

Alert level C

```
PLAT220_ALERT_2_C NonSolvent   Resd 1  C   Ueq(max)/Ueq(min) Range       3.9 Ratio
PLAT341_ALERT_3_C Low Bond Precision on  C-C Bonds ...............   0.00615 Ang.
PLAT906_ALERT_3_C Large K Value in the Analysis of Variance ......     6.286 Check
PLAT911_ALERT_3_C Missing FCF Refl Between Thmin & STh/L=    0.600        12 Report
PLAT934_ALERT_3_C Number of (Iobs-Icalc)/Sigma(W) > 10 Outliers ..         1 Check
PLAT975_ALERT_2_C Check Calcd Resid. Dens.  1.01A   From O12            0.53 eA-3
PLAT975_ALERT_2_C Check Calcd Resid. Dens.  0.92A   From O11            0.42 eA-3
```

Alert level G

```
PLAT083_ALERT_2_G SHELXL Second Parameter in WGHT  Unusually Large     15.24 Why ?
PLAT343_ALERT_2_G Unusual sp?      Angle Range in Main Residue for        C2 Check
PLAT343_ALERT_2_G Unusual sp?      Angle Range in Main Residue for        C3 Check
PLAT883_ALERT_1_G No Info/Value for _atom_sites_solution_primary .    Please Do !
PLAT912_ALERT_4_G Missing # of FCF Reflections Above STh/L=  0.600         8 Note
PLAT965_ALERT_2_G The SHELXL WEIGHT Optimisation has not Converged    Please Check
PLAT978_ALERT_2_G Number C-C Bonds with Positive Residual Density.         0 Info
```

```
   0 ALERT level A = Most likely a serious problem - resolve or explain
   0 ALERT level B = A potentially serious problem, consider carefully
   7 ALERT level C = Check. Ensure it is not caused by an omission or oversight
   7 ALERT level G = General information/check it is not something unexpected

   1 ALERT type 1 CIF construction/syntax error, inconsistent or missing data
   8 ALERT type 2 Indicator that the structure model may be wrong or deficient
   4 ALERT type 3 Indicator that the structure quality may be low
   1 ALERT type 4 Improvement, methodology, query or suggestion
   0 ALERT type 5 Informative message, check
```

It is advisable to attempt to resolve as many as possible of the alerts in all categories. Often the minor alerts point to easily fixed oversights, errors and omissions in your CIF or refinement strategy, so attention to these fine details can be worthwhile. In order to resolve some of the more serious problems it may be necessary to carry out additional measurements or structure refinements. However, the purpose of your study may justify the reported deviations and the more serious of these should normally be commented upon in the discussion or experimental section of a paper or in the "special_details" fields of the CIF. checkCIF was carefully designed to identify outliers and unusual parameters, but every test has its limitations and alerts that are not important in a particular case may appear. Conversely, the absence of alerts does not guarantee there are no aspects of the results needing attention. It is up to the individual to critically assess their own results and, if necessary, seek expert advice.

Publication of your CIF in IUCr journals

A basic structural check has been run on your CIF. These basic checks will be run on all CIFs submitted for publication in IUCr journals (*Acta Crystallographica*, *Journal of Applied Crystallography*, *Journal of Synchrotron Radiation*); however, if you intend to submit to *Acta Crystallographica Section C* or *E* or *IUCrData*, you should make sure that full publication checks are run on the final version of your CIF prior to submission.

Publication of your CIF in other journals

Please refer to the *Notes for Authors* of the relevant journal for any special instructions relating to CIF submission.

PLATON version of 03/06/2021; check.def file version of 02/06/2021

Datablock 2 - ellipsoid plot

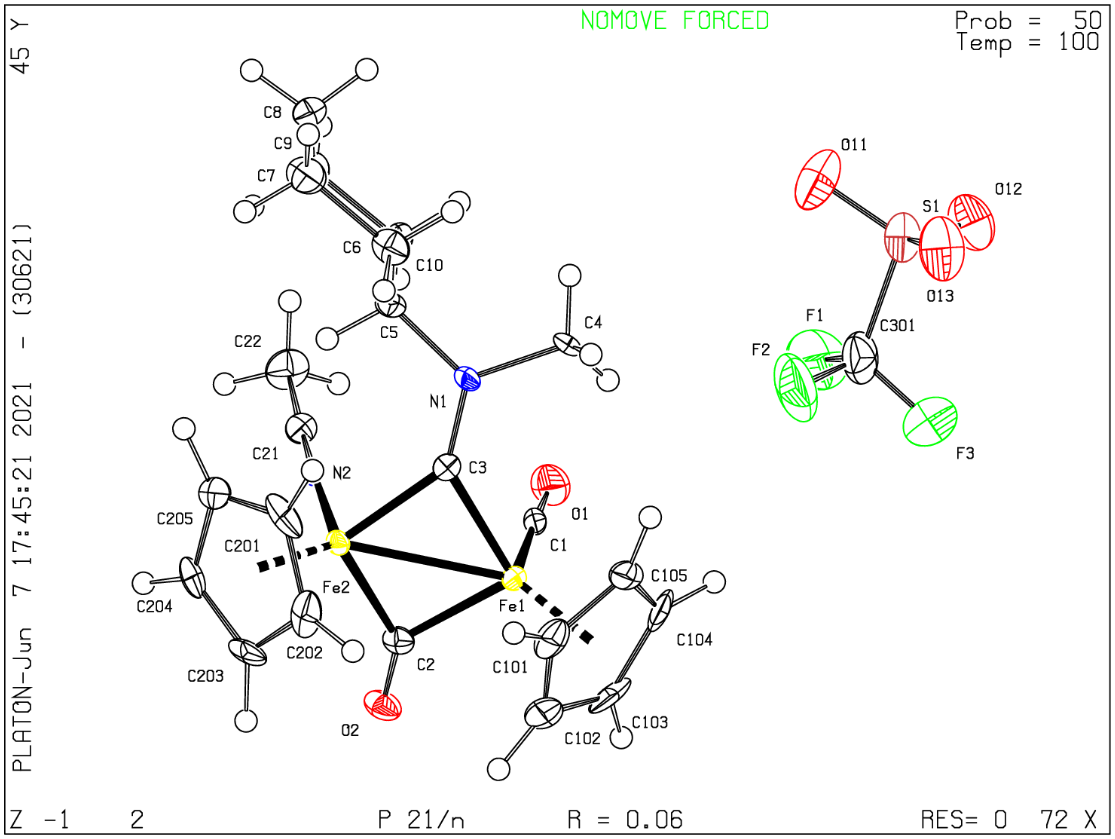

Datablock 3 - ellipsoid plot

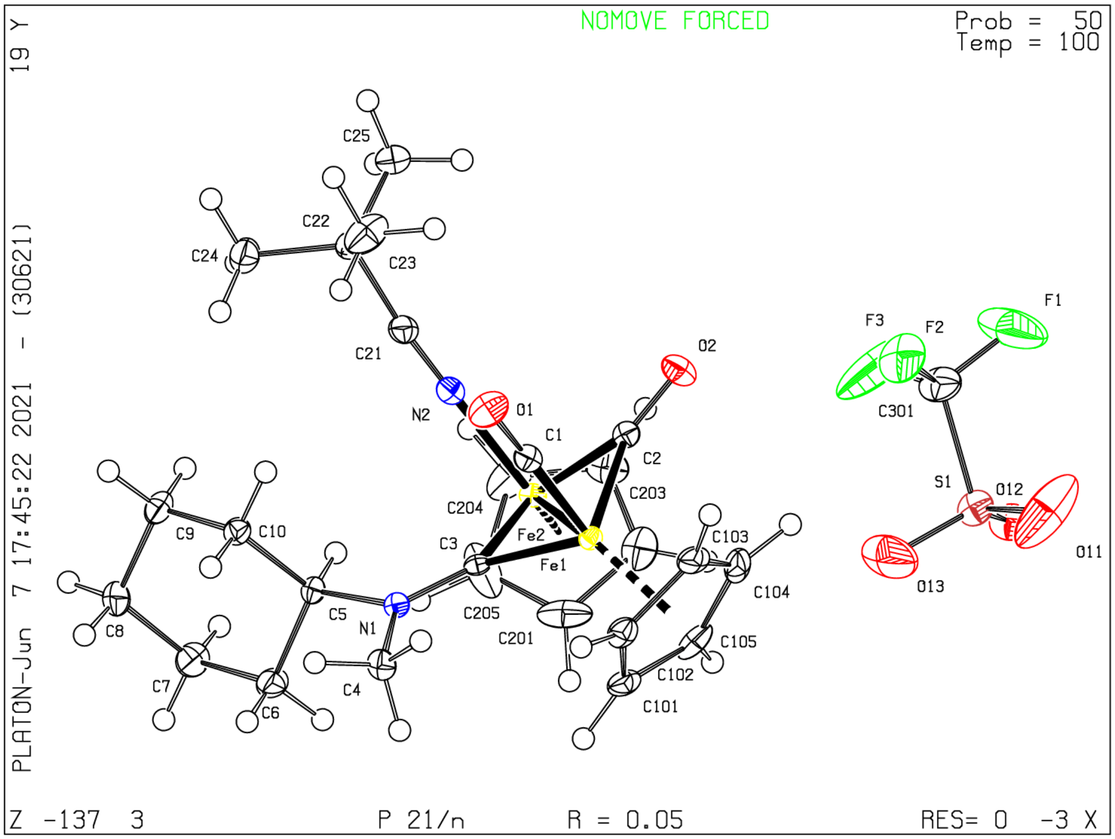

Datablock 5 - ellipsoid plot

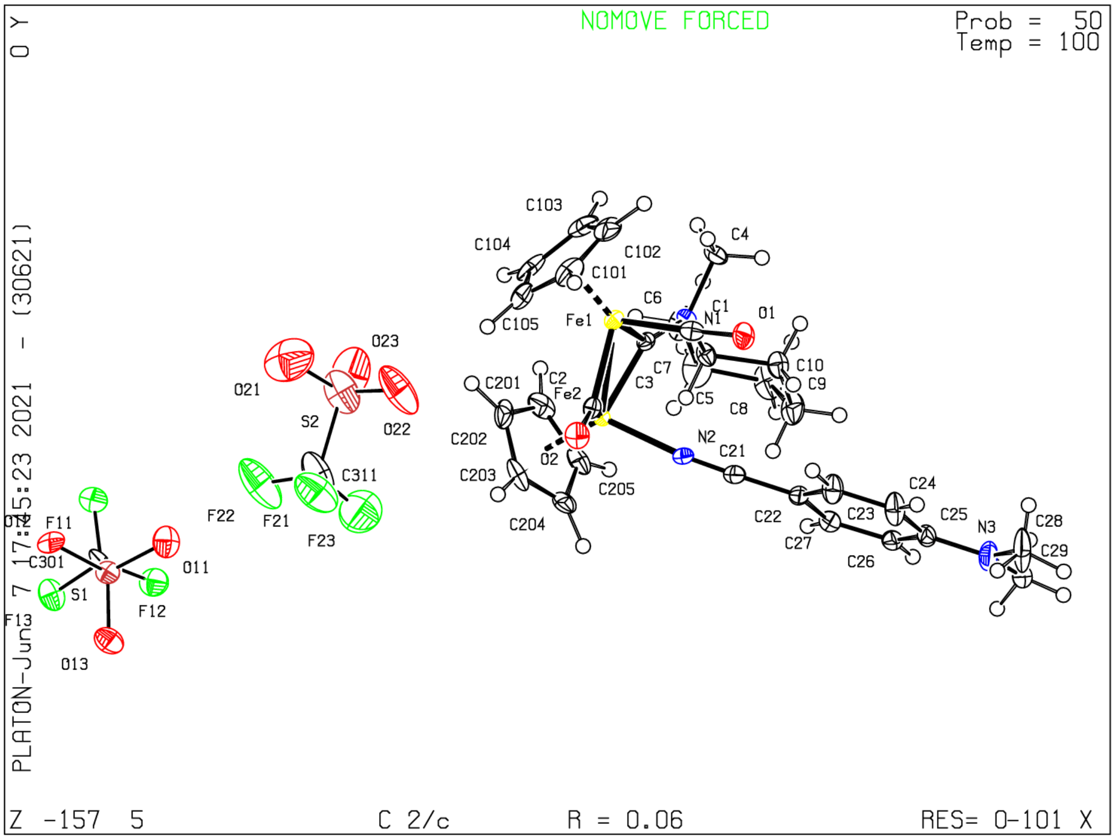

Datablock 11 - ellipsoid plot

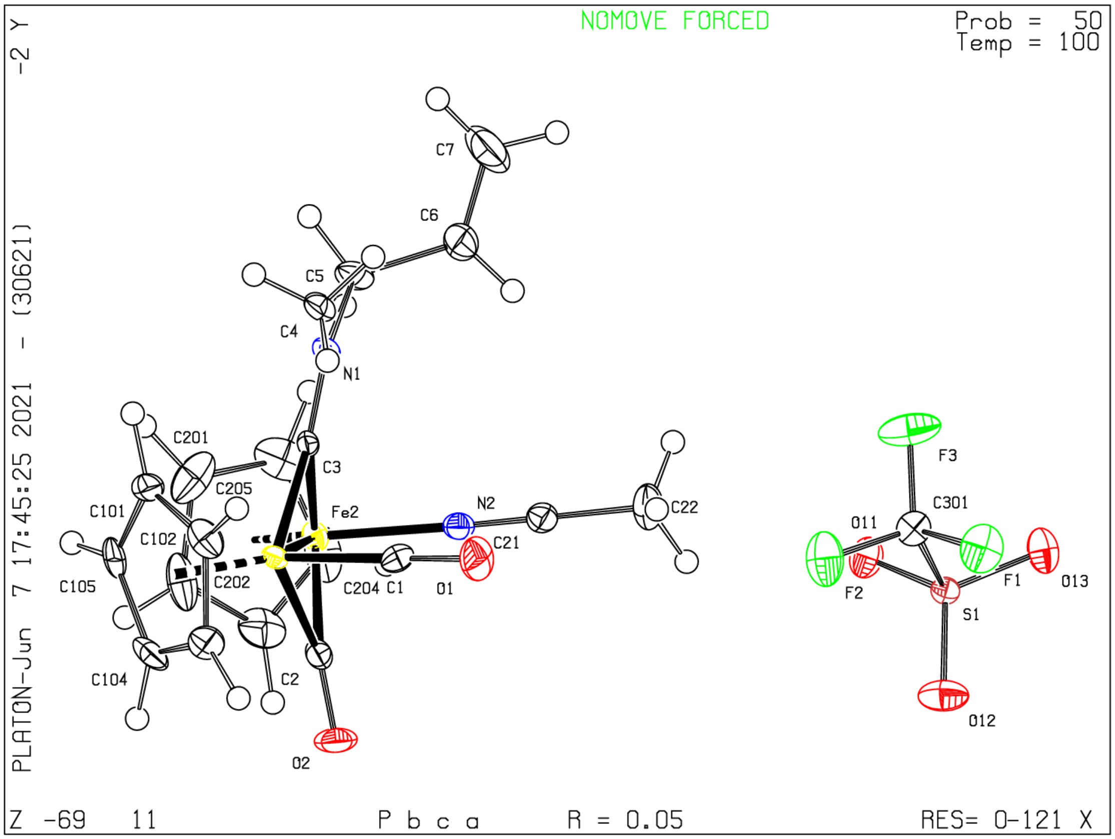

Supplement: Supplementary file 1 [file molecules-26-07088-s001.zip › molecules-1472803-supplementary/checkcif.pdf]
