# Supplementary material for: A Comprehensive Analysis of the Metal–Nitrile Bonding in an Organo-Diiron System
Source: Molecules. 2021 Nov 23;26(23):7088. doi: 10.3390/molecules26237088 (PMC8659010; doi:10.3390/molecules26237088)
Supplement: Supplementary file 1 [file molecules-26-07088-s001.zip › molecules-1472803-supplementary/Supporting Information.pdf]

A Comprehensive Analysis of the Metal-Nitrile Bonding in an Organo-Diiron System

Giulio Bresciani, Lorenzo Biancalana, Guido Pampaloni, Stefano Zacchini, Gianluca Ciancaleoni, Fabio Marchetti

Supporting Information

Table of contents *Pages*

**Figures S1-S25**: NMR spectra	S2-S13
**Figures S26-S47**: IR spectra	S14-S24
**Figure S48**: Alternative isodensity surface plots for [**M1**]$^+$	S25
**Figure S49**: View of DFT structures of experimental complexes	S26
**Figure S50**: View of DFT structures of model complexes	S27
**DFT optimized geometries**	S28-S38

Figure S1. ^{1}H NMR spectrum (401 MHz, acetone-d$_6$) of **[2]CF$_3$SO$_3$**.

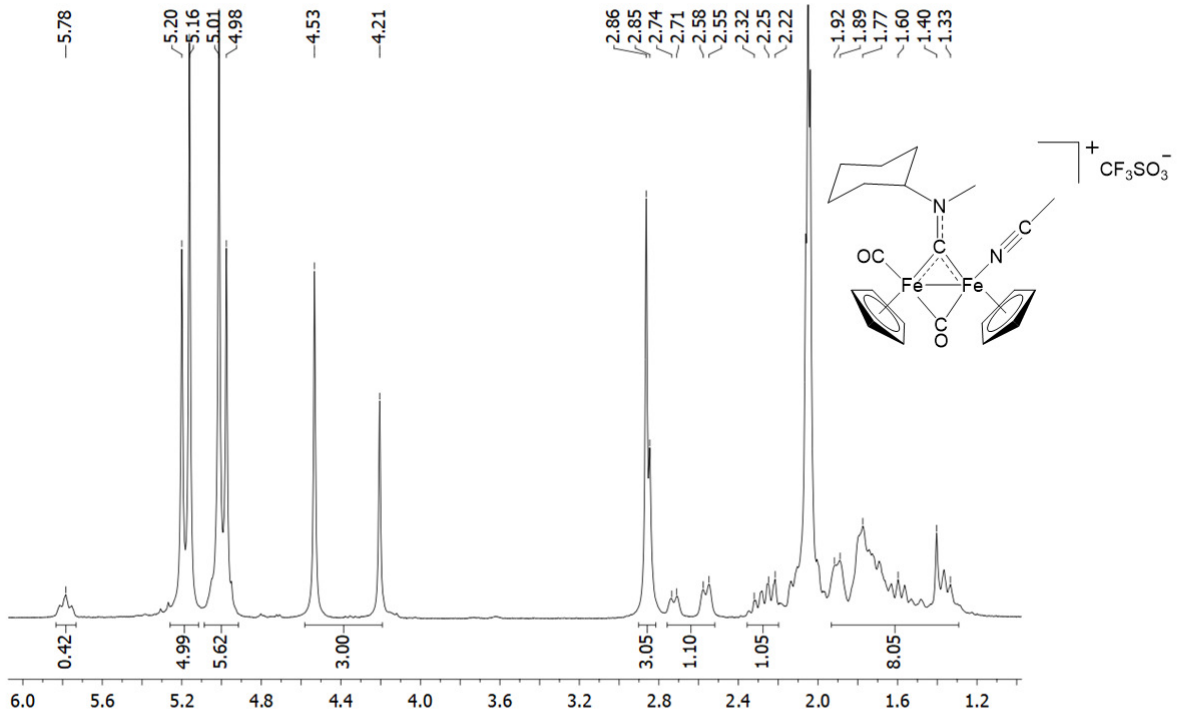

Figure S2. ^{13}C{^{1}H} NMR spectrum (101 MHz, acetone-d$_6$) of **[2]CF$_3$SO$_3$**.

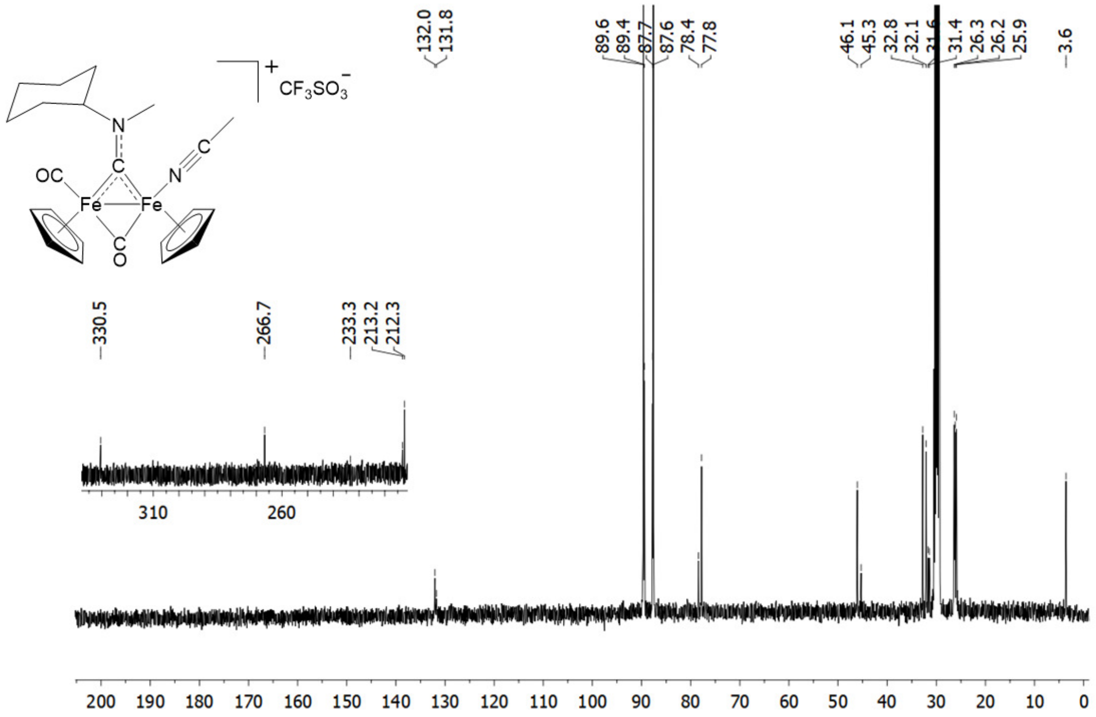

Figure S3. ^{1}H NMR spectrum (401 MHz, acetone-d$_6$) of **[3]CF$_3$SO$_3$**.

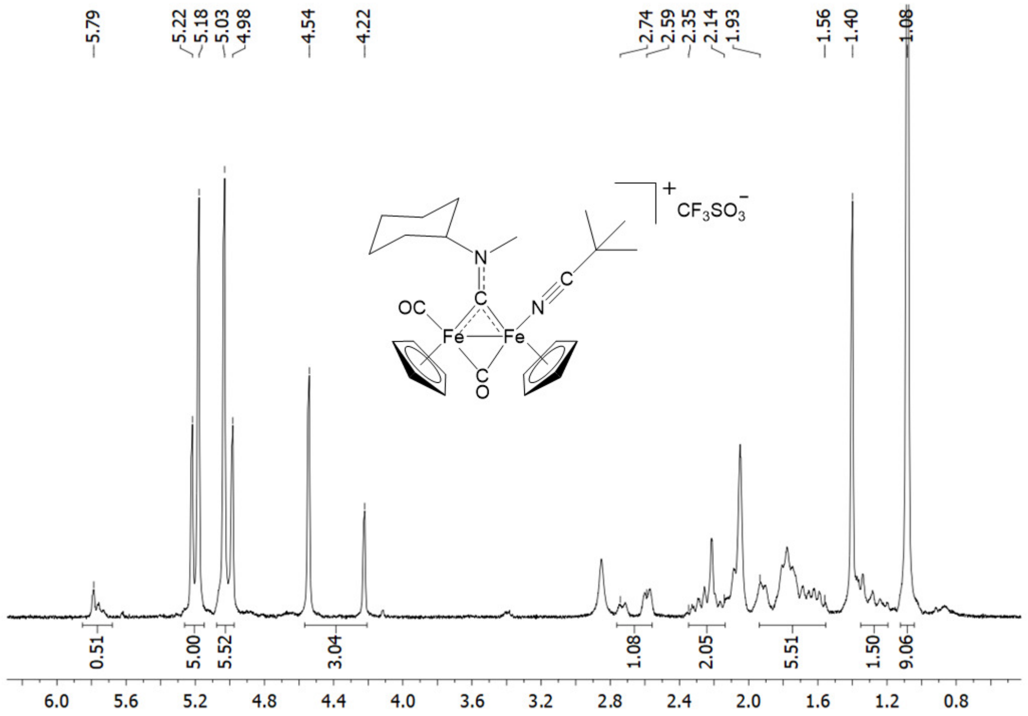

Figure S4. $^{13}C\{^{1}H\}$ NMR spectrum (101 MHz, acetone-d$_6$) of **[3]CF$_3$SO$_3$.**

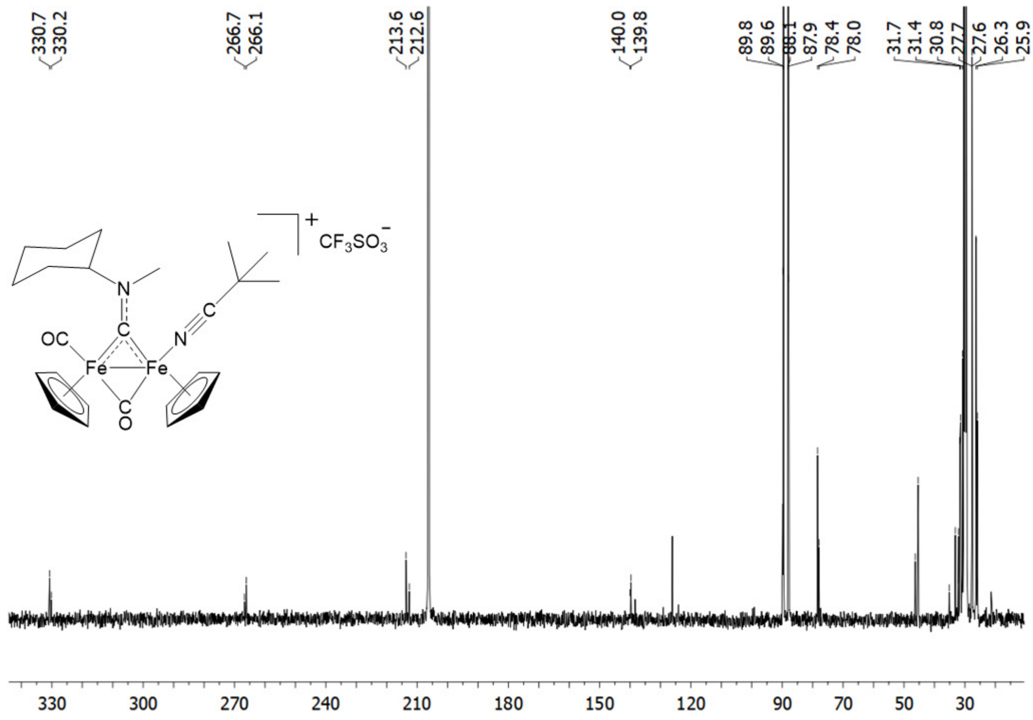

Figure S5. ^{1}H NMR spectrum (401 MHz, acetone-d$_6$) of **[4]CF$_3$SO$_3$**.

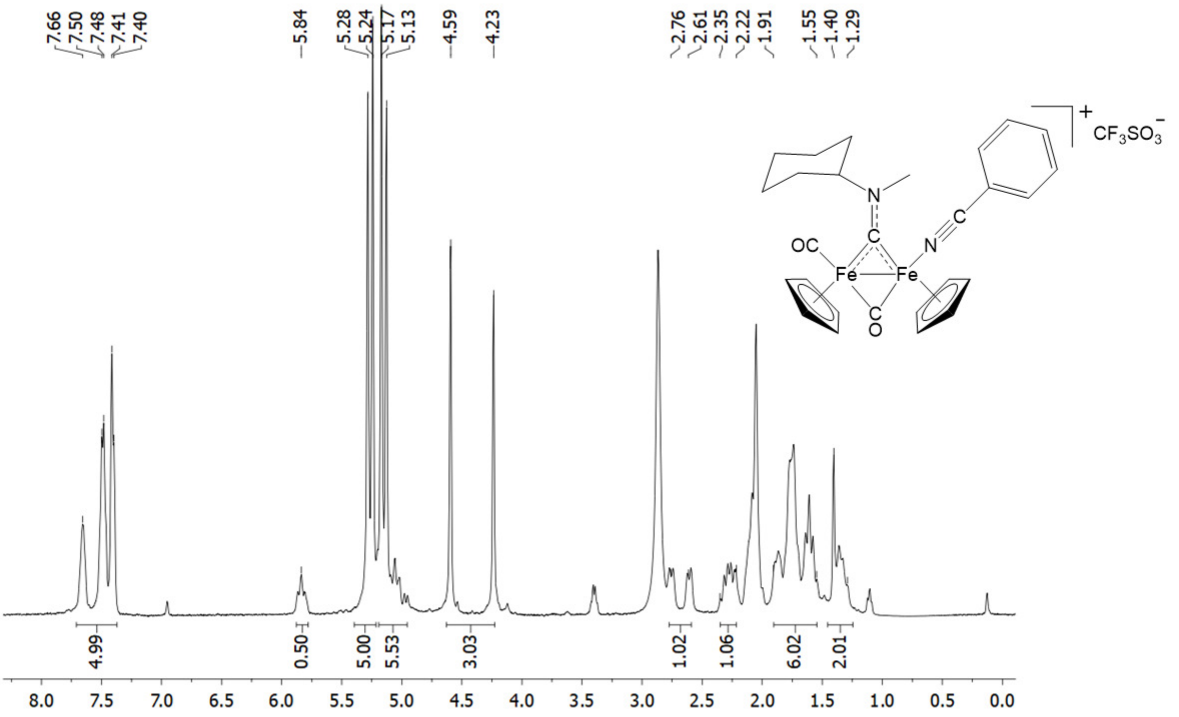

Figure S6. ^{13}C{^{1}H} NMR spectrum (101 MHz, acetone-d$_6$) of **[4]CF$_3$SO$_3$.**

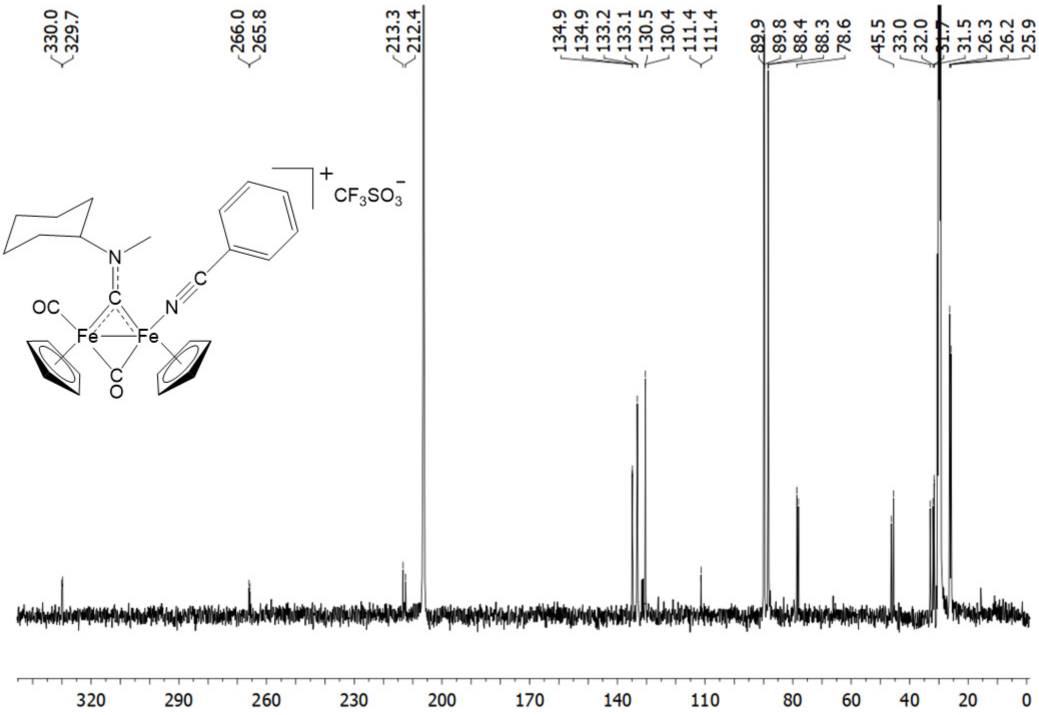

Figure S7. ^{1}H NMR spectrum (401 MHz, acetone-d$_6$) of **[5]CF$_3$SO$_3$**.

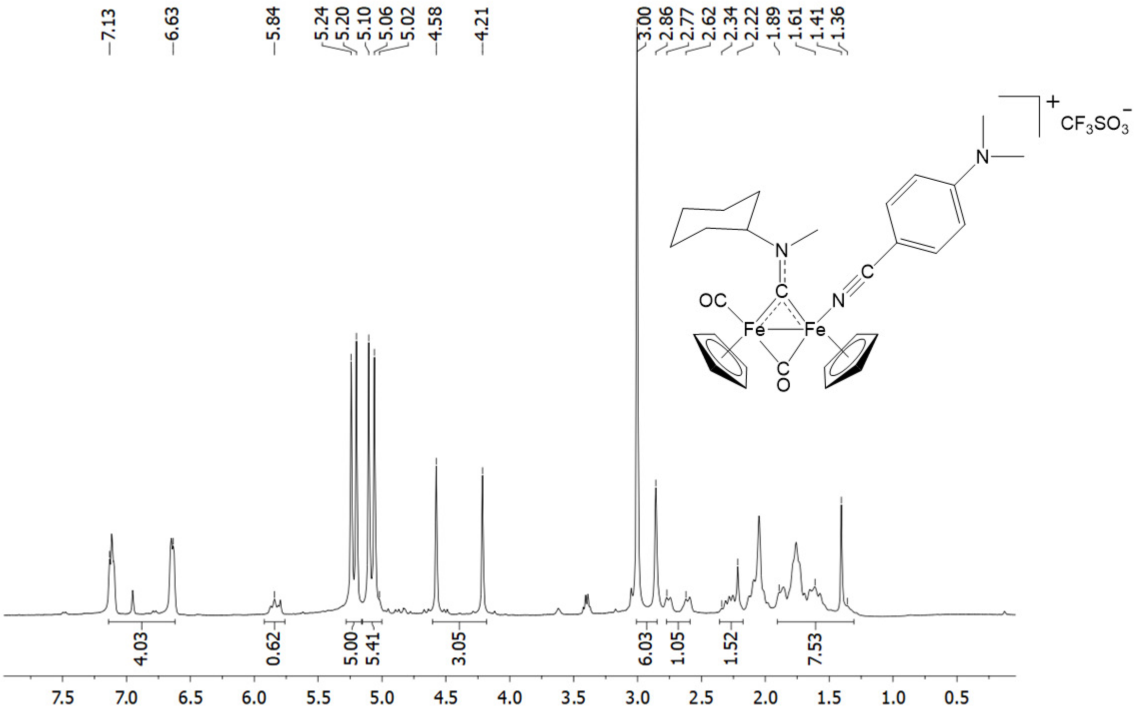

Figure S8. ^{13}C{^{1}H} NMR spectrum (101 MHz, acetone-d$_6$) of **[5]CF$_3$SO$_3$.**

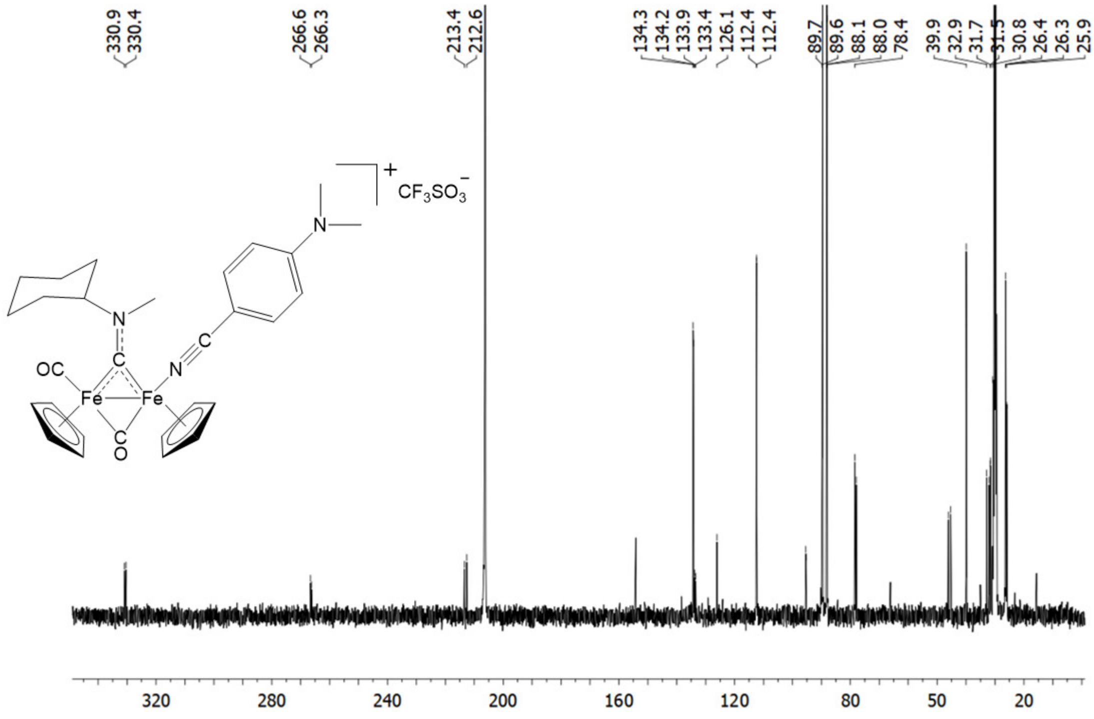

Figure S9. ^{1}H NMR spectrum (401 MHz, acetone-d$_6$) of **[6]CF$_3$SO$_3$**.

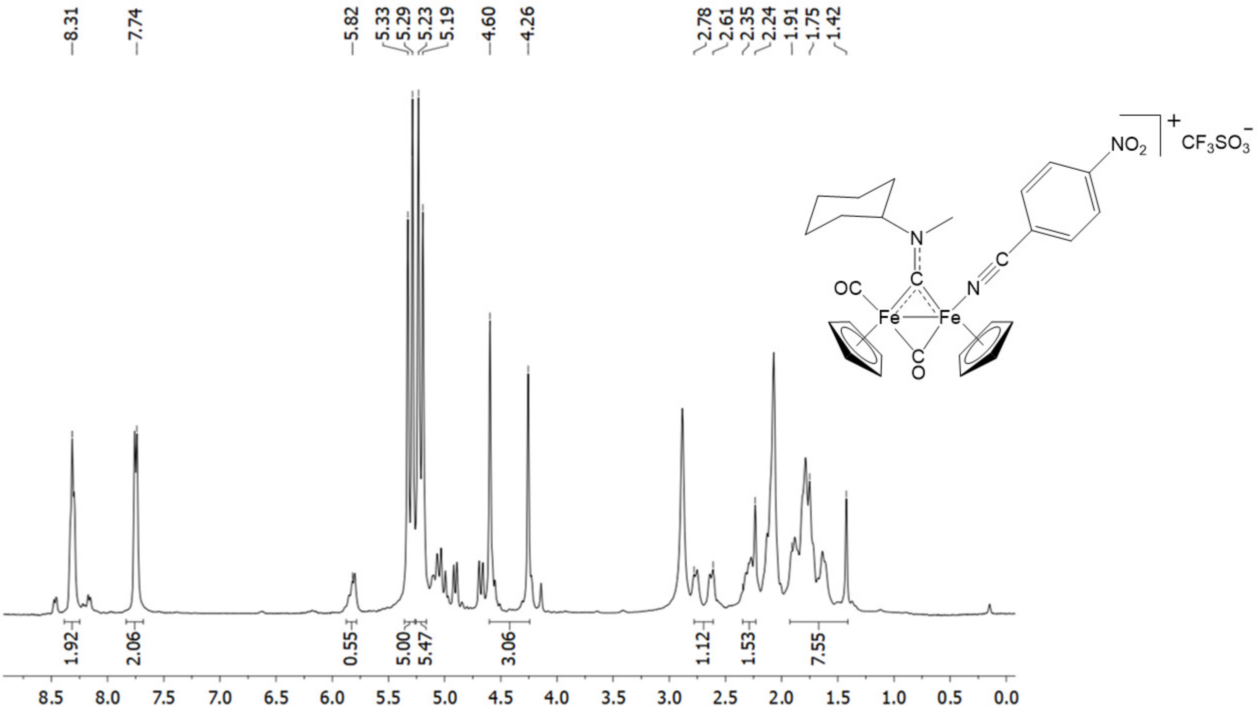

Figure S10. ^{13}C{^{1}H} NMR spectrum (101 MHz, acetone-d$_6$) of **[6]CF$_3$SO$_3$**.

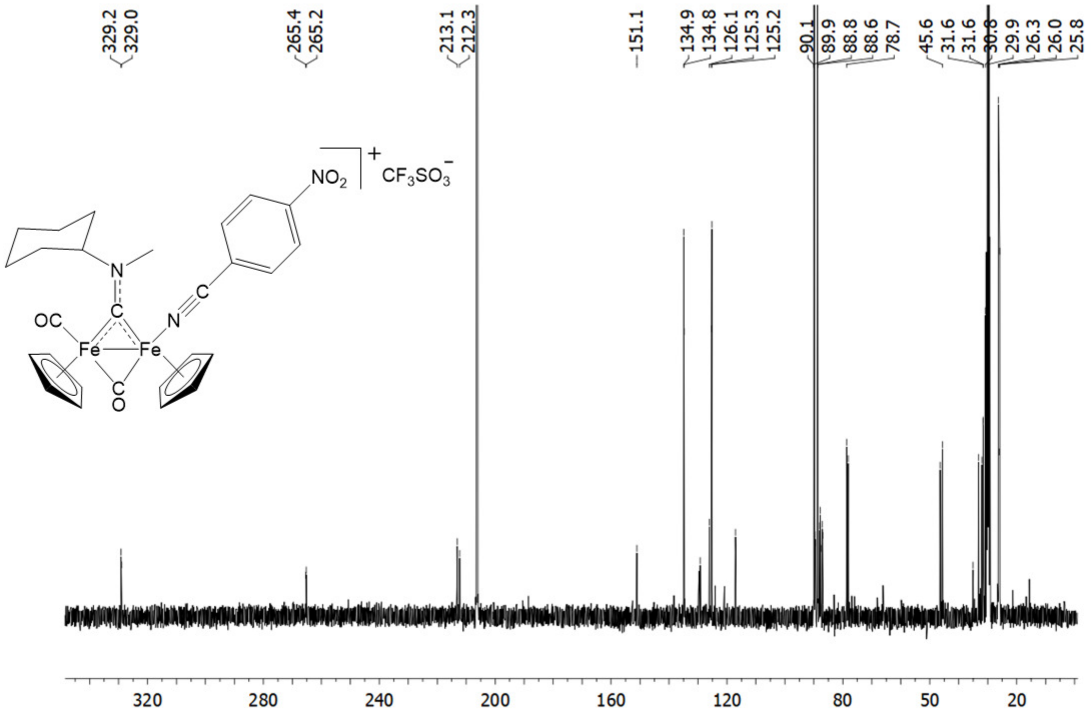

Figure S11. ^{1}H NMR spectrum (401 MHz, acetone-d$_6$) of **[7]CF$_3$SO$_3$**.

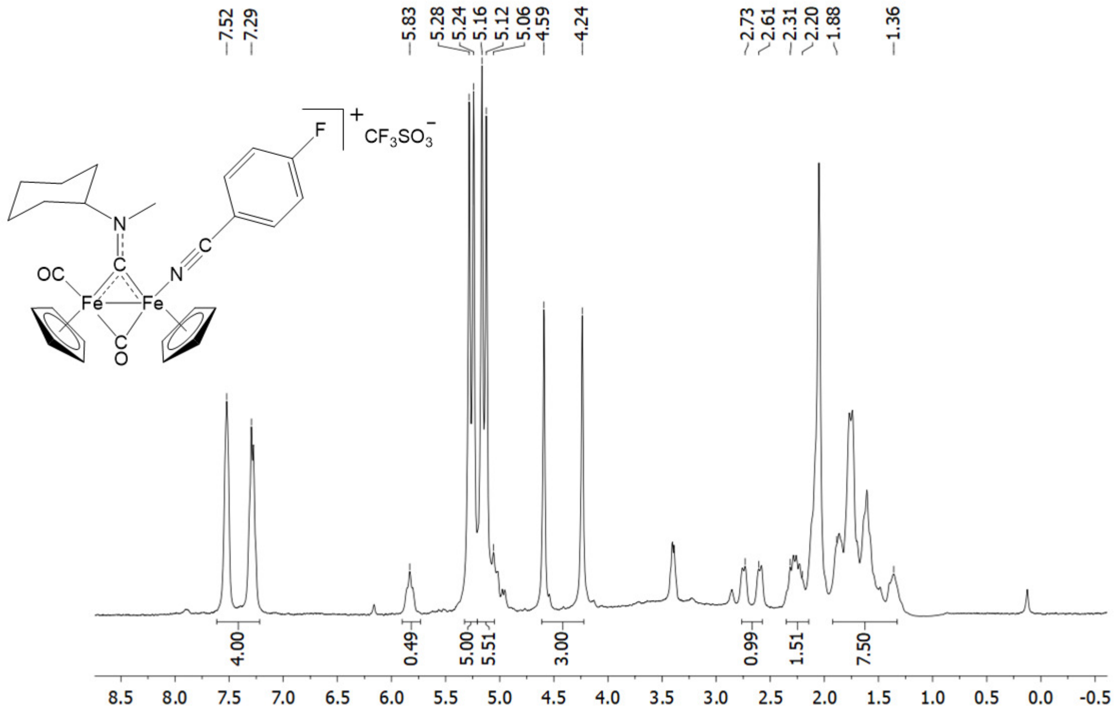

Figure S12. ^{13}C{^{1}H} NMR spectrum (101 MHz, acetone-d$_6$) of **[7]CF$_3$SO$_3$**.

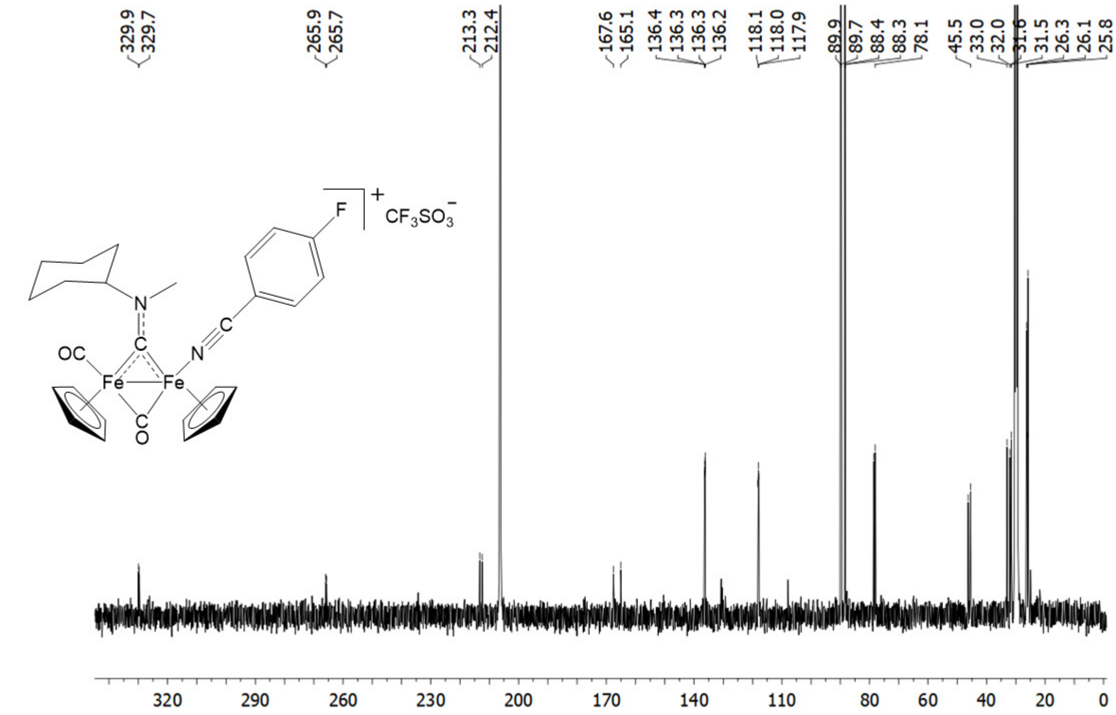

Figure S13. ^{19}F NMR spectrum (101 MHz, acetone-d$_6$) of **[7]CF$_3$SO$_3$**.

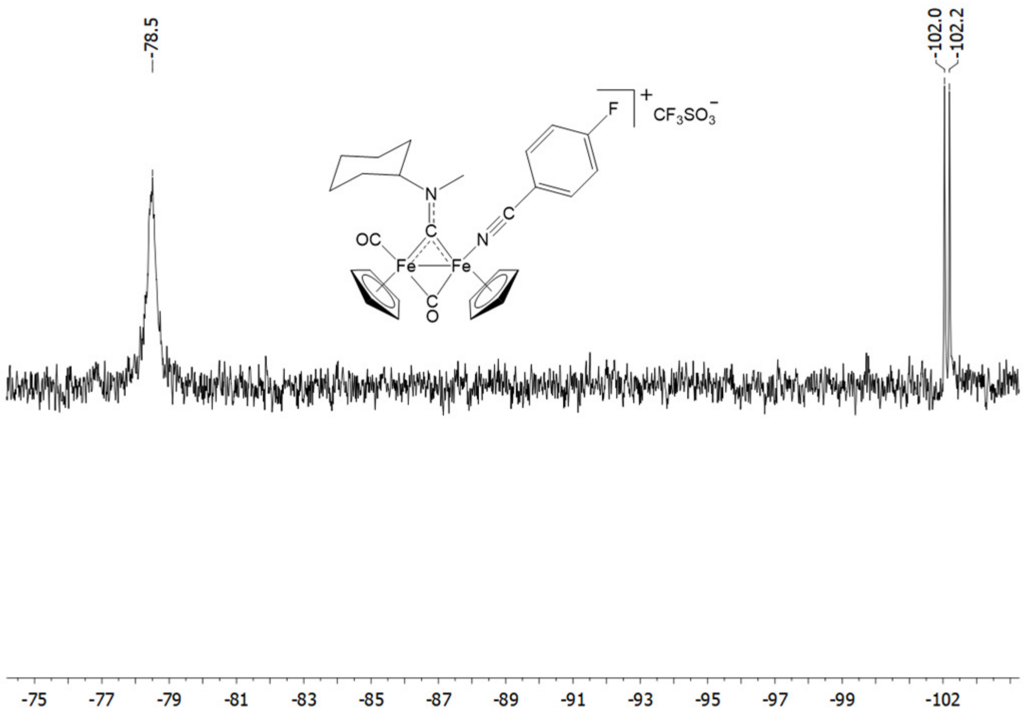

Figure S14. ^{1}H NMR spectrum (401 MHz, acetone-d_6) of **[8]CF_3SO_3**.

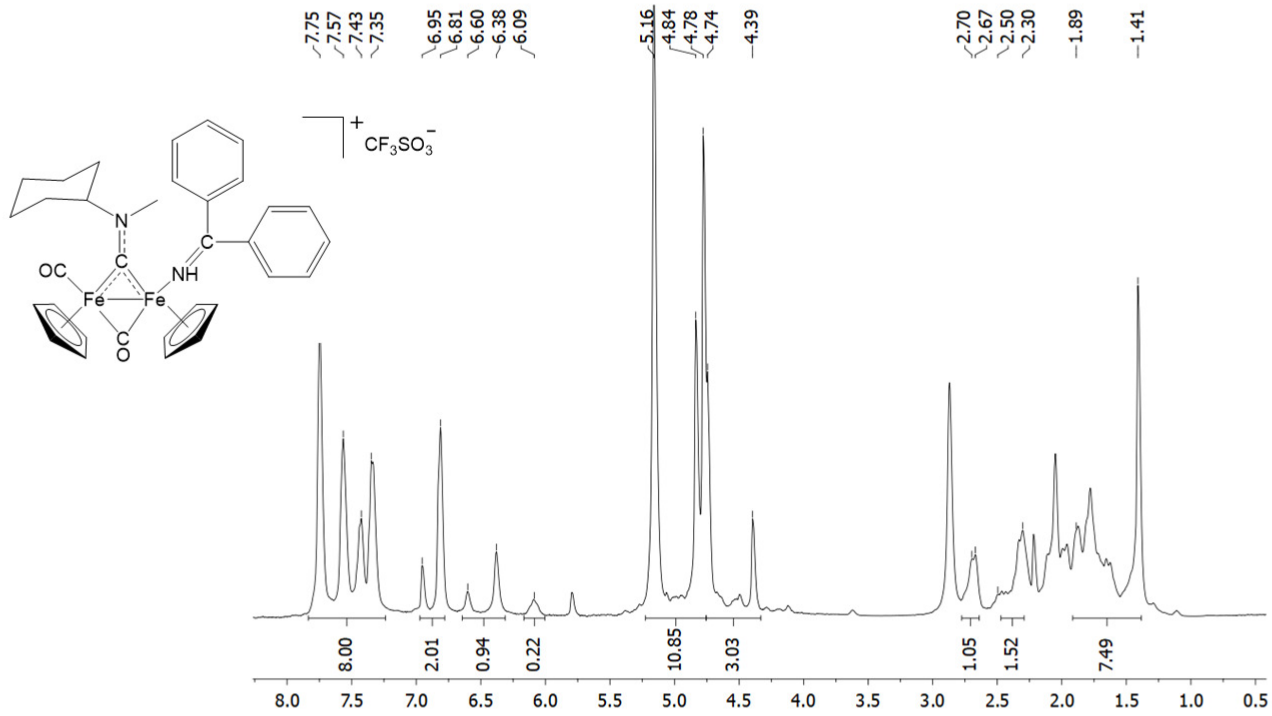

Figure S15. $^{13}C\{^{1}H\}$ NMR spectrum (101 MHz, acetone-d_6) of **[8]CF_3SO_3**.

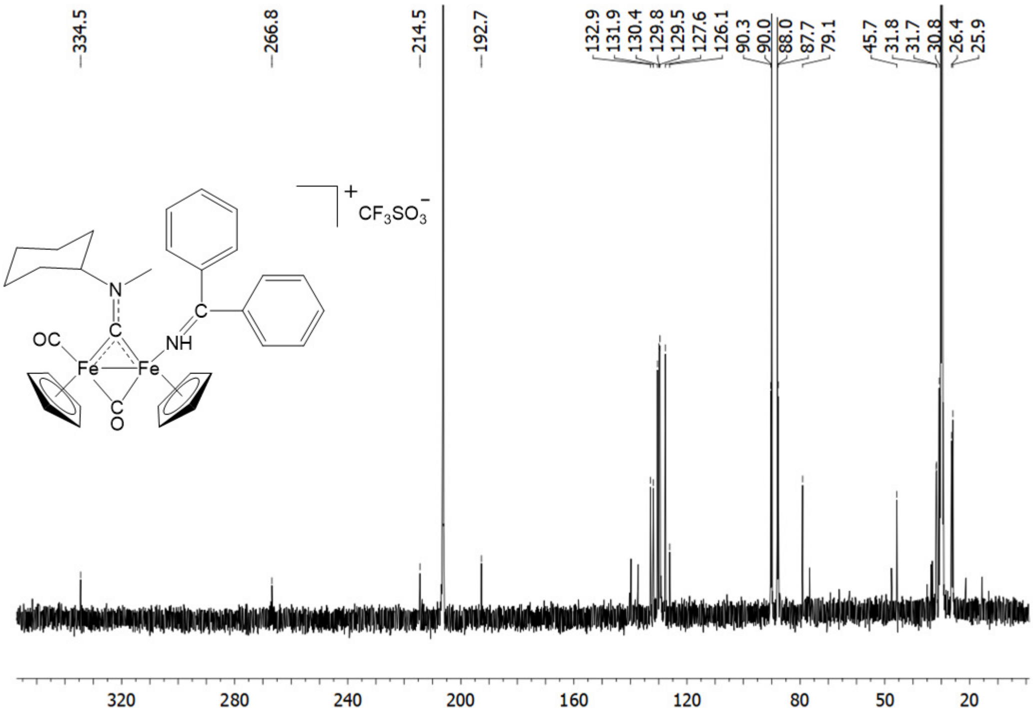

Figure S18. ^{1}H NMR spectrum (401 MHz, acetone-d_6) of **[9]CF₃SO₃**.

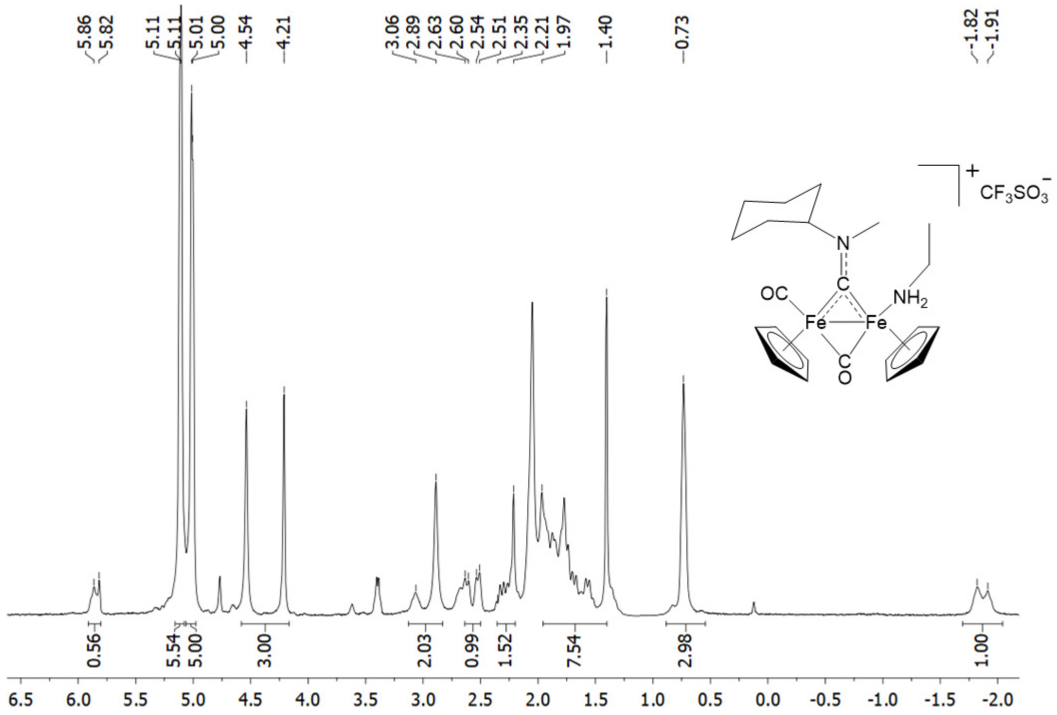

Figure S19. $^{13}C\{^{1}H\}$ NMR spectrum (101 MHz, acetone-d_6) of **[9]CF₃SO₃**.

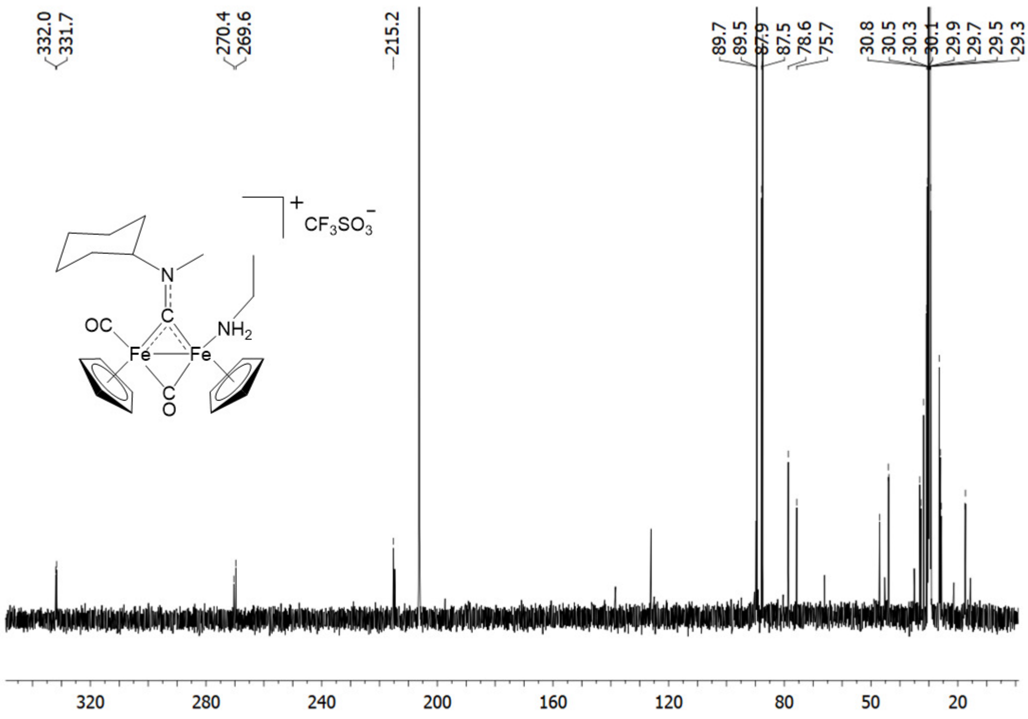

Figure S20. ^{1}H NMR spectrum (401 MHz, acetone-d$_6$) of **[10]CF$_3$SO$_3$**.

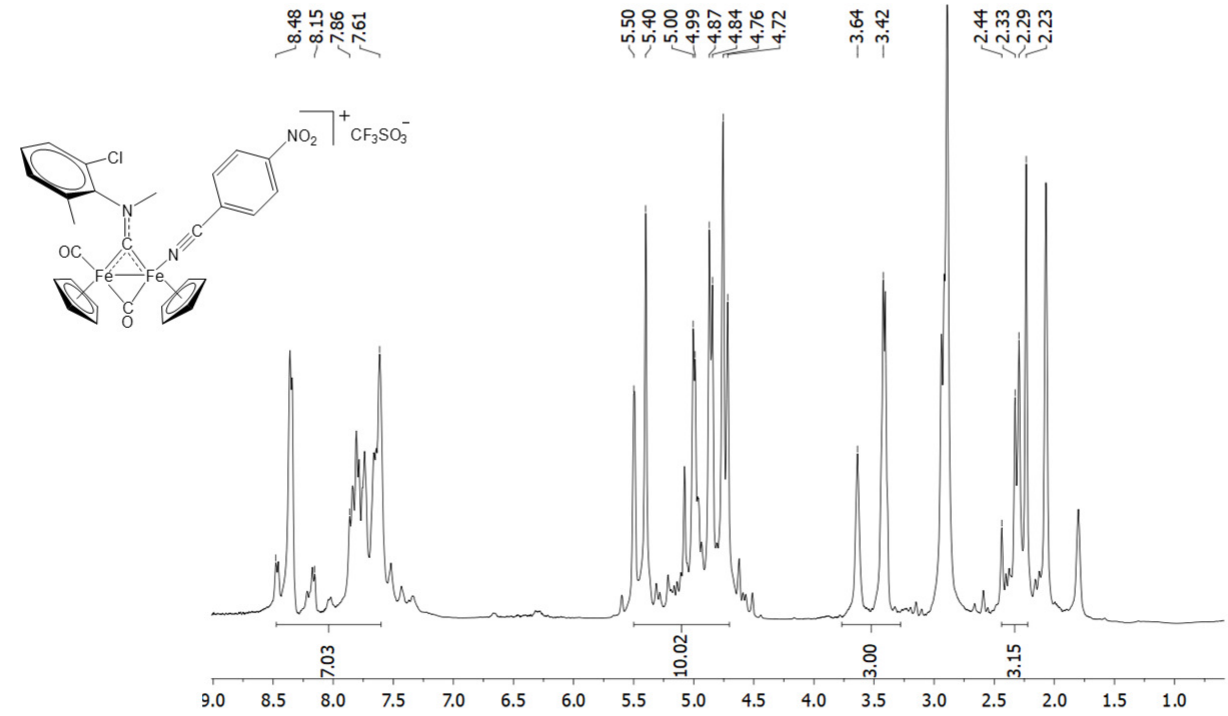

Figure S21. $^{13}C\{^{1}H\}$ NMR spectrum (101 MHz, acetone-d$_6$) of **[10]CF$_3$SO$_3$**.

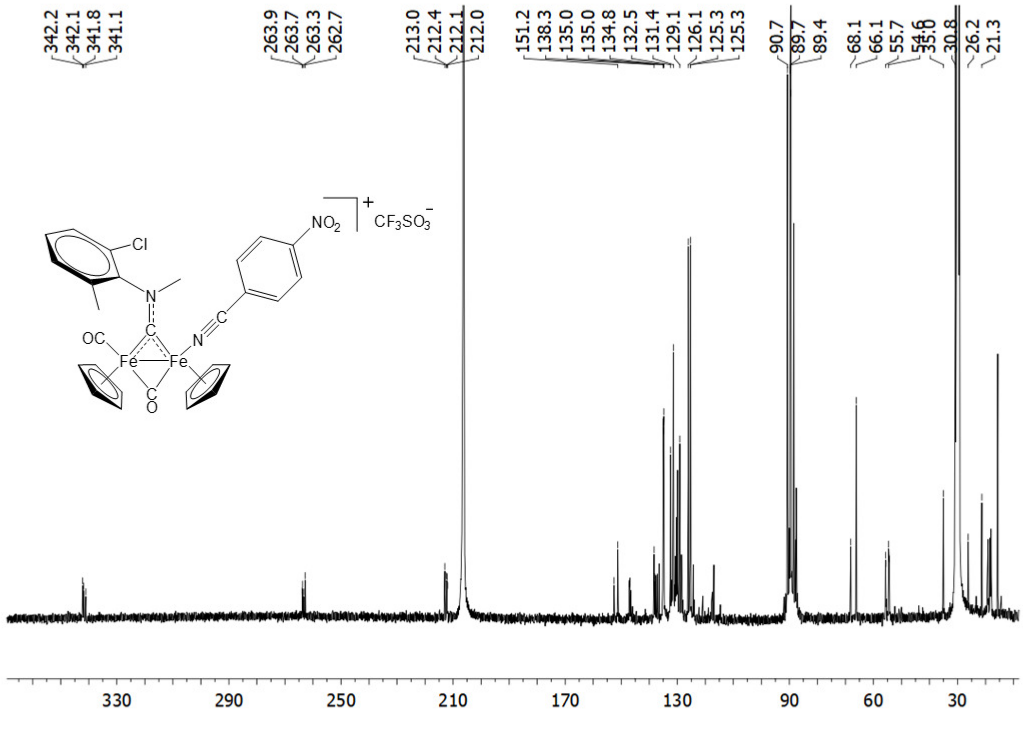

Figure S22. ^{1}H NMR spectrum (401 MHz, acetone-d$_6$) of **[11]CF$_3$SO$_3$**.

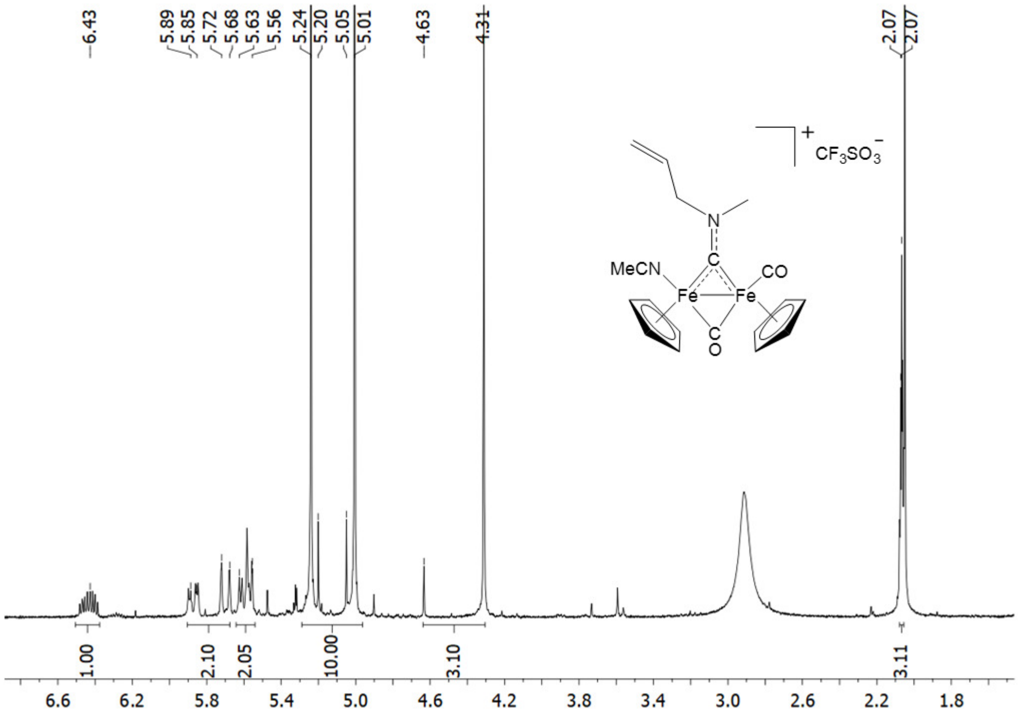

Figure S23. ^{13}C{^{1}H} NMR spectrum (101 MHz, acetone-d$_6$) of **[11]CF$_3$SO$_3$**.

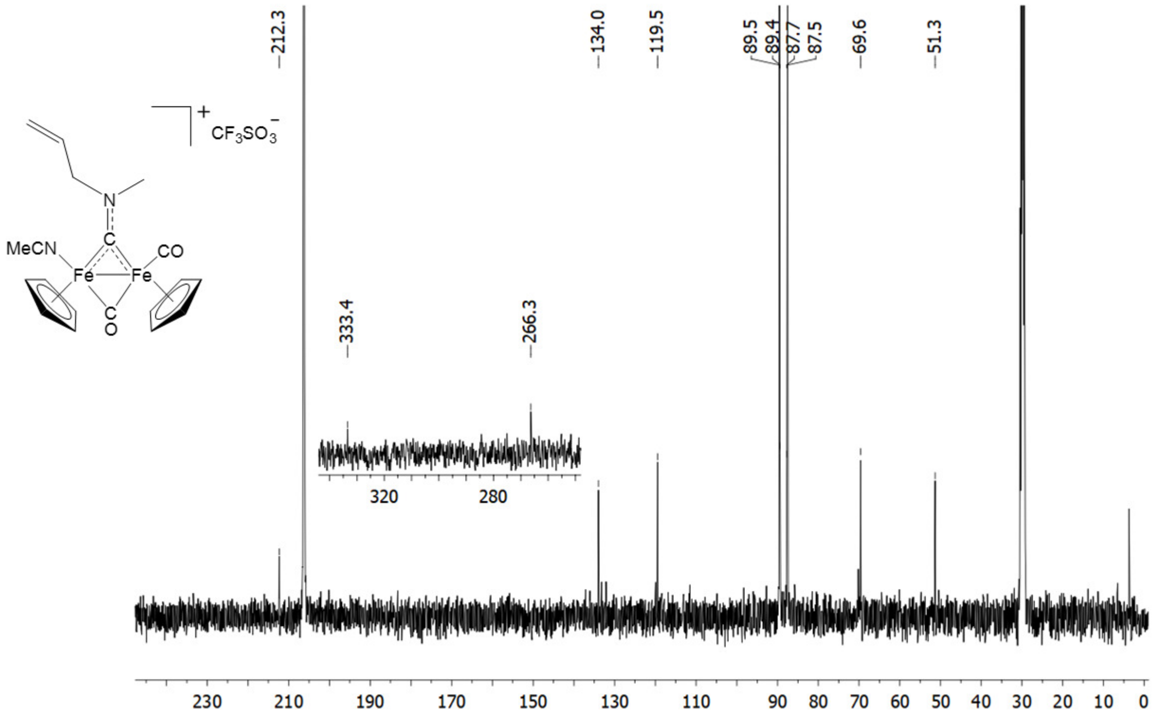

Figure S24. ^{1}H NMR spectrum (401 MHz, acetone-d$_6$) of **[12]CF$_3$SO$_3$**.

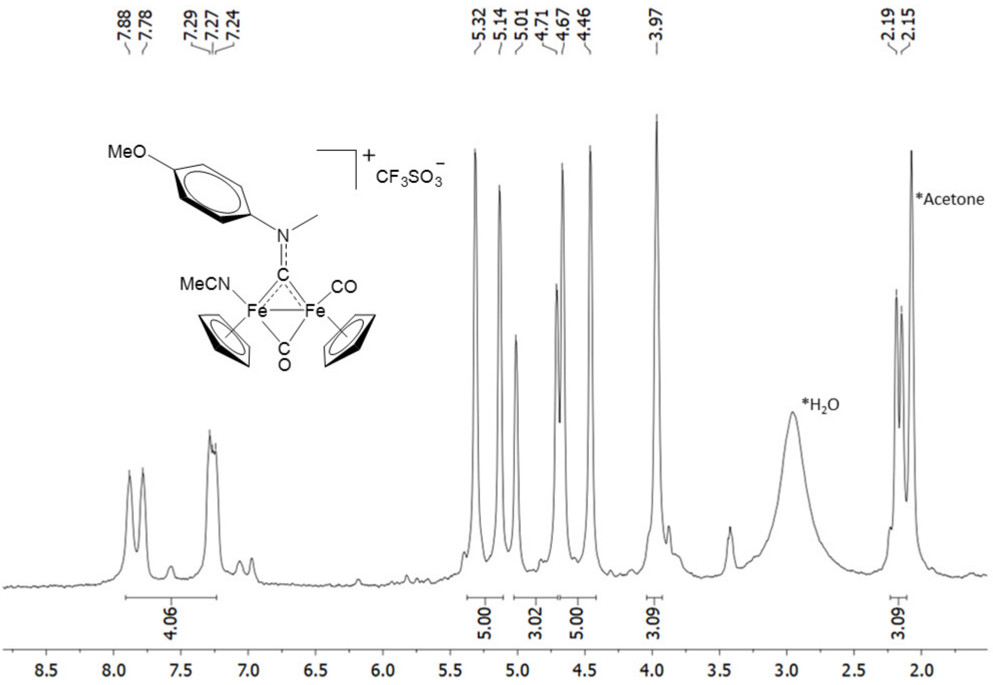

Figure S25. $^{13}C\{^{1}H\}$ NMR spectrum (101 MHz, acetone-d$_6$) of **[12]CF$_3$SO$_3$**.

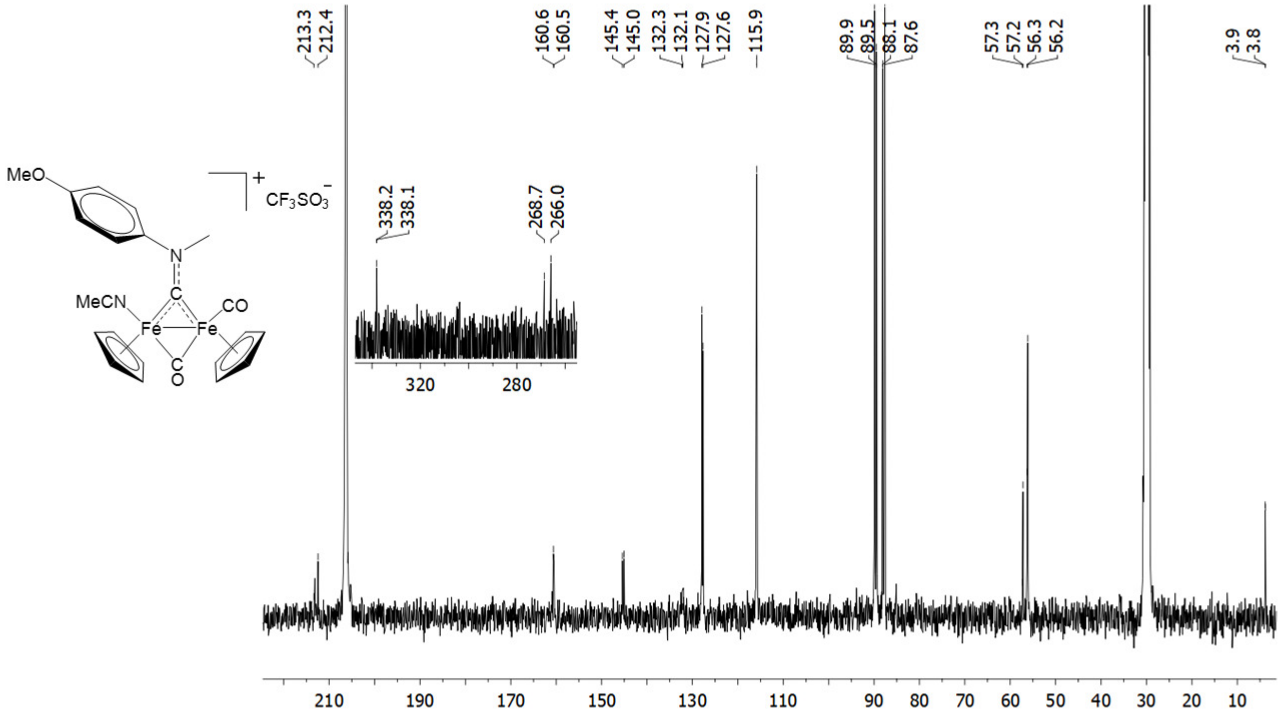

Figure S26. Solid state IR spectrum of **[2]CF$_3$SO$_3$**.

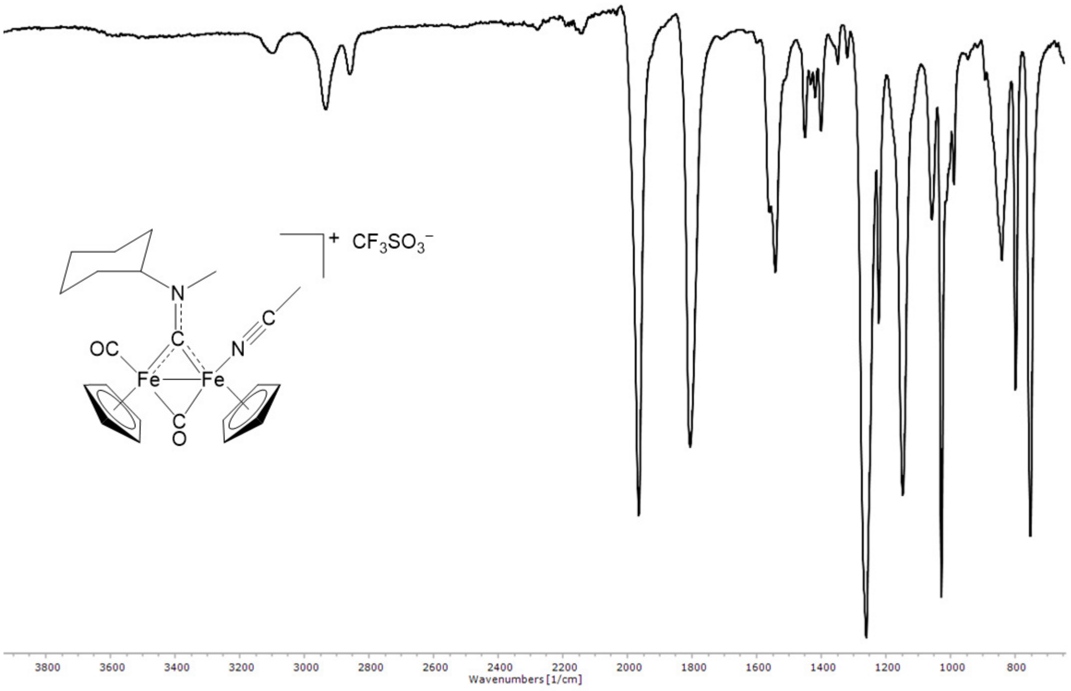

Figure S27. Solution (CH$_2$Cl$_2$) IR spectrum of **[2]CF$_3$SO$_3$**.

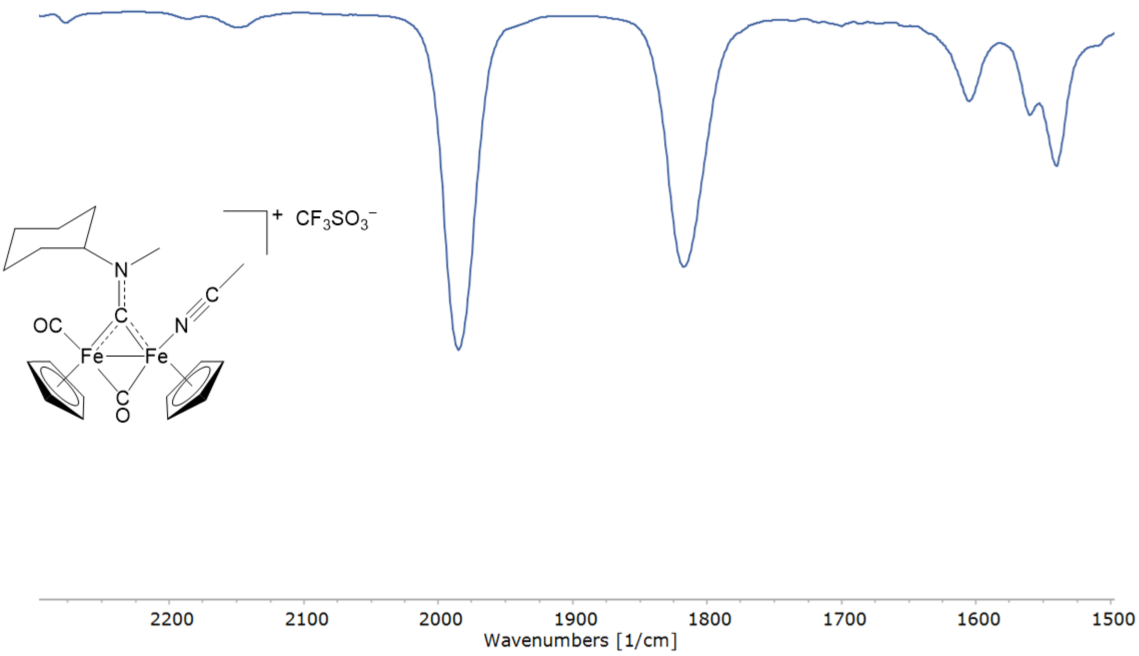

Figure S28. Solid state IR spectrum of **[3]CF$_3$SO$_3$**.

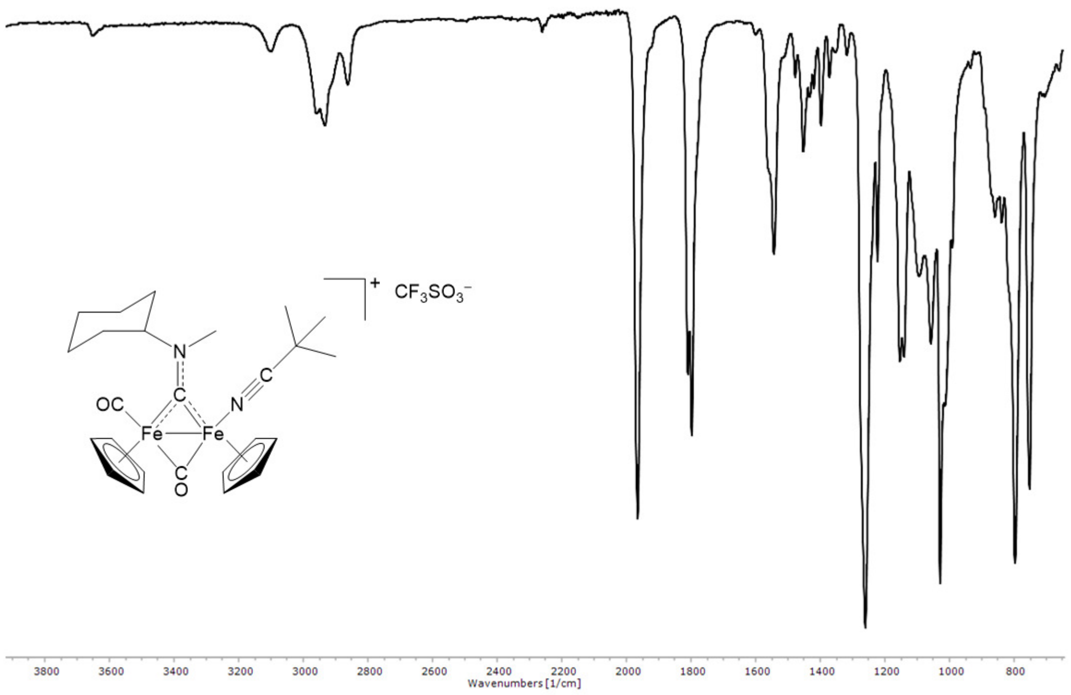

Figure S29. Solution (CH$_2$Cl$_2$) IR spectrum of **[3]CF$_3$SO$_3$**.

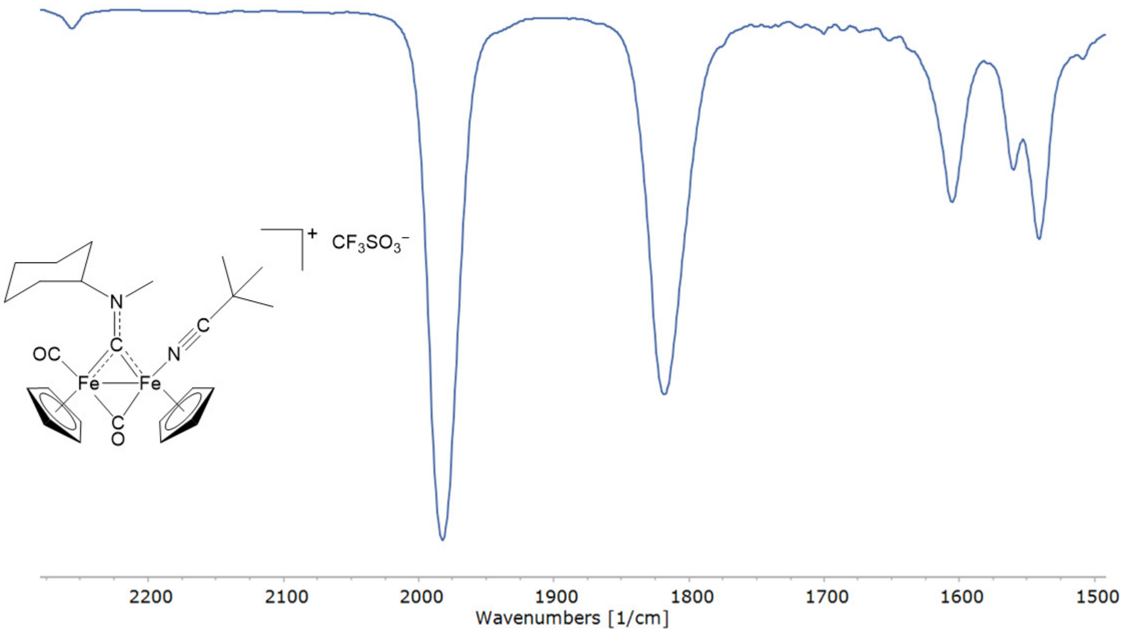

Figure S30. Solid state IR spectrum of **[4]CF_3SO_3**.

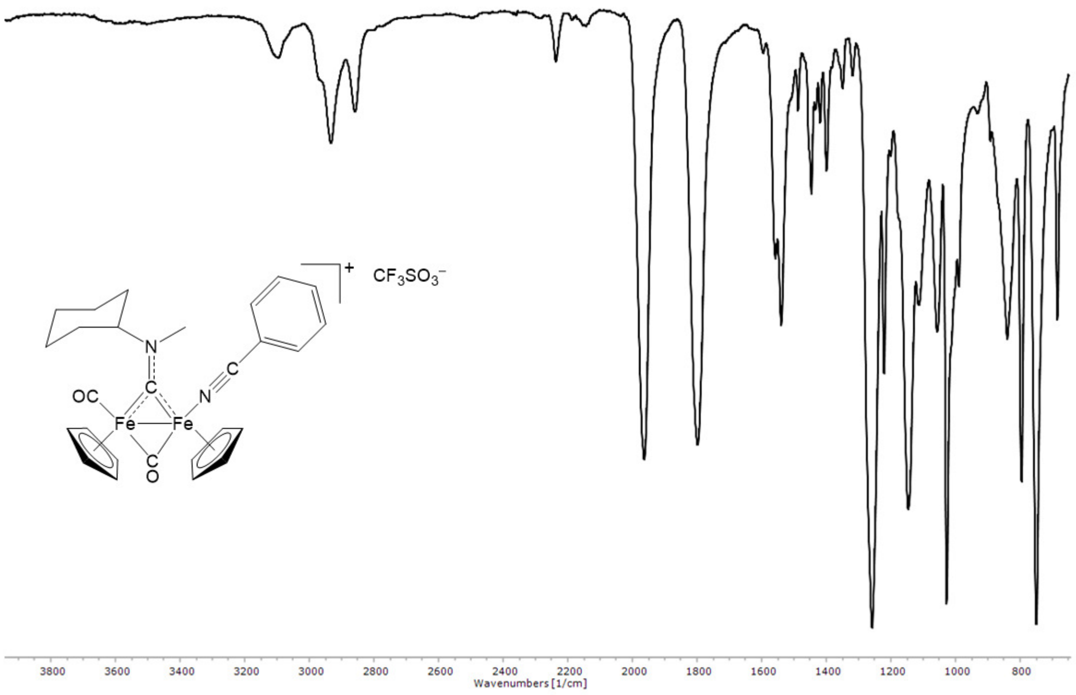

Figure S31. Solution (CH_2Cl_2) IR spectrum of **[4]CF_3SO_3**.

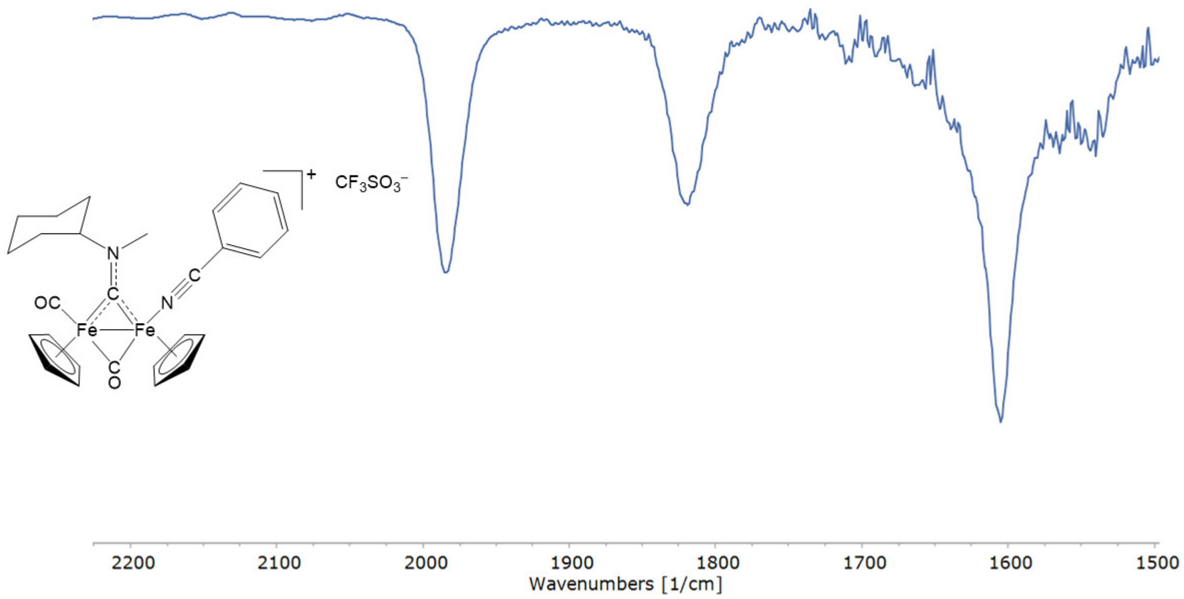

Figure S32. Solid state IR spectrum of **[5]CF$_3$SO$_3$**.

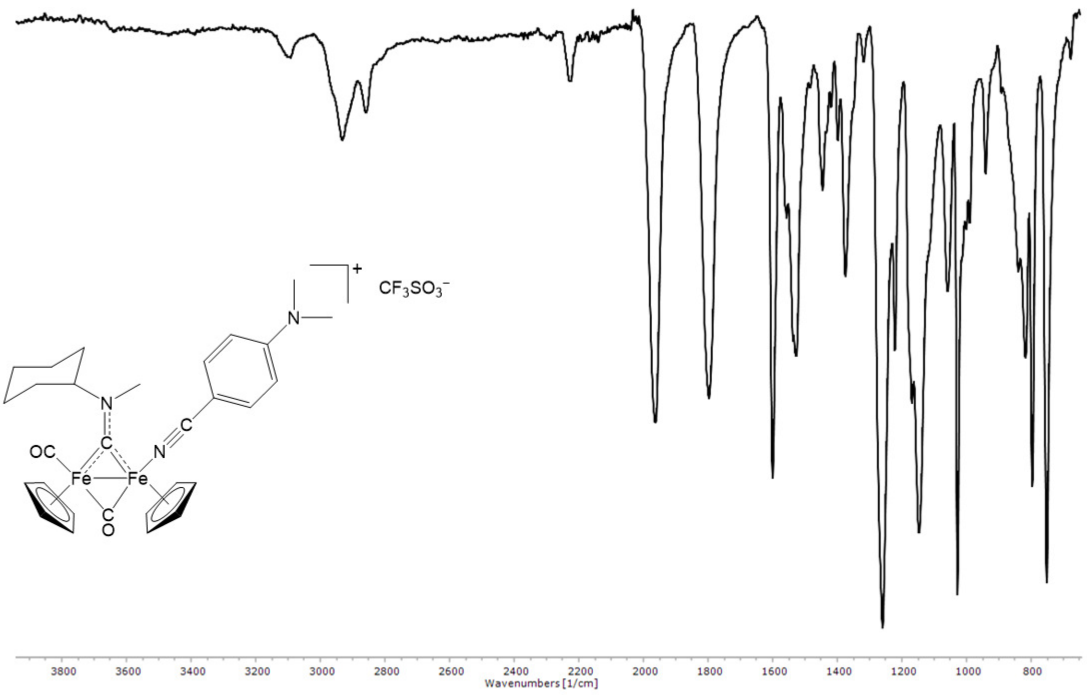

Figure S33. Solution (CH$_2$Cl$_2$) IR spectrum of **[5]CF$_3$SO$_3$**.

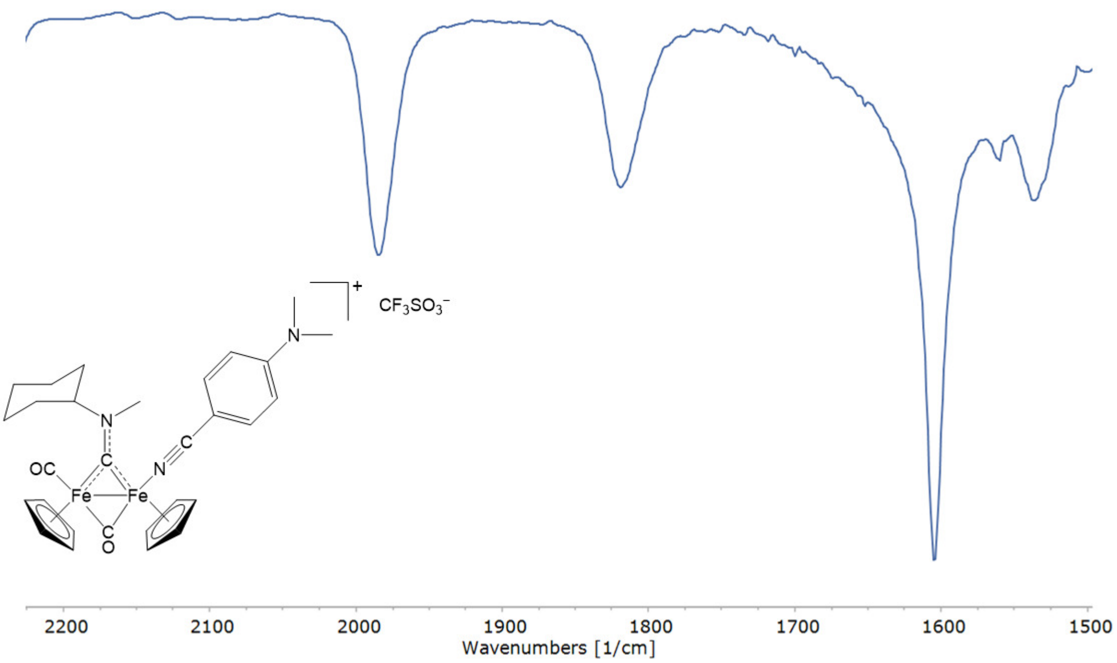

Figure S34. Solid state IR spectrum of **[6]CF$_3$SO$_3$**.

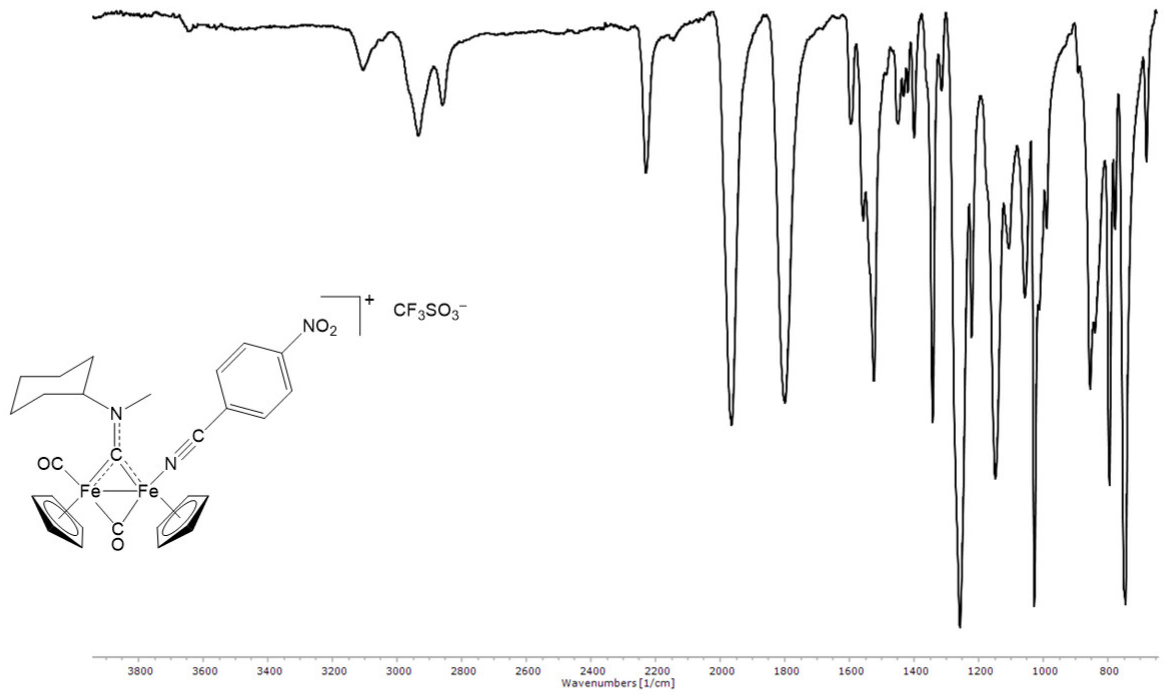

Figure S35. Solution (CH_2Cl_2) IR spectrum of **[6]CF$_3$SO$_3$**.

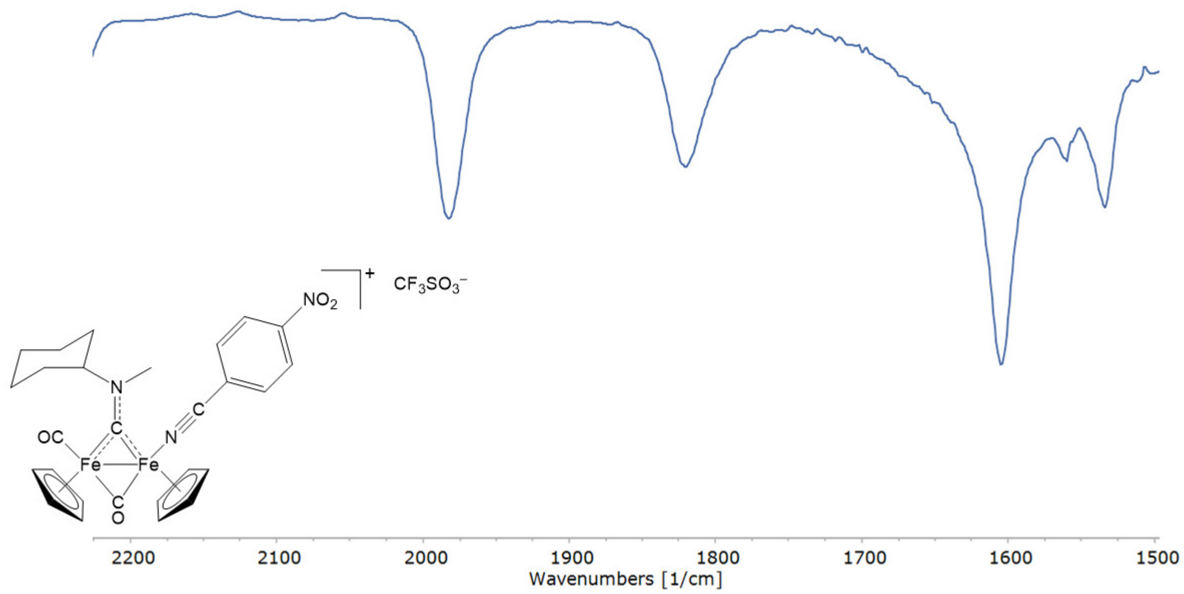

Figure S36. Solid state IR spectrum of **[7]CF₃SO₃**.

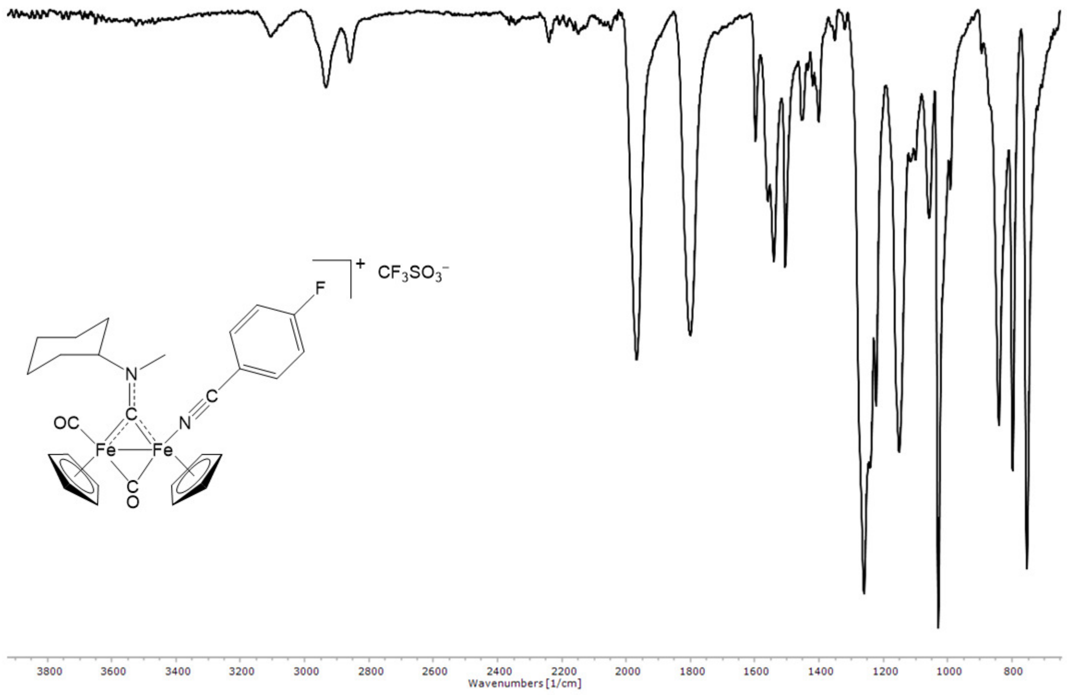

Figure S37. Solution (CH_2Cl_2) IR spectrum of **[7]CF_3SO_3**.

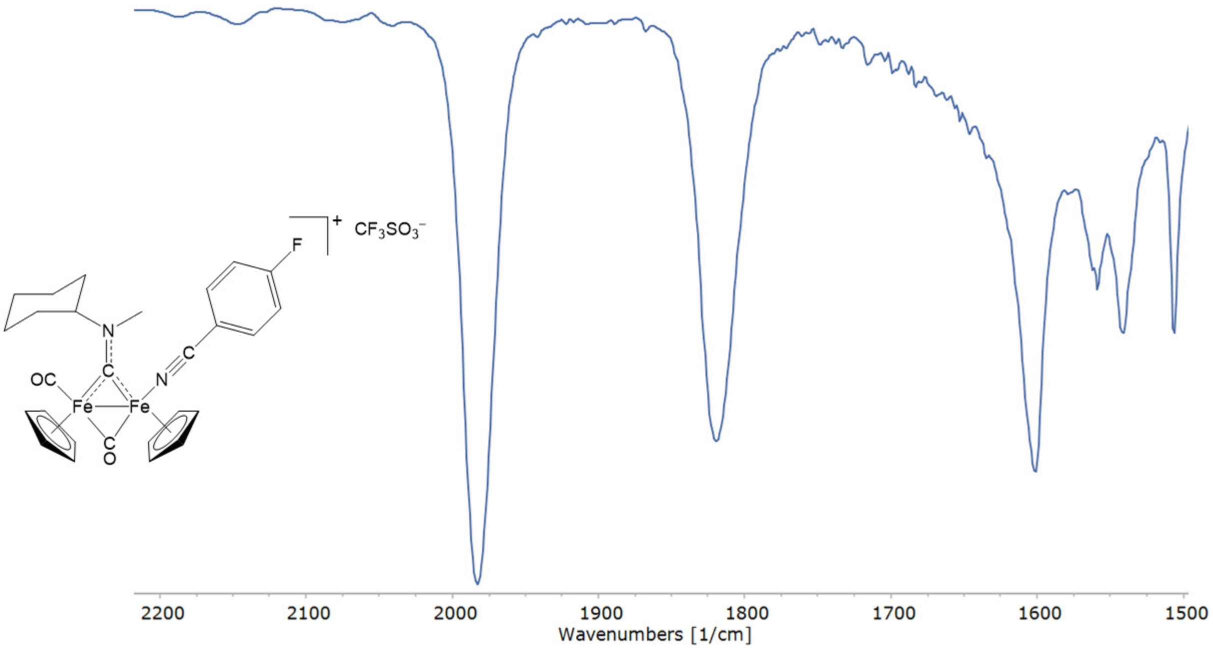

Figure S38. Solid state IR spectrum of **[8]CF₃SO₃**.

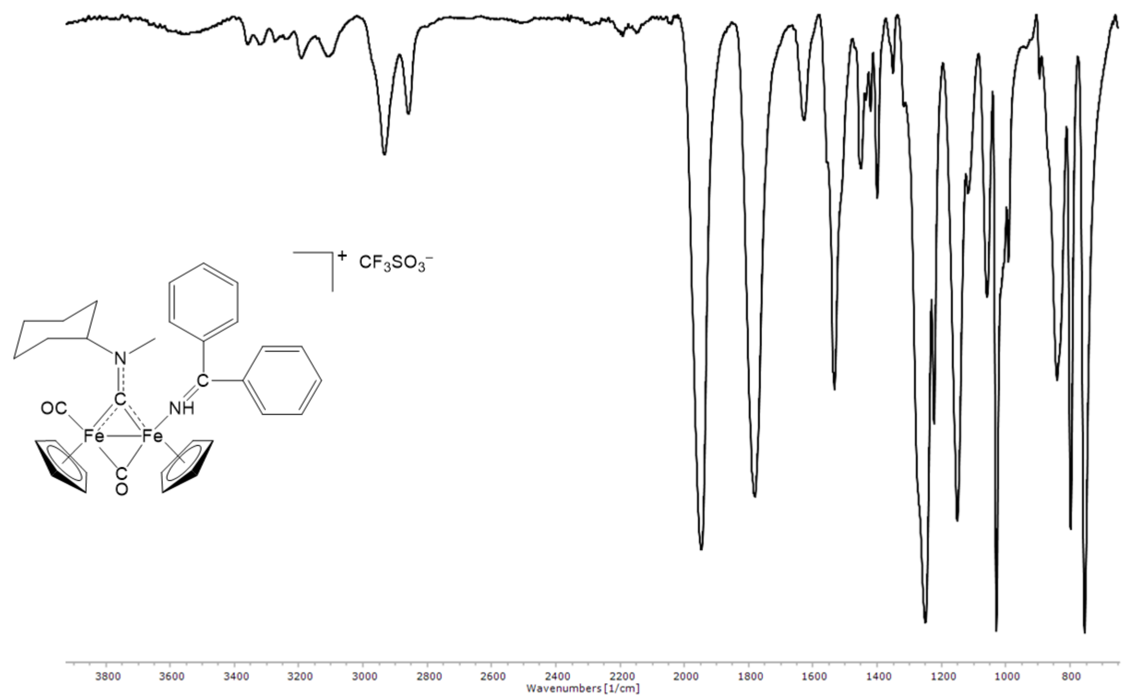

Figure S39. Solution (CH_2Cl_2) IR spectrum of **[8]CF_3SO_3**.

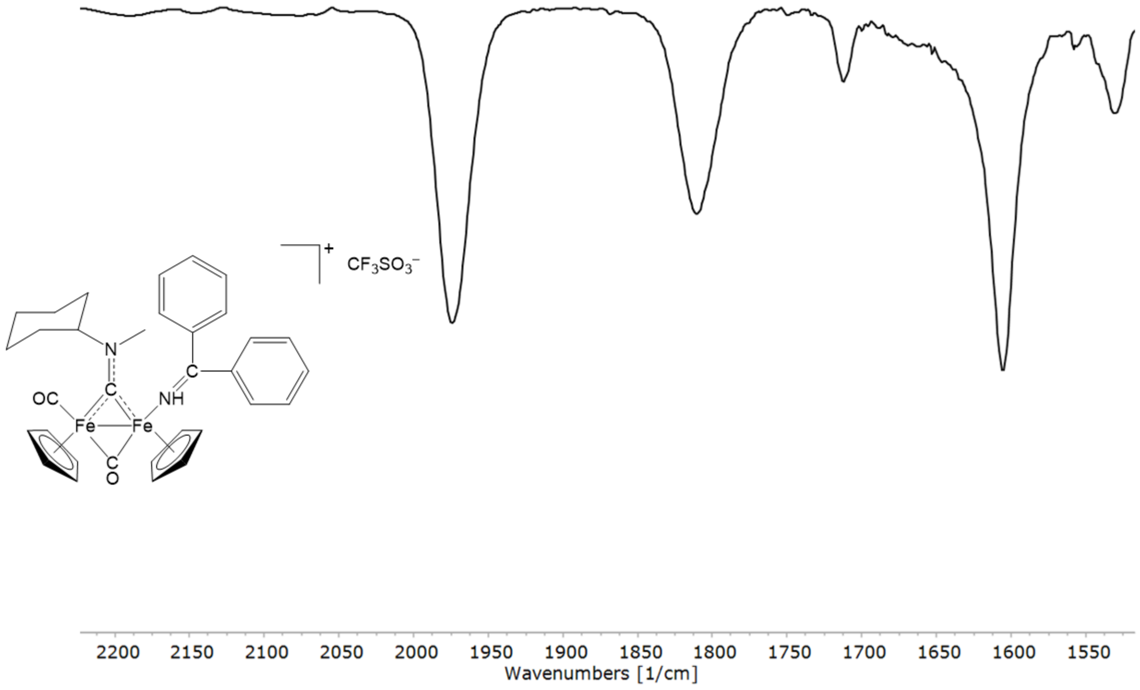

Figure S40. Solid state IR spectrum of **[9]CF_3SO_3**.

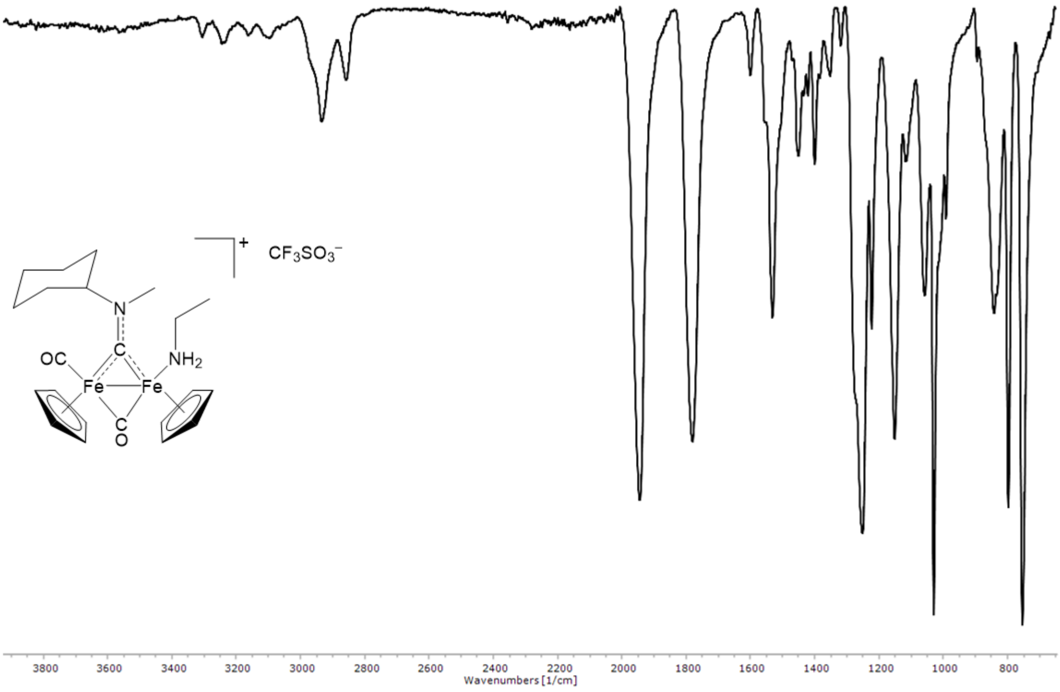

Figure S41. Solution (CH_2Cl_2) IR spectrum of **[9]CF_3SO_3**.

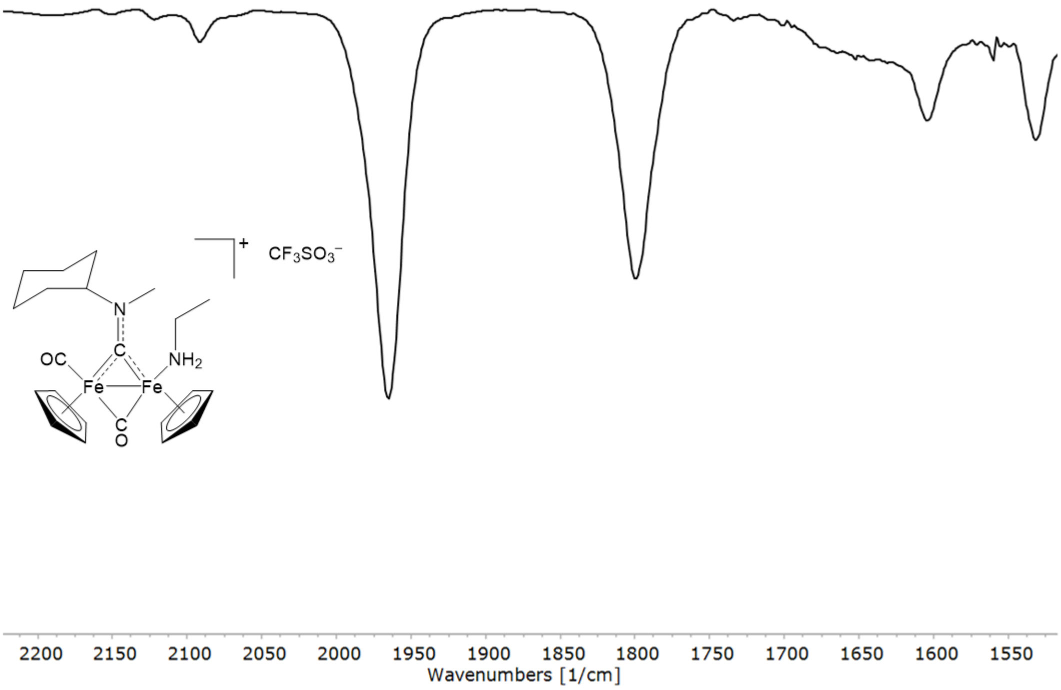

Figure S42. Solid state IR spectrum of **[10]CF$_3$SO$_3$**.

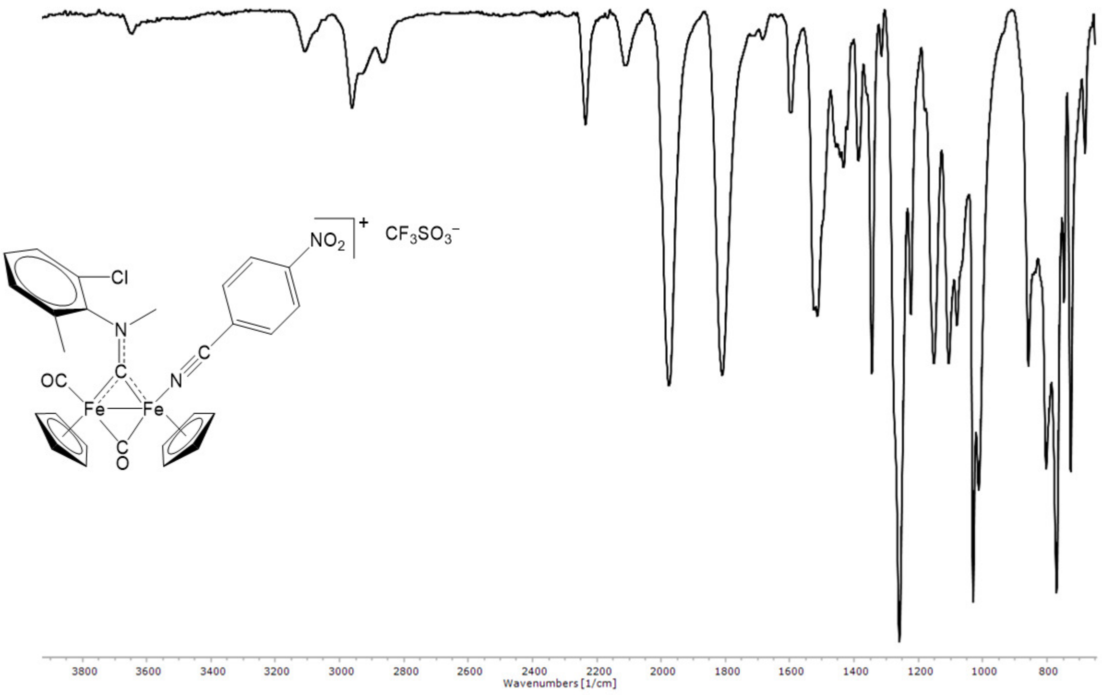

Figure S43. Solution (CH$_2$Cl$_2$) IR spectrum of **[10]CF$_3$SO$_3$**.

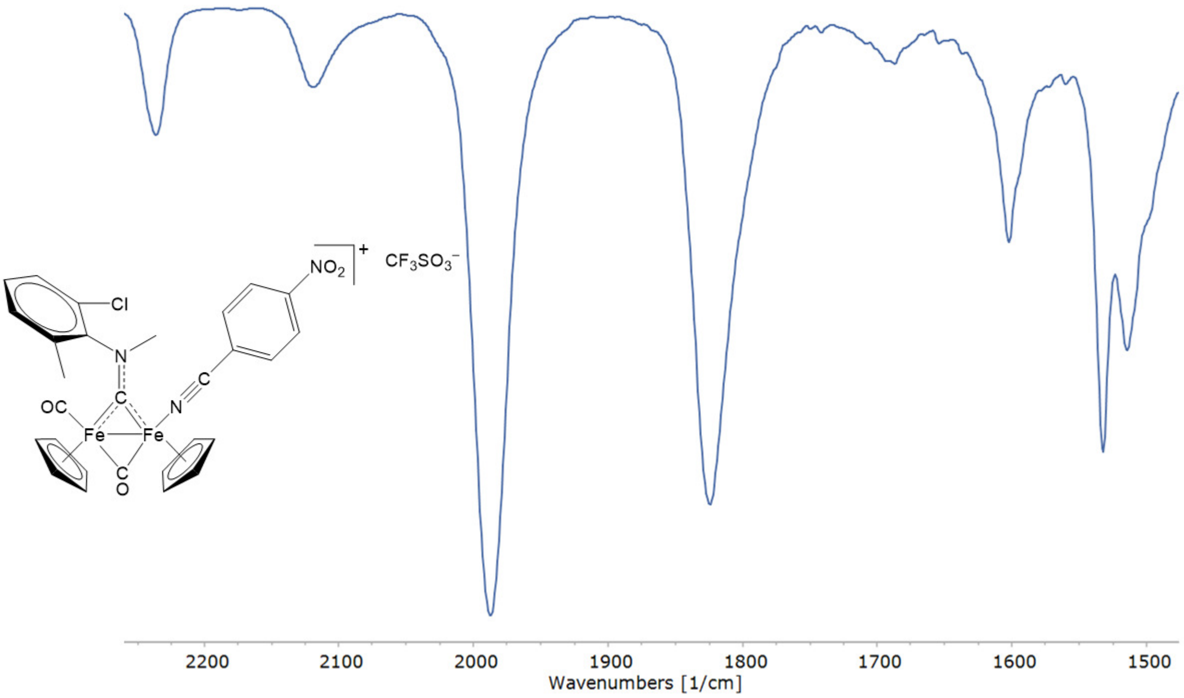

Figure S44. Solid state IR spectrum of **[11]CF_3SO_3**.

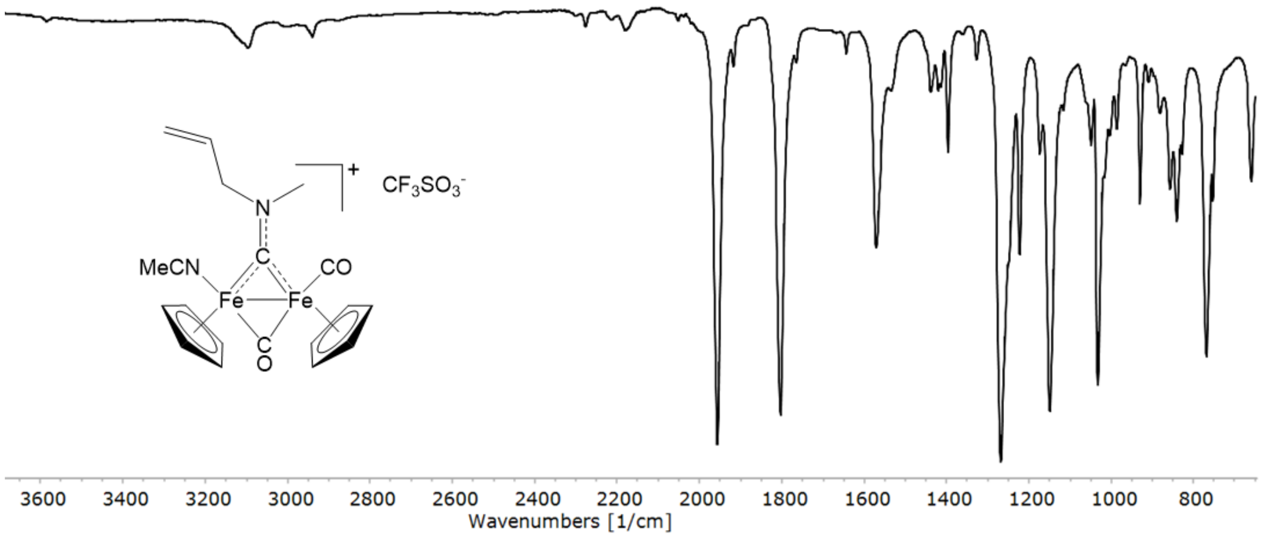

Figure S45. Solution (CH_2Cl_2) IR spectrum of **[11]CF_3SO_3**.

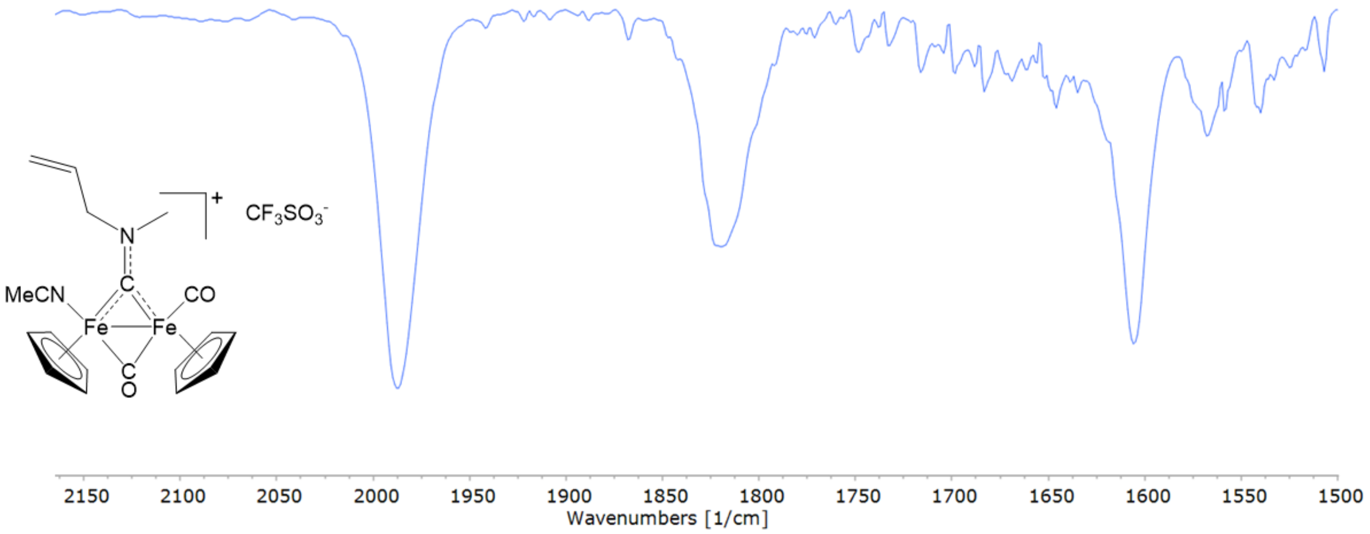

Figure S46. Solid state IR spectrum of **[12]CF$_3$SO$_3$**.

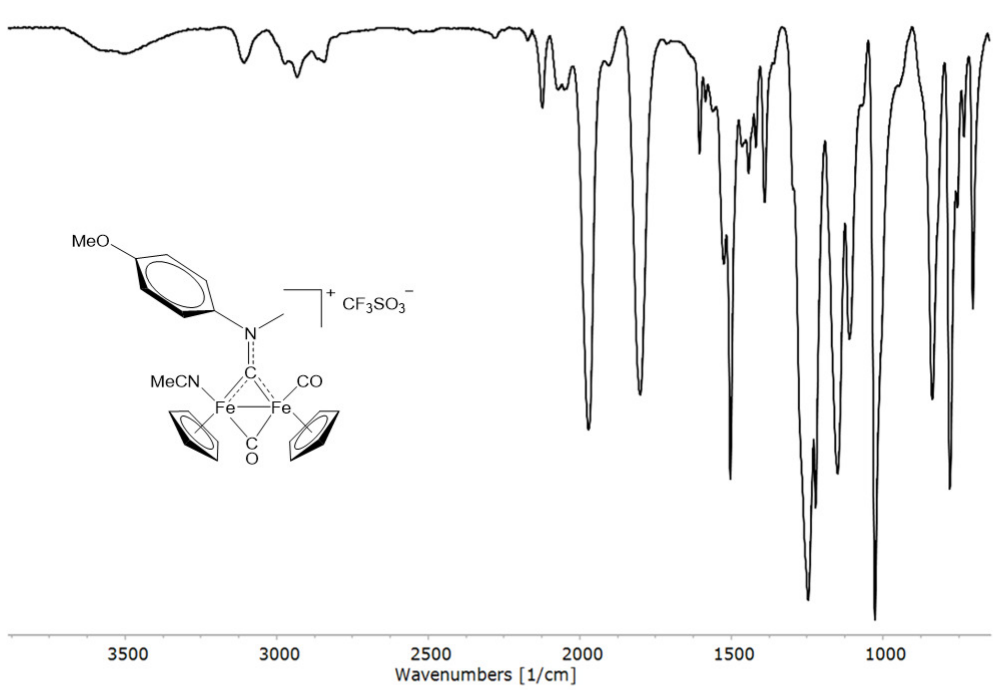

Figure S47. Solution (CH$_2$Cl$_2$) IR spectrum of **[12]CF$_3$SO$_3$**.

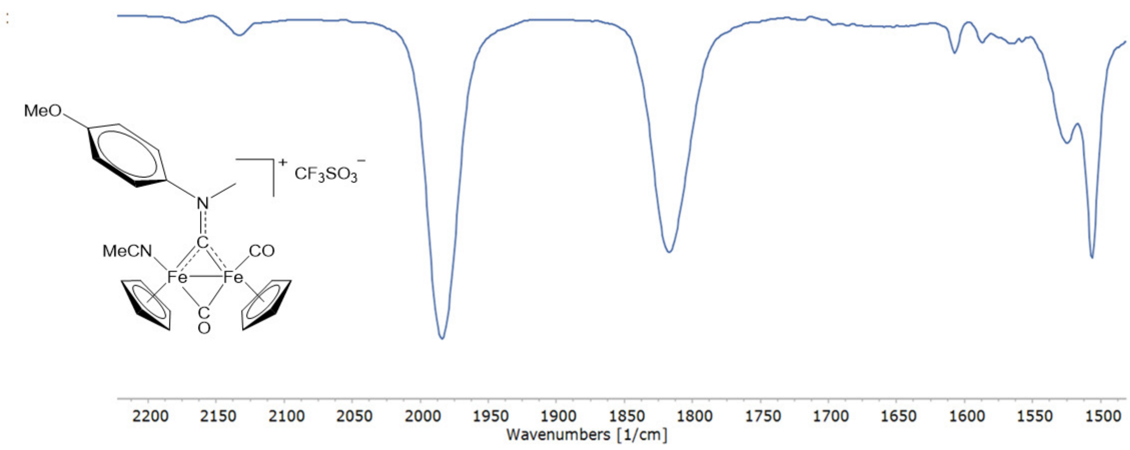

Figure S48. Isodensity surface plots (isodensity value 8 me a.u.$^{-3}$) for the deformation maps relative to $\Delta\rho_k$ of the $[Fe_2Cp_2(CO)(CNMe_2)(NCMe)]^+\cdots[CO]$ interaction (complex [**M1**]$^+$). The charge flux is red → blue. E_{int} = -65.7 kcal/mol; E_{orb} = -113.2 kca/mol; E_{st} = 55.4; E_{disp} = -7.9 kcal/mol.

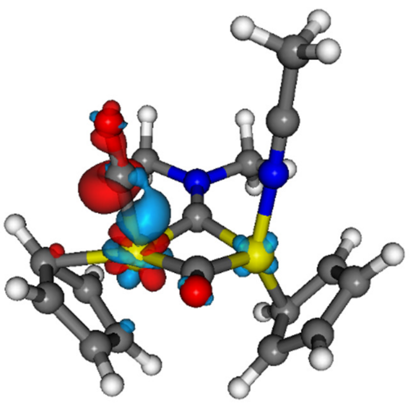

$\Delta\rho_0$ (E_0 = -49.2 kcal/mol)

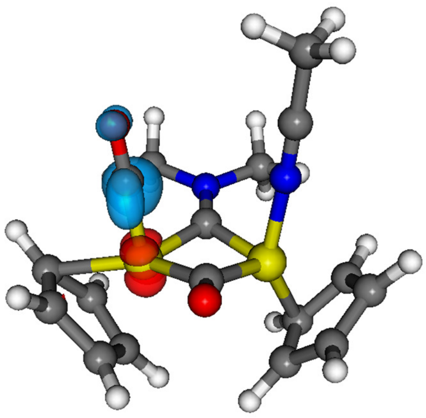

$\Delta\rho_1$ (E_1 = -31.6 kcal/mol)

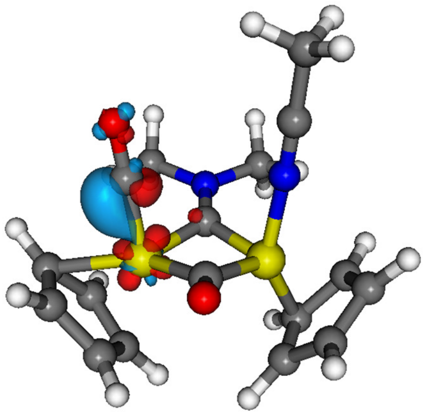

$\Delta\rho_2$ (E_2 = -24.5 kcal/mol)

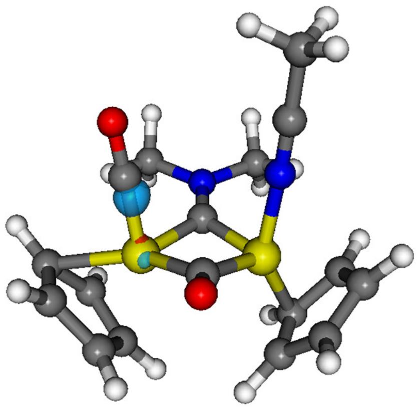

$\Delta\rho_3$ (E_3 = -5.3 kcal/mol)

Figure S49. Views of the DFT optimized structures of experimental complexes. H atoms have been omitted for clarity.

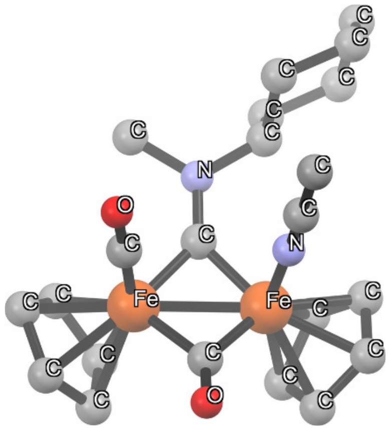

[2Z]$^+$

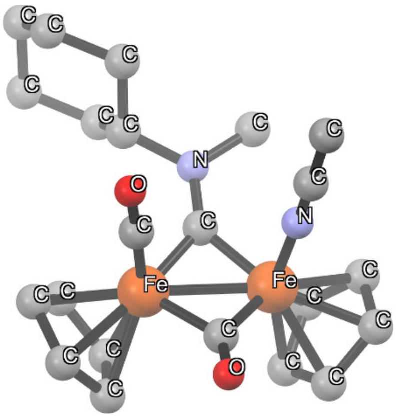

[2E]$^+$

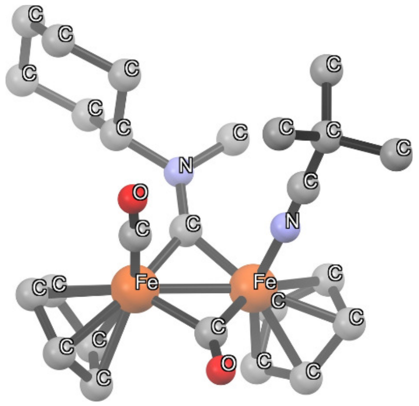

[3E]$^+$

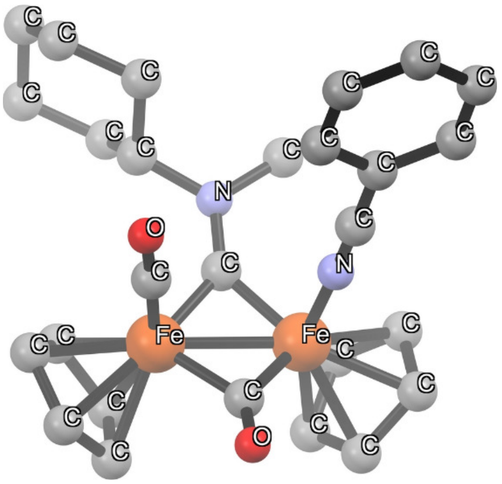

[4E]$^+$

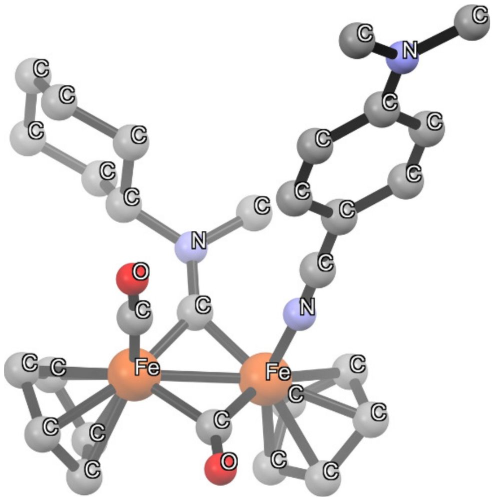

[5E]$^+$

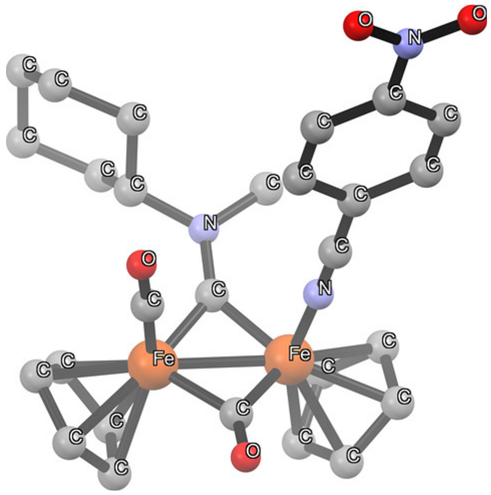

[6E]$^+$

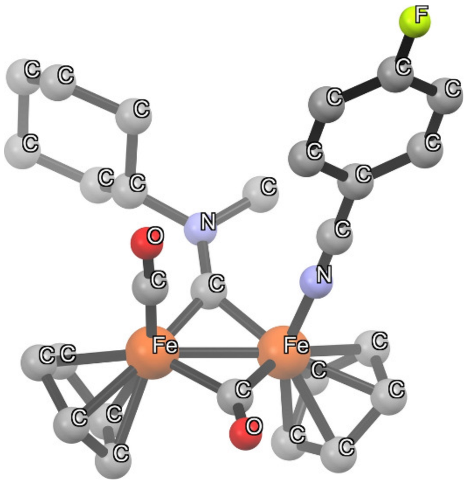

[7E]$^+$

Figure S50. Views of the DFT optimized structures of model complexes. H atoms (expect those ones belonging to the nitrile ligand, if any) have been omitted for clarity.

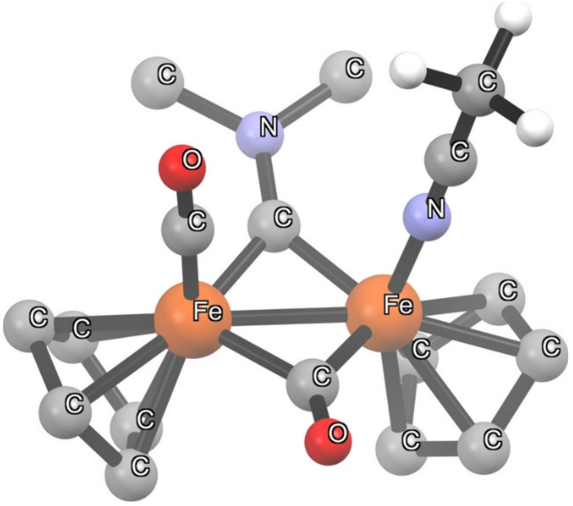

$[Fe_2Cp_2(CO)_2\{\mu\text{-}CNMe_2\}(N{\equiv}CMe)]^+$, **[M1]**$^+$

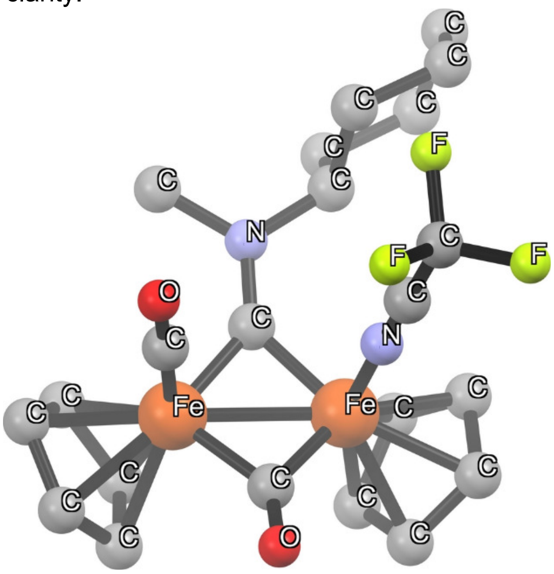

$[Fe_2Cp_2(CO)_2\{\mu\text{-}CNMe(Cy)\}(N{\equiv}CCF_3)]^+$, **[M2]**$^+$

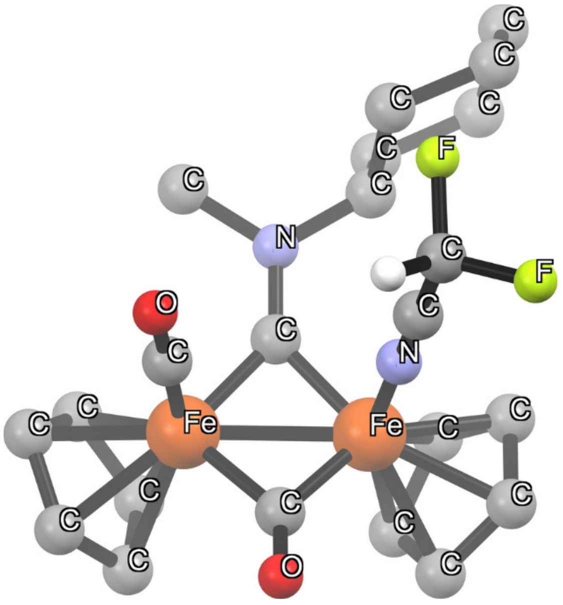

$[Fe_2Cp_2(CO)_2\{\mu\text{-}CNMe(Cy)\}(N{\equiv}CCHF_2)]^+$, **[M3]**$^+$

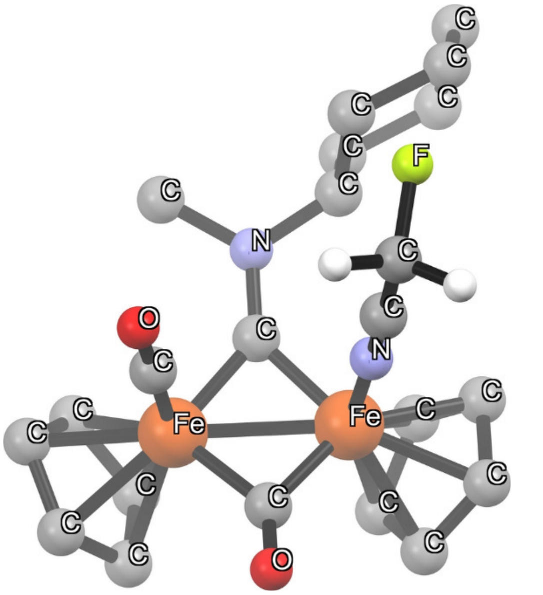

$[Fe_2Cp_2(CO)_2\{\mu\text{-}CNMe(Cy)\}(N{\equiv}CCH_2F)]^+$, **[M4]**$^+$

DFT optimized geometries

[M1]$^+$

Fe	0.43519305187458	2.47399644266526	-0.36779944578611
Fe	0.08209635222908	0.02856040770164	0.02230328438828
C	0.53871048335470	3.03754258499255	1.27694293383594
C	-1.15698154011235	1.46715873913367	0.10815216475674
O	0.64060725895510	3.43207207988877	2.36497223852400
O	-2.29993876756426	1.69212865410754	0.30765300116425
C	1.59465372734851	1.08109042811701	0.05990463704319
N	2.86082291425296	0.96227267016705	0.37734287563163
C	3.79847816025839	2.09776509040171	0.39474069700642
H	4.57647449318994	1.95887002695391	-0.37037083994017
H	4.27879969299566	2.15597385247698	1.38124186500190
H	3.24355840327105	3.01808731069641	0.20454442531130
C	3.46083926756679	-0.32680007892664	0.76484096902148
H	4.05863369401497	-0.73667530513303	-0.06269824899397
H	2.65819779357233	-1.01905074896688	1.02642423599034
H	4.11542235555235	-0.17100019931200	1.63276690192361
N	0.06609041646147	0.01911159572872	1.89127957748764
C	0.01487305977886	0.07431319415998	3.05213365165495
C	-0.06944072718298	0.17510806299059	4.49374432923082
H	0.71779584836382	-0.42236225953144	4.97504000240047
H	-1.04877356871641	-0.18151557057735	4.84416421115651
H	0.04918621170023	1.22614337748287	4.79511555913137
C	1.60898908711939	3.26054462232450	-1.97949513469712
H	2.65388916817051	3.03189526147418	-2.16311182026101
C	1.10739602727164	4.30482128463338	-1.14969134621860
H	1.69957251087107	5.02735777203755	-0.59484616240901
C	-0.33428063900650	4.26228180495729	-1.20051023026250
H	-1.01292487559475	4.92734731298465	-0.67564347211340
C	-0.70232617695332	3.18268181340106	-2.04422333449240
H	-1.71836873175743	2.87195621116955	-2.26580894952105
C	0.49534661536715	2.55870721034115	-2.52200542845913
H	0.55521022206476	1.71025910215180	-3.19445432123488
C	0.72623165518002	-1.11352963896291	-1.61462261773301
H	1.61455498122019	-0.90004602632852	-2.20152250477274
C	-0.59151236448197	-0.61631808526915	-1.87121686044186
H	-0.88496940514949	0.03546430446208	-2.68643747905584
C	-1.47896321465805	-1.15801961358201	-0.88553315006845
H	-2.53926728793996	-0.94381591345798	-0.79303737165191
C	-0.70670348127456	-1.95594950282617	-0.00971045682238
H	-1.07583547232384	-2.45978134873044	0.87909665256374
C	0.65928528932157	-1.94491434680884	-0.45568942660946
H	1.47344751138868	-2.50981257918856	-0.01483561167960

[2Z]$^+$

Fe	0.70629924826351	2.34090196867153	-0.44765616057002
Fe	0.01933963647869	-0.00340101777971	0.08305390232723
C	0.81859846753504	2.98540289101118	1.16530279112230
C	-1.02136350894149	1.57968379360419	0.00892152300918
O	0.92688060297031	3.42612360862340	2.23565620615346
O	-2.13170087772849	1.96526559363175	0.13971074308984
C	1.66042939558427	0.84581743832363	0.12500832451386
N	2.88018362351933	0.58599636736365	0.52122258654119
C	3.95088737797403	1.59795935728622	0.50053632488242
H	4.60698978191993	1.44734105209046	-0.36788199785143
H	4.54831449955042	1.51487616187578	1.41550805832679
H	3.49612596521604	2.59000936986791	0.45208739674981
C	3.24565310255901	-0.76040113053747	1.05698596920884
H	2.32982769504989	-1.34921840220824	0.93787830808965

C	4.36806692762013	-1.41920127143883	0.25126261744765
H	5.29844902275743	-0.83664735949233	0.35878118270796
H	4.11034992480644	-1.42385689734164	-0.81997443514875
C	4.60576781695902	-2.84793883713698	0.76489931778590
H	3.71025468902796	-3.46176348512128	0.56341242542223
H	5.43196485700368	-3.31060078999445	0.20592045356882
C	4.90128916337069	-2.85970073356374	2.27012392342983
H	5.01911422313996	-3.89412712841916	2.62429400912232
H	5.86441702714837	-2.35489362403992	2.45748430382432
C	3.79766617896263	-2.15047041848248	3.06558044258930
H	4.04701253656873	-2.12847597800118	4.13644760454278
H	2.85422708039032	-2.71718924977735	2.97244810367385
C	3.57447416287494	-0.71829561375077	2.55356199836971
H	4.48765208673161	-0.12699094777575	2.73162792760028
H	2.75418064105000	-0.22502208662468	3.09611496601447
N	-0.06299048959328	0.11894353675838	1.94268753902428
C	-0.13188762657961	0.23981872658608	3.09767620663500
C	-0.22140732845251	0.42376686495561	4.53087732425079
H	0.35242205238900	-0.35013914270685	5.06041200206179
H	-1.26955576016610	0.37321197425308	4.85871387265904
H	0.18206958818124	1.41077678843036	4.80031556590508
C	1.05577902475268	2.27112266322033	-2.57100247471292
H	1.21517884941558	1.37459971238657	-3.15966359114756
C	-0.20109485957067	2.90515300993071	-2.30809566151499
H	-1.17355316890307	2.55920222639490	-2.64381776485364
C	0.03643118708630	4.05524164636755	-1.50834487878533
H	-0.71539449820148	4.73854765610925	-1.12593077013308
C	1.45313825045680	4.12760931558074	-1.26587392764297
H	1.95740451345220	4.89684908025134	-0.68762671528814
C	2.07765104638429	3.03357579711306	-1.93582898200036
H	3.14083753571742	2.81977124568341	-1.96151798570689
C	0.68760337222475	-1.57196736434307	-1.17358785966217
H	1.71520916582979	-1.70407960683270	-1.49998983238165
C	-0.31510542961575	-0.81796309149078	-1.85723333365246
H	-0.19295681614563	-0.29868701456139	-2.80095488348688
C	-1.53292688073744	-0.90887978509327	-1.10682871752588
H	-2.47442396216777	-0.42932139255356	-1.35569728579067
C	-1.27724021720271	-1.68316746311929	0.04779119931845
H	-1.98695664189152	-1.90188736019404	0.83989307978366
C	0.10411391371068	-2.09924326400696	0.01281040067330
H	0.59578383126434	-2.71868738998322	0.75658865743038

[2E]$^+$

Fe	0.73442712544901	2.30319519936636	-0.51481542951658
Fe	0.01913895005467	-0.03921134806939	-0.01695443328923
C	1.01203297745169	2.89055190503532	1.10004601964891
C	-0.97060291760558	1.57304935855659	0.07359860303187
O	1.23028549602494	3.30016097679823	2.16615308960771
O	-2.04858260374514	1.99088097475910	0.32203387391135
C	1.68336771171307	0.75540939310326	-0.08792199839675
N	2.92539841435926	0.44374314143275	0.17890184150947
C	3.31628070622618	-0.93090272583891	0.53656599618062
H	3.96305619418408	-0.91551293476733	1.42219286601980
H	3.85869025021447	-1.40480849346888	-0.29284296368110
H	2.40935469864007	-1.49715567215145	0.75755399017409
C	4.01723218010247	1.46587789310902	0.14532600394049
H	3.49945372624930	2.39722628274011	-0.11585192719115
C	4.67004065470620	1.66681684952264	1.51680524808771
H	5.19047877177962	0.74774356171809	1.83231318247355
H	3.89707424715900	1.88724497239654	2.26781219350811
C	5.69471918508178	2.80951970799134	1.43436546222709
H	5.16697822611253	3.75524760003734	1.21995971269614

H	6.18014562801592	2.93951962179789	2.41232041221514
C	6.74011969361610	2.54625890282354	0.34393718330454
H	7.44108115706726	3.39114659998488	0.27735034391576
H	7.34021880164224	1.66150532218640	0.62111365568754
C	6.07888094930641	2.29972937857523	-1.01774675958951
H	6.83438573874810	2.06728195820593	-1.78140497746137
H	5.57256413293044	3.22445595939785	-1.34854392976698
C	5.05169833416178	1.15958614671736	-0.94006898555142
H	5.57553401730229	0.21702555216188	-0.70904272110417
H	4.54630317667124	1.01625502465924	-1.90908999451805
N	0.11934921445337	0.00478695639175	1.84573555001980
C	0.17467794983513	0.08831599958081	3.00481030927166
C	0.22425623218017	0.22528516179068	4.44529135261245
H	0.85403530141842	-0.55785182777765	4.89108658760635
H	-0.78692612682854	0.15111699433355	4.87070161684853
H	0.64200581059569	1.20842282245727	4.70713279354437
C	0.86528778708179	2.31162775358257	-2.66638291838320
H	0.93406031706023	1.43663749514588	-3.30310477246240
C	-0.33527569426107	2.97340334429028	-2.25194952332366
H	-1.34865736186195	2.67119139205888	-2.49610477076908
C	0.02291074704830	4.08036478431576	-1.43677379725450
H	-0.66168154449399	4.76958522858487	-0.95260691169058
C	1.45918248834943	4.09741800291153	-1.33882932996883
H	2.04791262211480	4.82361576804003	-0.78537923122279
C	1.97219828342417	3.01454416704495	-2.11248117816065
H	3.01893131615654	2.76988603786464	-2.25958739824501
C	0.50763347179998	-1.52661732509676	-1.44244298532495
H	1.48251395197055	-1.64006413315224	-1.90661287348744
C	-0.56820610514400	-0.72882423561803	-1.94189131291655
H	-0.56146662395452	-0.14883741289118	-2.85788404633055
C	-1.68233068052269	-0.86794231407119	-1.05093556482706
H	-2.64257682172538	-0.37088420879907	-1.14558595508775
C	-1.28672875320904	-1.71402946253483	0.00998415079852
H	-1.89072802637723	-1.98020209124407	0.87210644710524
C	0.07530499096248	-2.12825332449740	-0.22587626151146
H	0.64656163030768	-2.80353668149205	0.40351446508599

[3E]+

Fe	0.76093733092635	2.40345925501804	-0.44407162066850
Fe	-0.14447359791189	0.10206074689531	-0.09412128059693
C	0.91728553612272	2.96543524873639	1.19447989614140
C	-1.03658606890246	1.76856374053067	-0.02153193796871
O	1.06289314394024	3.37816795904895	2.27204533134150
O	-2.10120928863259	2.25663511513765	0.14065870795815
C	1.56214876603137	0.78924714872422	0.01905112020697
N	2.74236292593764	0.38364278197355	0.40912231649850
C	2.99937346331629	-1.03052240371260	0.73570000044789
H	3.71424134417632	-1.09157526553347	1.56420840899851
H	3.41719422643149	-1.55805003648961	-0.13252529956909
H	2.05600220055746	-1.49423623349925	1.03235014337097
C	3.88143256940039	1.33351347205235	0.59581868060203
H	3.48874021269008	2.29305991768365	0.23792752600601
C	4.23669061513588	1.50093536702032	2.07791836361115
H	4.58973024118573	0.54465136226953	2.49760455385246
H	3.33775054081853	1.79746919253671	2.63900527719974
C	5.34520368307107	2.55393907689773	2.22920038838210
H	4.95558831674426	3.53712897655673	1.91253435943824
H	5.61568603030964	2.65499895535549	3.29014006677649
C	6.57524459079900	2.19580624679037	1.38766391225629
H	7.34075973723967	2.98002886083243	1.48136600782014
H	7.02755257554382	1.26848604092821	1.77981729273186
C	6.20663835992227	1.99360178350457	-0.08758671503274

H	7.08876286557315	1.68969056442300	-0.66931650608688
H	5.86662033019608	2.95329245586778	-0.51587537069782
C	5.09622840494499	0.94398527297010	-0.24709295118305
H	5.47723338006271	-0.03758943531879	0.08041690249604
H	4.80230995422075	0.83689688588814	-1.30382222445905
C	1.08983834853028	2.42579620660505	-2.57207097879124
H	1.14164699841125	1.55305532644372	-3.21403963753337
C	-0.08395808932905	3.18326145284742	-2.25531851637473
H	-1.09298486857509	2.97185492102463	-2.59520485529833
C	0.28951726421445	4.24548335753060	-1.39022041222581
H	-0.37549175253576	4.98666164496151	-0.95836340283354
C	1.70722348371058	4.13778828220342	-1.16041055682121
H	2.30089047105394	4.80424306054760	-0.54122991610577
C	2.19673298035369	3.02379911345948	-1.90415775983746
H	3.22700484800413	2.68633946297164	-1.94996131942425
C	0.34177754097780	-1.27903267914170	-1.61354935107669
H	1.32192948656800	-1.37178362835662	-2.07088325066440
C	-0.71724556075943	-0.43265258526466	-2.06774191710983
H	-0.69096328747386	0.21841155484539	-2.93477141219320
C	-1.84673912055992	-0.62349404007945	-1.20594331603067
H	-2.79768939304397	-0.10484017384305	-1.27507210917178
C	-1.47617257730574	-1.55169892305432	-0.20692072255264
H	-2.09448796608696	-1.86914366704606	0.62741754558689
C	-0.11825332089600	-1.96805394528451	-0.45312690408898
H	0.43431331684696	-2.70011762600938	0.12664497351251
N	-0.15238862131100	0.04465939648309	1.77184920185620
C	-0.09823309857519	0.02253453482683	2.93461907394751
C	-0.01118187044461	-0.02348571964189	4.39774609074431
C	-1.21505362188088	-0.83146503262891	4.93560062375471
H	-2.16621229617383	-0.35705062160158	4.66009725327240
H	-1.15290690646010	-0.87287406094365	6.03199075359857
H	-1.20870631279095	-1.86004101597406	4.55096401753925
C	-0.04367319808869	1.42472480544843	4.93749687430590
H	0.79515646911706	2.01662903872226	4.54942942822753
H	0.02741654310746	1.39180643165707	6.03382400504483
H	-0.97995956005862	1.92987523026755	4.66532203036871
C	1.32004337514916	-0.71321484533199	4.77995458247785
H	1.36570209523511	-1.73809735071647	4.38780850132652
H	1.39641244153501	-0.75949754337732	5.87505922060264
H	2.18100336968431	-0.14756341563650	4.39865681213162

[4E]$^+$

Fe	0.69968232139506	2.30086123302583	-0.52124083522523
Fe	-0.02772854545227	-0.03956244565040	-0.03286295388101
C	0.95889190291182	2.90422176722804	1.08942549330723
C	-1.01200503930586	1.57698077677656	0.06022515119402
O	1.16307736276089	3.33752645773665	2.14944609935943
O	-2.09017048007220	1.99902490087570	0.29713637668675
C	1.63869859221114	0.75269911223940	-0.07917522688743
N	2.87211074626380	0.43851858642498	0.21874818493911
C	3.25054775275953	-0.93653173628200	0.58830121841959
H	3.85356852928843	-0.92304020087913	1.50408455766629
H	3.83125537822834	-1.40486234237649	-0.21775145547094
H	2.33616577547806	-1.50544945487761	0.76676446208674
C	3.96357274582676	1.46115618913999	0.22371005384510
H	3.45282682577702	2.39399374537462	-0.04628036673427
C	4.57604587765885	1.64890891346156	1.61519134903731
H	5.09091105437136	0.72785576274349	1.93399827462526
H	3.78204531221360	1.85566146219243	2.34792140961843
C	5.59896667790213	2.79499174132821	1.57386654001828
H	5.07505540918937	3.74224196072824	1.35632981365552
H	6.05680222299040	2.91515367875209	2.56636617405679

C	6.67547705082554	2.54543185739099	0.51095560732129
H	7.37441245322602	3.39388006792788	0.47145634342229
H	7.27193147056354	1.66135886574654	0.79798209016805
C	6.05524714187425	2.30820185118389	-0.87174285838743
H	6.83377429465511	2.08485955756662	-1.61476892300097
H	5.55657373077400	3.23483308961109	-1.20919905418957
C	5.02858358444048	1.16545047602211	-0.83423100048873
H	5.54719522129198	0.22127608141984	-0.59802058921702
H	4.55079357964704	1.03038671588968	-1.81832671844387
N	0.08920712318644	-0.02864860323196	1.81992196927700
C	0.19845832451676	0.00311939975328	2.98252765987043
C	0.89293640182318	2.29443958372622	-2.66656520064103
H	1.00018087545444	1.41411036464673	-3.29079244870925
C	-0.33443645881267	2.93150239514602	-2.29368239658196
H	-1.33299043634556	2.60353506058731	-2.56413099980441
C	-0.02670816346221	4.05442363784250	-1.47936829416226
H	-0.74148279028020	4.73003781613584	-1.02031754134410
C	1.40425041377762	4.10606562269613	-1.33938927267100
H	1.95917828528404	4.85050567924973	-0.77525391796785
C	1.96633311499545	3.02808640929477	-2.08642650561456
H	3.02272823953071	2.80734707696063	-2.19916529290469
C	0.46550371915036	-1.52316585959804	-1.46397662425182
H	1.44453730828877	-1.64000072037009	-1.91859262678139
C	-0.60154316964480	-0.71794125929043	-1.96930087516992
H	-0.58351070488040	-0.13454928251410	-2.88305543925515
C	-1.72384692874382	-0.85438785026011	-1.08805542299991
H	-2.68047451269247	-0.35150185954596	-1.18864534414075
C	-1.34263903420410	-1.70761298469728	-0.02743356566887
H	-1.95459810187447	-1.97155734765369	0.82970946619837
C	0.01890701478784	-2.12749548810855	-0.25413152707833
H	0.58297432256938	-2.80572040422252	0.37863745679578
C	0.34240323355725	0.05973976402948	4.39059873629796
C	0.63312898950514	0.18775781967741	7.16284675062525
C	0.21432415686841	-1.11372937562999	5.16477334786929
C	0.61542823366445	1.29881401133053	5.01064818836263
C	0.75953031476830	1.35054229542140	6.39443305178545
C	0.36047716900535	-1.03941118307686	6.54716210776481
H	-0.00170872803067	-2.06491776558110	4.67845013665928
H	0.70866779481807	2.19841568863142	4.40334723047698
H	0.96870080412191	2.30539108311544	6.87752082315300
H	0.25933278894406	-1.94310483750393	7.14877172718971
H	0.74544145065910	0.23788244231889	8.24662542592023

[5E]$^+$

Fe	1.81254841788588	1.60304166034020	-0.36513404613825
Fe	0.05352576851955	-0.12747602256602	0.02352386036907
C	2.19651613517791	2.05026637590216	1.26984946901727
C	-0.08306686404578	1.75417721996550	0.09518104079161
O	2.49634112803925	2.37951002402649	2.34491817072381
O	-0.85107692472655	2.63621640814382	0.27020292076296
C	1.89125250661952	-0.20097121836605	0.08964050710186
N	2.81352096434754	-1.05104514320614	0.45914201423395
C	2.47602541790253	-2.43659900274740	0.83444558133399
H	3.12493494227080	-2.76543875120744	1.65444462499922
H	2.61084667802961	-3.11182115822290	-0.02148047080624
H	1.43596047214806	-2.45785691622825	1.16610527130892
C	4.24610215489254	-0.64833312031467	0.58993193856916
H	4.27945811716821	0.36647280755514	0.17488117203999
C	4.67495479703095	-0.57114335759248	2.05999012716443
H	4.61785386805866	-1.57050323096519	2.52380801269405
H	3.98825301010908	0.08513734814288	2.61311119676492
C	6.12136120502532	-0.06436576498533	2.15519788705325

H	6.16673611364016	0.97432359989101	1.78442404194796
H	6.43365740928189	-0.03270023036212	3.20925459925752
C	7.07475859475330	-0.94321174100997	1.33585453154468
H	8.09864789994577	-0.54511963651228	1.38666654533907
H	7.10719297333194	-1.95396441625600	1.77854224710527
C	6.62071734378339	-1.04644500054856	-0.12578003448979
H	7.28617511620948	-1.71213631649834	-0.69409320342746
H	6.69220488723932	-0.05227114532489	-0.60223686240083
C	5.17284598425491	-1.55050281087090	-0.22840197322716
H	4.84041323236465	-1.57940033954061	-1.27854743333143
H	5.12035793619011	-2.58321154085700	0.15414652547425
N	0.08257889737028	-0.17819253576564	1.89154631057816
C	0.19852424446782	-0.22779348219048	3.05483315936847
C	0.33921659140388	-0.28104383500564	4.45323637295101
C	0.95692289495689	0.78322482439905	5.15305342458624
H	1.31925162597472	1.65058004412959	4.60153667459280
C	1.10259018786887	0.73274511866434	6.52585375739524
H	1.57604156320364	1.57243806912188	7.03009387689670
C	0.63728581811878	-0.38492191085424	7.27740048768160
C	0.01098337334781	-1.44626833514805	6.56205893608025
H	-0.37280780888094	-2.31399047181413	7.09384563304866
C	-0.13106827422198	-1.39481216174588	5.18894587737557
H	-0.61702016455080	-2.22029231272705	4.66724531465782
N	0.78612319492582	-0.43771256249140	8.63373822767462
C	1.37671392822517	0.68673683715560	9.35282050859648
H	2.39865426264411	0.89400782256883	8.99919933957389
H	1.43142322086372	0.43797106494166	10.41697953020404
H	0.77319889406675	1.60328925122393	9.24343116013929
C	0.25797338129050	-1.56772007354864	9.39100295843441
H	-0.84097558752583	-1.62973605102181	9.32160976238656
H	0.52939894432806	-1.44617669495599	10.44412997785632
H	0.68634636125982	-2.51854005078599	9.03898247430402
C	2.25934420234818	3.47875312425692	-1.23851769710685
H	2.08182486970705	4.42905190779521	-0.74531357292017
C	3.45031843015975	2.67127225100350	-1.12586921388946
H	4.33494659167653	2.92191036235616	-0.54698482260010
C	3.27131064137969	1.50645367071576	-1.92717375388265
H	3.98716142493815	0.70146814436801	-2.05912170421379
C	1.97440960195026	1.57392267443152	-2.51409218938920
H	1.54344037382669	0.82861437643719	-3.17314546852368
C	1.35444111243197	2.79360356379099	-2.09015822495181
H	0.35537073940278	3.12811828211447	-2.35067612286901
C	-0.11392721720923	-1.55762492212480	-1.51313500639344
H	0.73298883042950	-2.00923795851027	-2.02004275504681
C	-0.78726208968599	-0.36007791160707	-1.91124886559063
H	-0.55696265699147	0.24807588664476	-2.77872789308595
C	-1.85941459693903	-0.11847178389655	-0.99056778775572
H	-2.53711556023540	0.72949932369266	-1.00766048074749
C	-1.82432420669193	-1.13614441155622	-0.01143080233532
H	-2.46922559327929	-1.20212299487426	0.85994618501104
C	-0.74664069961228	-2.03870763351325	-0.32856282148279
H	-0.49205903219130	-2.93777708545936	0.22235097161540

$[6E]^{+}$

Fe	0.69063353379871	2.29112488056292	-0.49767767268333
Fe	-0.02814937820676	-0.05798137023422	-0.02673190952765
C	0.94861262939956	2.87905764024973	1.11905317202565
C	-1.01759258771418	1.56217819033543	0.07181946219897
O	1.15118812346040	3.29941354759002	2.18443034900330
O	-2.09859597891503	1.97267787871402	0.31318408312075
C	1.63848705753008	0.74359652757127	-0.07379967717938
N	2.87287586490554	0.42930105529239	0.21586312870259

C	3.25866879505453	-0.95019263059435	0.56141840937221
H	3.87505064783529	-0.94695209854121	1.46834568939554
H	3.82977019688261	-1.40566888599466	-0.25866649142902
H	2.34838639016182	-1.52433169096807	0.74457045977199
C	3.96209782899087	1.45621335458504	0.23676245115557
H	3.44930574438518	2.39023256698283	-0.02473582446810
C	4.56605401418938	1.62994526660985	1.63376872586173
H	5.08152992779473	0.70711223556166	1.94616531946864
H	3.76694779605954	1.82690207839470	2.36388247530531
C	5.58520396506460	2.77989159308295	1.61115402627897
H	5.05908081654718	3.72792275209277	1.40235749817274
H	6.03727480547749	2.88951446171310	2.60739361939072
C	6.66780312188149	2.54619951901051	0.55098053335548
H	7.36377023065205	3.39748478412026	0.52490565161009
H	7.26610470436607	1.66120068797585	0.83097171755437
C	6.05574884476642	2.32281014905727	-0.83761894629459
H	6.83896393881625	2.10986896434131	-1.57867713324890
H	5.55634285513103	3.25164167707173	-1.16803649737094
C	5.03298034640896	1.17594230022793	-0.81901549856442
H	5.55453730630735	0.23130308157614	-0.59167484612155
H	4.56077009430597	1.05091478582097	-1.80724835304695
N	0.08226042316160	-0.05555621685694	1.81586178047624
C	0.17860249428691	-0.02336393351128	2.98047824956364
C	0.88522310855302	2.31084142451992	-2.64306701028006
H	0.99344346508947	1.43921745865960	-3.27919280788929
C	-0.34273702720697	2.94265458894262	-2.26321064565477
H	-1.34092966575262	2.61846682229297	-2.53961293406102
C	-0.03583755399814	4.05570385257044	-1.43494861916815
H	-0.75105167359993	4.72640530041093	-0.96934687218335
C	1.39511205088560	4.10672686927859	-1.29408048864912
H	1.94952418264031	4.84506743110309	-0.72145520925051
C	1.95781305260350	3.03790099891962	-2.05360305278811
H	3.01434512738649	2.81929082655990	-2.16893759297954
C	0.47445192180754	-1.52900431698962	-1.47027257466306
H	1.45516502057655	-1.64199093781095	-1.92216331411126
C	-0.59033511814986	-0.71936104945600	-1.97301835089293
H	-0.56794935565943	-0.12774332503368	-2.88131413803179
C	-1.71656814585054	-0.86373587213047	-1.09825865880264
H	-2.67364500935640	-0.36182611340987	-1.19959261610394
C	-1.34030760212366	-1.72805315139457	-0.04427104004811
H	-1.95776272041980	-2.00321452838928	0.80543530537406
C	0.02214688708915	-2.14524487170036	-0.26864815245493
H	0.58389904160818	-2.82920810873141	0.36012831477095
C	0.31065266542003	0.03940541681251	4.38872178914680
C	0.58560491836629	0.17954596486862	7.13275684460152
C	0.15884258794702	-1.12831757387005	5.16944047969128
C	0.59812989554432	1.28003093043175	5.00186083412002
C	0.73618965395313	1.34615274878369	6.38333869028729
C	0.29735284182734	-1.05471897580624	6.55047786791535
H	-0.06837951642478	-2.07992167462414	4.69013208780099
H	0.70835149969314	2.17498037174665	4.39120093714307
H	0.95680313749970	2.28444516420909	6.89011645351588
H	0.18668842984620	-1.93356488786708	7.18402843707777
N	0.74320577266605	0.25343781390548	8.61775049984445
O	1.00939869010781	1.35755839974298	9.10052223093332
O	0.59844488464548	-0.79533014838476	9.25159935394006

[7E]⁺

Fe	0.70521495946439	2.29883431356965	-0.49714872036007
Fe	-0.01438517127656	-0.04777443041627	-0.02876918685662
C	0.96897484452401	2.89089722939474	1.11615650615013
C	-1.00180094359557	1.56677045806606	0.08035768356836

O	1.17254598206182	3.31682888800229	2.17962638380177
O	-2.08049684602821	1.98298343157809	0.32550666232623
C	1.64924034939927	0.74869566392570	-0.07056076408443
N	2.88343676763333	0.43447201725951	0.22342232683653
C	3.26607203258919	-0.94654744050632	0.56590491376737
H	3.90529499991233	-0.94412372174020	1.45674556318171
H	3.81199664664703	-1.41143106559898	-0.26604232284534
H	2.35406048757753	-1.50921338098468	0.77391312372982
C	3.97650849331900	1.45630264438907	0.22111491513283
H	3.46170805494570	2.39156712724120	-0.03215612323475
C	4.60949973831698	1.63150104104095	1.60495725176096
H	5.12500526467490	0.70663025277473	1.91165378567032
H	3.82678365182193	1.83719855960253	2.35004231303892
C	5.63694249680553	2.77344468148177	1.55618522065164
H	5.11484053373804	3.72345605138877	1.34604072448976
H	6.10555968354971	2.89019721223904	2.54406218806244
C	6.69891920663569	2.52212806805759	0.47950133766136
H	7.40768628977640	3.36235589640549	0.43904963881554
H	7.28868871972766	1.62897339260207	0.75208733234720
C	6.05812372514140	2.30335288870832	-0.89661445519103
H	6.82352471984813	2.08383012716382	-1.65423085458393
H	5.56003799834797	3.23656942133537	-1.21665894071988
C	5.02451211072211	1.16733223220482	-0.85607941340547
H	5.53877557783933	0.21620805370576	-0.63950117685328
H	4.53080420903676	1.04734644717855	-1.83456414404486
N	0.09573366111258	-0.04538040380877	1.82401807152643
C	0.19507874479333	-0.00775554872828	2.98758700604062
C	0.89606507953497	2.30892254216983	-2.64464417508859
H	1.00810747893616	1.43260929230775	-3.27373478000307
C	-0.33347988245982	2.93780939936947	-2.26570547673118
H	-1.33100495217213	2.60687069912831	-2.53656438341385
C	-0.03042012638205	4.05653874039375	-1.44410549896204
H	-0.74776813142215	4.72609850580224	-0.98019175894333
C	1.40000495204348	4.11343307943809	-1.30492460918862
H	1.95160863706132	4.85617925420122	-0.73521019576194
C	1.96650211991384	3.04318114068476	-2.05987891962479
H	3.02391374740431	2.82818193667047	-2.17391334465108
C	0.46636389186488	-1.51505560600817	-1.48212354620109
H	1.44211045299604	-1.62258462506194	-1.94604845255233
C	-0.60611570876040	-0.70479152126755	-1.96776218363559
H	-0.59809962888058	-0.11002949163091	-2.87437327464842
C	-1.72052312108676	-0.85467092005715	-1.07860147762452
H	-2.67861870911904	-0.35160806117175	-1.16352839972633
C	-1.32816192997721	-1.71918440676871	-0.03170072908208
H	-1.93156943825223	-1.99437939441757	0.82801119078989
C	0.03224138036903	-2.13346018422146	-0.27484572413803
H	0.60169056548370	-2.82108421704078	0.34282677695432
C	0.32083391939816	0.06327857014678	4.39502517986486
C	0.56760718683544	0.22228432248225	7.14519594246397
C	0.26169590767467	-1.11146873470898	5.17728744869919
C	0.50455253400924	1.32024066179588	5.01428235161731
C	0.63028508138933	1.39830842230843	6.39617547445818
C	0.38469476692887	-1.03140964916543	6.55932521045556
H	0.11495875570154	-2.07876467634265	4.69748161356945
H	0.54843310618861	2.22278638568779	4.40617942438917
H	0.77122975543020	2.35379914407612	6.90040914016415
H	0.33965503669612	-1.92048836038117	7.18721401163663
F	0.68832028359061	0.29880764404861	8.48483631853390

[M2]$^+$

Fe	0.71831901659827	2.35673212675592	-0.47320441432354
Fe	0.01197382343487	0.02714940330775	0.10730998121627

C	0.84712390674251	3.04459747859589	1.12661531413425
C	-1.01086382720677	1.64400562823374	-0.00656449125109
O	0.96735611381311	3.51657732076218	2.17867337285207
O	-2.12142180032116	2.02437482806838	0.11884669069141
C	1.66949346415548	0.87255512256203	0.12450999742317
N	2.88247540789737	0.60223041399973	0.52380721159657
C	3.96474019246055	1.60391619825207	0.49821286485431
H	4.62027763866460	1.43606032401958	-0.36681546223754
H	4.55651174699403	1.51905160275959	1.41614940368852
H	3.52238702521227	2.60078608536142	0.44123212788052
C	3.23965843045821	-0.74787516320761	1.06410396195944
H	2.31585933254740	-1.32667735738193	0.95951523071987
C	4.34249029813423	-1.42185755345521	0.24433413650656
H	5.28136287740962	-0.85144078571029	0.33879011214861
H	4.07130980700667	-1.42641943130375	-0.82362753201718
C	4.56736359260899	-2.85264304122719	0.75953778991015
H	3.66243595043004	-3.45567171590552	0.56840469333738
H	5.38150470578214	-3.32586439070457	0.19212059190637
C	4.87895049765572	-2.86554758500746	2.26157709011373
H	4.98307063257127	-3.90115585469443	2.61583838402225
H	5.85238274444573	-2.37688296985624	2.43703578474002
C	3.79688799771815	-2.13666341877978	3.06830009124699
H	4.05897815377253	-2.11231682440355	4.13539671251613
H	2.84309935641619	-2.68800948701479	2.99221318580605
C	3.59055047953329	-0.70210947060236	2.55499419830697
H	4.51825948497640	-0.12908561610915	2.71517756401334
H	2.79186544908463	-0.19427752879393	3.11387103566218
C	1.09173097893103	2.24992874471342	-2.59046219650469
H	1.25913325890077	1.34476664483629	-3.16359482096824
C	-0.16846268289039	2.88720071136356	-2.35229613389827
H	-1.13704320098817	2.53654574624676	-2.69439446593720
C	0.05967628187778	4.05236420195492	-1.57058892900973
H	-0.69670570582376	4.74209700677140	-1.20901056463966
C	1.47302616074896	4.13113144054441	-1.31559006580340
H	1.97071120858916	4.91082326359793	-0.74566923616592
C	2.10553049146806	3.02551820968200	-1.95874809717595
H	3.16908633800612	2.81273907844200	-1.96899005301120
C	0.66946763313320	-1.55974149611687	-1.14128665449509
H	1.69705348351992	-1.70783396193177	-1.46028635357505
C	-0.31995356471427	-0.79708446568100	-1.83393558406940
H	-0.18593109397073	-0.28583340548186	-2.78021298202144
C	-1.54312201767115	-0.86630680466070	-1.09020362701284
H	-2.47765114707177	-0.37798956195467	-1.34792348260261
C	-1.30421578285057	-1.63916710833276	0.06933354217933
H	-2.02160620767000	-1.84711809022620	0.85777061620932
C	0.07220026739103	-2.07245753583980	0.04410494958823
H	0.55326620477137	-2.69138475753685	0.79543226389665
N	-0.07037908017963	0.15190723820454	1.91837767567294
C	-0.14339563075115	0.21705573468428	3.08197156434327
C	-0.22861895860709	0.28750429130468	4.55293933366151
F	1.00208686627844	0.12538624884658	5.10209982027736
F	-1.03569636805598	-0.69057469041773	5.01797679976967
F	-0.71511023136770	1.48233497846689	4.94092105386861

$[M3]^+$

Fe	0.71501455630562	2.35754013127738	-0.47189915232317
Fe	0.00826804396147	0.02594798605598	0.09725112356612
C	0.82016118177131	3.03303990824193	1.13171621894706
C	-1.01889836442256	1.62899019572174	-0.01697573056513
O	0.91612701875759	3.49243847565542	2.19360920732893
O	-2.12874435229311	2.01566204851187	0.10300212745750
C	1.66427325837281	0.87199195375565	0.12556605346237

N	2.87545732865479	0.60347008079042	0.53194988587678
C	3.95489111249879	1.60795850884115	0.51484841593954
H	4.61823534732831	1.44236507718628	-0.34492501475954
H	4.53957157352406	1.52454326055738	1.43758097928711
H	3.50976875743374	2.60359745903800	0.45404954862201
C	3.23282907632299	-0.74700865940418	1.07129955293442
H	2.30831595024220	-1.32479827532143	0.96751617665322
C	4.33360026228906	-1.42056747560410	0.24825505381389
H	5.27336585519515	-0.85112536438274	0.34233330723281
H	4.06063588876410	-1.42289501617843	-0.81942465476596
C	4.55837865067344	-2.85250388225313	0.76011144327608
H	3.65207923641781	-3.45419478398558	0.57018093002569
H	5.37064903958201	-3.32574085166934	0.18973857966233
C	4.87287626099394	-2.86833990292736	2.26157340257224
H	4.97722584458124	-3.90471355246930	2.61393833326623
H	5.84723487452763	-2.38039468340964	2.43541711962617
C	3.79302519955113	-2.13979106044319	3.07135625326163
H	4.05651870349189	-2.11718769796837	4.13821875487228
H	2.83823208047237	-2.68970023233502	2.99626298434873
C	3.58648769904814	-0.70411837190694	2.56156685298885
H	4.51523302775981	-0.13200947684040	2.72084118614668
H	2.78872362696878	-0.19869919951552	3.12400542096044
C	1.08807082058511	2.25512656679582	-2.59006252895942
H	1.25208706905536	1.35049914578889	-3.16503259200326
C	-0.16963986393772	2.89710608295870	-2.35098524629798
H	-1.13947636655730	2.55088718011454	-2.69413859353545
C	0.06287589533090	4.05859700870199	-1.56559028121477
H	-0.69078505710813	4.75121347889556	-1.20379904819147
C	1.47682871619825	4.13035395645362	-1.30865672263146
H	1.97743621588903	4.90636932995627	-0.73627235991130
C	2.10490851267715	3.02419622238497	-1.95512173063703
H	3.16749134369801	2.80621108693766	-1.96465971964377
C	0.67419667872716	-1.56290771131616	-1.14401975731961
H	1.70294983530686	-1.70879759943025	-1.46038633495189
C	-0.31592464051610	-0.80524504582222	-1.84130468394369
H	-0.18178950262092	-0.29582148797580	-2.78868033716930
C	-1.54022443651564	-0.87581514449904	-1.09937470419317
H	-2.47562074249459	-0.39091348590337	-1.36059861290128
C	-1.30138998691195	-1.64416684457968	0.06357685449964
H	-2.01941089890023	-1.85207991217369	0.85143732681557
C	0.07615447018496	-2.07388758117360	0.04199113775615
H	0.55716095994027	-2.68892348550246	0.79653235127183
N	-0.07088445706731	0.15553549709362	1.91638037080693
C	-0.11786753654652	0.23417742461017	3.07943724979982
C	-0.20440700108556	0.35573052985378	4.54403781872086
F	1.04543211561597	0.16938865138180	5.08973732406103
F	-1.04195781415861	-0.61135640501630	5.02966740754035
H	-0.57627106756321	1.34611594244660	4.85101105251731

[M4]$^+$

Fe	0.69760053429386	2.34855477111040	-0.46301492106808
Fe	-0.00093000692692	0.01011813589058	0.08338927644090
C	0.78943354507673	3.01292364125117	1.14491288335639
C	-1.03489726670140	1.60707029271044	-0.02707042178022
O	0.87898154239965	3.46814854911244	2.20976902211231
O	-2.14796780166112	1.98816983158506	0.08634537733803
C	1.64719612509383	0.86016305811408	0.13221753937728
N	2.85843052337050	0.59844767792647	0.54491293221300
C	3.93005007275043	1.61077688354293	0.54248930540060
H	4.60095714398268	1.45759927838450	-0.31387755745586
H	4.50921191364764	1.52485025708768	1.46845934370261
H	3.47781041592965	2.60349330812906	0.48645167231003

C	3.22167679745255	-0.75194508776370	1.07948780386637
H	2.30088173100074	-1.33478687652002	0.97003755355009
C	4.32933336941580	-1.41538417896610	0.25717942587567
H	5.26522650822891	-0.84048948485024	0.35693791219239
H	4.06009352395308	-1.41484875774004	-0.81147890520674
C	4.56162436683640	-2.84805335034437	0.76346701551230
H	3.65913948537783	-3.45402840553078	0.56876846046810
H	5.37813071461246	-3.31432046556403	0.19329694616626
C	4.87184084268998	-2.86829409683297	2.26582508338031
H	4.98217124419765	-3.90552085550529	2.61408866351419
H	5.84250175810959	-2.37466777226213	2.44449317765696
C	3.78477966355524	-2.14994456210702	3.07495489700326
H	4.04363726973497	-2.13020499259356	4.14299251558908
H	2.83355430363059	-2.70499237838285	2.99373337726215
C	3.57044656046010	-0.71364953319544	2.57093952748792
H	4.49526560220148	-0.13641906328173	2.73523387534451
H	2.76750961979089	-0.21783138364122	3.13413463640185
C	1.09073629358378	2.26140226042264	-2.57884275113863
H	1.26491186714345	1.36051569450509	-3.15672120509840
C	-0.17288949800667	2.89410193590948	-2.34711318109710
H	-1.13749180341286	2.54369776791274	-2.70056268766641
C	0.04549407774198	4.05232673773375	-1.55285123071360
H	-0.71563537196265	4.73806631531095	-1.19369645517086
C	1.45657105771145	4.13099618873243	-1.28280993158128
H	1.94750065870494	4.90645684750040	-0.70138682548275
C	2.09722203815407	3.03263761385166	-1.93028138324163
H	3.16110077289466	2.82103205175976	-1.93178613055753
C	0.68091239407458	-1.56903939954679	-1.15684325905123
H	1.71316737618184	-1.70806198814330	-1.46472308293039
C	-0.30704935405350	-0.81365661112981	-1.85941552510034
H	-0.16748056830354	-0.29931607379093	-2.80338154443803
C	-1.53661851973626	-0.89308001341023	-1.12736281900154
H	-2.47189019668659	-0.41085124073744	-1.39388556427132
C	-1.30333687010626	-1.66498606930290	0.03446920061331
H	-2.02702660127715	-1.87861183286710	0.81535007096821
C	0.07561165286238	-2.08815251616394	0.02252820329392
H	0.55448062703192	-2.70312950141467	0.77841066617684
N	-0.09045871289654	0.14053418830612	1.91435203421840
C	-0.13732405662976	0.25341636378559	3.07426736203680
C	-0.14321274776823	0.38482879867518	4.52470103788590
F	1.04017886091733	-0.13930091221149	5.06475863535389
H	-0.99785590840695	-0.16165821560909	4.95268979898969
H	-0.21382757025944	1.45024717015856	4.795150148992
